# Targeting the MR1-MAIT cell axis improves vaccine efficacy and affords protection against viral pathogens

**DOI:** 10.1371/journal.ppat.1011485

**Published:** 2023-06-29

**Authors:** Rasheduzzaman Rashu, Marina Ninkov, Christine M. Wardell, Jenna M. Benoit, Nicole I. Wang, Courtney E. Meilleur, Michael R. D’Agostino, Ali Zhang, Emily Feng, Nasrin Saeedian, Gillian I. Bell, Fatemeh Vahedi, David A. Hess, Stephen D. Barr, Ryan M. Troyer, Chil-Yong Kang, Ali A. Ashkar, Matthew S. Miller, S. M. Mansour Haeryfar

**Affiliations:** 1 Department of Microbiology and Immunology, Western University, London, Ontario, Canada; 2 McMaster Immunology Research Centre, McMaster University, Hamilton, Ontario, Canada; 3 Krembil Centre for Stem Cell Biology, Molecular Medicine Research Laboratories, Robarts Research Institute, London, Ontario, Canada; 4 Department of Physiology and Pharmacology, Western University, London, Ontario, Canada; 5 Department of Medicine, McMaster University, Hamilton, Ontario, Canada; 6 Department of Biochemistry and Biomedical Sciences, McMaster University, Hamilton, Ontario, Canada; 7 Michael G. DeGroote Institute for Infectious Disease Research, McMaster University, Hamilton, Ontario, Canada; 8 Division of Clinical Immunology and Allergy, Department of Medicine, Western University, London, Ontario, Canada; 9 Division of General Surgery, Department of Surgery, Western University, London, Ontario, Canada; 10 Lawson Health Research Institute, London, Ontario, Canada; University of Pittsburgh, UNITED STATES

## Abstract

Mucosa-associated invariant T (MAIT) cells are MR1-restricted, innate-like T lymphocytes with tremendous antibacterial and immunomodulatory functions. Additionally, MAIT cells sense and respond to viral infections in an MR1-independent fashion. However, whether they can be directly targeted in immunization strategies against viral pathogens is unclear. We addressed this question in multiple wild-type and genetically altered but clinically relevant mouse strains using several vaccine platforms against influenza viruses, poxviruses and severe acute respiratory syndrome coronavirus 2 (SARS-CoV-2). We demonstrate that 5-(2-oxopropylideneamino)-6-D-ribitylaminouracil (5-OP-RU), a riboflavin-based MR1 ligand of bacterial origin, can synergize with viral vaccines to expand MAIT cells in multiple tissues, reprogram them towards a pro-inflammatory MAIT1 phenotype, license them to bolster virus-specific CD8^+^ T cell responses, and potentiate heterosubtypic anti-influenza protection. Repeated 5-OP-RU administration did not render MAIT cells anergic, thus allowing for its inclusion in prime-boost immunization protocols. Mechanistically, tissue MAIT cell accumulation was due to their robust proliferation, as opposed to altered migratory behavior, and required viral vaccine replication competency and Toll-like receptor 3 and type I interferon receptor signaling. The observed phenomenon was reproducible in female and male mice, and in both young and old animals. It could also be recapitulated in a human cell culture system in which peripheral blood mononuclear cells were exposed to replicating virions and 5-OP-RU. In conclusion, although viruses and virus-based vaccines are devoid of the riboflavin biosynthesis machinery that supplies MR1 ligands, targeting MR1 enhances the efficacy of vaccine-elicited antiviral immunity. We propose 5-OP-RU as a non-classic but potent and versatile vaccine adjuvant against respiratory viruses.

## Author summary

Mucosa-associated invariant T (MAIT) cells are evolutionarily conserved lymphocytes that recognize and respond to bacterial metabolites presented by an antigen-presenting molecule called MR1. Although MAIT cells have been implicated in antiviral immunity, whether their stimulation by MR1 ligands can improve immune responses to viral invaders has been unclear. We addressed this question in several mouse models and primary and prime-boost immunization strategies against influenza viruses, poxviruses and severe acute respiratory syndrome coronavirus 2 (SARS-CoV-2). We demonstrate that an MR1 ligand called 5-(2-oxopropylideneamino)-6-D-ribitylaminouracil (5-OP-RU) can be incorporated into select vaccine formulations to expand tissue MAIT cells and reprogram them towards a pro-inflammatory phenotype, to enhance conventional virus-specific T cell responses, and to promote protection against influenza A viruses (IAVs). We found this phenomenon to be dependent upon the *in vivo* engagement of a pathogen recognition receptor called Toll-like receptor 3 as well as the receptor for type I interferons. Also importantly, MAIT cell proliferation in response to an IAV strain and 5-OP-RU could be phenocopied in a human white blood cell culture system. We propose that 5-OP-RU, and potentially other MR1 ligands, may be used to increase the efficacy of certain vaccines against respiratory viral infections.

## Introduction

Infection- and vaccine-elicited protection against viral invaders relies heavily on primary and anamnestic T and B cell responses, which are controlled and shaped by the innate arm of immunity. While many classic adjuvants prolong and enhance peptide antigen (Ag) presentation by the highly polymorphic major histocompatibility complex (MHC) molecules, targeting innate immune effectors is considered an attractive strategy to bolster antiviral defense mechanisms across genetically diverse populations.

Mucosa-associated invariant T (MAIT) cells are an evolutionarily conserved subset of innate-like T lymphocytes with impressive antimicrobial, cytotoxic and immunomodulatory properties [1,2]. The canonically rearranged invariant T cell receptor (*i*TCR) α chain of MAIT cells (typically Vα19-Jα33 in mice and Vα7.2-Jα33 in humans) is paired with one of only a limited number of available β chains [3–6] to enable recognition of riboflavin derivatives of bacterial origin displayed by a monomorphic MHC class Ib molecule called MHC-related protein 1 (MR1) [7–9]. Such derivatives are best exemplified by 5-(2-oxopropylideneamino)-6-D-ribitylaminouracil (5-OP-RU).

Viruses are devoid of the riboflavin synthesis machinery and consequently unable to activate MAIT cells in an MR1/*i*TCR-dependent manner. However, many viral infections lead to MAIT cell activation, primarily driven by inflammatory cytokines such as interleukin (IL)-7, -12, -15, -18 and type I interferons (TI-IFNs) [10–13]. The importance of MAIT cells in antiviral immune surveillance is evidenced by the heightened morbidity and mortality of influenza A virus (IAV)-infected *Mr1*^-/-^ mice that lack MAIT cells [14]. In addition, a history of bacterial pneumonia after infection with varicella-zoster virus and persistent tattoo-associated human papilloma virus-positive warts was recently reported in an individual carrying a homozygous point mutation in *MR1* [15]. Finally, certain viruses impair MR1 expression or destroy MAIT cells as immune evasion tactics whose *in vivo* relevance remains to be better understood [16–19].

MAIT cells should be regarded as emergency responders to viral infections and their consequences and also as potentially pivotal players in vaccine-induced immunity. First, they are abundant in peripheral blood and enriched in mucosal tissues that provide entry points, transport systems or propagation sites for many viruses or vaccines. For instance, MAIT cells can comprise >2% of all T cells in the human lung where they safeguard against respiratory pathogens [20], including but not limited to IAVs and severe acute respiratory syndrome coronavirus 2 (SARS-CoV-2) [21,22]. Second, MAIT cells have a memory-like phenotype and poised effector functions, including rapid cytokine production [23,24]. Once activated, they release T helper (T_H_)-1-, T_H_2- and/or T_H_17-type cytokines that transregulate the biological behaviors of a myriad of cell types, including dendritic cells (DCs) [25], natural killer (NK) cells [25], CD4^+^ and CD8^+^ conventional T (T_conv_) cells [26,27], and B cells [28–30], all of which participate in antiviral defense. Third, MAIT cells constitutively express or acquire cytolytic effector molecules, including perforin (PFN), granzymes (GZMs) and granulysin, during viral infections, which can be deployed to eliminate MR1^+^ target cells and extracellular bacteria encountered during secondary infections [2,31,32]. Furthermore, certain viral diseases and the cytokines they evoke (*e*.*g*., hepatitis A and IL-15) upregulate natural killer group 2, member D (NKG2D) on MAIT cells, thus potentiating MR1-independent MAIT cell-mediated cytotoxicity against stressed or damaged target cells [33].

The inherent immunomodulatory capacity of MAIT cells has been demonstrated in the contexts of IAV infection and adenovirus-based immunization for coronavirus disease 2019 (COVID-19) [14,27]. However, whether MR1 ligands can be employed as vaccine adjuvants for viral diseases is not clearly understood. In this study, we used wild-type (WT) and MAIT cell-sufficient mouse strains, multiple vaccination models and routes, and a human cell culture system to demonstrate that 5-OP-RU can indeed serve as an efficacious adjuvant to enlarge tissue MAIT cell compartments, to generate MAIT cells with pro-inflammatory and immunoprotective properties, and to augment virus-specific T_conv_ cell responses.

## Materials and Methods

### Ethics statement

Animal experiments were performed following the Canadian Council on Animal Care guidelines and using animal use protocols (AUPs) approved by Animal Care and Veterinary Services at Western University (AUPs 2018–093, 2019–054 and 2020–084) or Animal Research Ethics Board at McMaster University (AUP 19-12-32).

Human peripheral blood was collected from healthy donors who provided formal written consent under protocol #5545, which was approved by the Western University Research Ethics Board for Health Sciences Research Involving Human Subjects.

### Mice

Adult WT C57BL/6 (B6), BALB/c and C3H/HeN mice were purchased from Charles River Canada (St. Constant, QC) and used at ~6–14 weeks of age. Old WT B6 mice, which were provided by Dr. Tony Rupar (Western University), were housed in a pathogen-free barrier facility at Western University until they were used at 18–22 months of age. B6-MAIT^CAST^ mice and *Mr1*^-/-^ mice on the same background [34] were provided by Dr. Olivier Lantz (Institut Curie, Paris, France) and bred in-house. Using sex- and age-matched animals was ensured in this study as appropriate.

### Viruses, vaccines and immunization routes

Several IAV strains, namely A/Puerto Rico/8/1934 (PR8) (H1N1), A/Northern Territory/60/1968 (NT60) (H3N2), A/Hong Kong/1/1968 (HK) (H3N2), and the X31 reassortant (H3N2), were propagated in embryonated chicken eggs before infectious allantoic fluid was harvested, filter-sterilized and stored at -80°C until use. X31 is a chimeric virus expressing the PR8 internal genes along with the hemagglutinin and neuraminidase of an H3N2 virus strain [35]. Mice received an intraperitoneal (*i*.*p*.) inoculum approximating 600 hemagglutinating units of the above-indicated viruses. In several experiments, PR8 was inactivated at 56°C for 30 minutes before injection.

To study IAV-specific recall responses, we used a prime-boost immunization protocol in which B6-MAIT^CAST^ or WT B6 mice were injected *i*.*p*. with PR8 followed, 4 or 6 weeks later, by an *i*.*p*. injection of X31 as we previously described [36].

In a few experiments, BALB/c mice were inoculated intranasally (*i*.*n*.) with 20 μL of the 2021–2022 quadrivalent FluMist spray (AstraZeneca Canada Inc.), 10 μL in each nare, under light isoflurane-induced anesthesia. This vaccine contains live attenuated reassortants from A/Victoria/1/2020 (H1N1) [A/Victoria/2570/2019 (H1N1)pdm09-like virus], A/Tasmania/503/2020 (H3N2) [A/Cambodia/e0826360/2020 (H3N2)-like virus], B/Phuket/3073/2013 (Yamagata lineage), and B/Washington/02/2019 (Victoria lineage).

Separate cohorts of BALB/c mice were injected intramuscularly (*i*.*m*.) in the right hind limb with 5 × 10^8^ plaque-forming units (PFUs) of recombinant vesicular stomatitis viruses (rVSVs) expressing the Spike protein of the Wuhan strain of SARS-CoV-2 [37]. Animals received either a single dose of the Indiana strain (rVSV_Ind_) or two-dose immunization with rVSV_Ind_ and the New Jersey strain (rVSV_NJ_) given 24 days apart.

To model antipoxviral immunization, B6 mice were injected *i*.*p*. with 10^6^ PFUs of the Western Reserve strain of vaccinia virus (VacV), which was propagated using the thymidine kinase-deficient osteosarcoma cell line 143B.

### *In vivo* treatments

To stimulate MAIT cells with an MR1 ligand, 5-OP-RU was administered *i*.*p*., *i*.*m*., or *i*.*n*. at estimated quantities of ~10 nanomoles, ~1 nanomole, or ~76 picomoles per dose, respectively [38,39]. To generate 5-OP-RU, 5-amino-6-D-ribitylaminouracil, which was generously provided by Dr. Olivier Lantz (Institut Curie), and methylglyoxal (Sigma) were mixed at equimolar concentrations in dimethyl sulfoxide (DMSO). After overnight incubation at room temperature, the mixture was stored at -80°C until use, at which point it was thawed and further diluted in sterile phosphate-buffered saline (PBS) for injection. Where indicated, parallel animal cohorts received the same amounts of methylglyoxal and DMSO in PBS as vehicle control.

To prevent tissue trafficking by immune cells, each mouse was injected twice *i*.*p*. with 1 mg/kg of the sphingosine-1-phosphate receptor 1 (S1PR1) antagonist FTY720 (Sigma) 2 hours before and 22 hours after a combination of PR8 and 5-OP-RU was administered [40,41]. FTY720 was reconstituted in water and diluted in PBS before injection, and control animals received PBS only.

In a few experiments, mice were injected *i*.*p*. with 50 μg of the Toll-like receptor (TLR)3 agonist polyinosinic-polycytidylic acid [poly (I:C)] (InvivoGen), 50 μg of the TLR7 agonist imiquimod (InvivoGen), 10^4^ units of recombinant mouse IFN-α1 (rmIFNα1) (R&D Systems), 1 μg of recombinant mouse IFN-β (rmIFNβ) (R&D Systems), or a combination of rmIFNα1 and rmIFNβ, all prepared in PBS.

To sufficiently block IFN α/β receptor subunit 1 (IFNAR-1) *in vivo*, multiple *i*.*p*. injections of an anti-IFNAR-1 monoclonal antibody (mAb) (clone MAR1-5A3) (Bio X Cell, Lebanon, NH) were given. Accordingly, each mouse received 0.5 mg of this mAb 1 hour and 24 hours before immunization with PR8 and 5-OP-RU. This was followed by two additional doses, 0.25 mg each, of MAR1-5A3 at 24 and 48 hours post-immunization. Control cohorts received a mouse IgG1κ isotype control (clone MOPC-21) from Bio X Cell.

### Viral challenge

B6 mice were injected *i*.*p*. with 5-OP-RU, vehicle, the HK virus, or a combination of HK and 5-OP-RU. After 3 days, under isoflurane anaesthesia, mice were challenged *i*.*n*. with 3 × 10^3^ PFUs of the mouse-adapted PR8 virus in 40 μL of saline. Animals were closely monitored for weight loss and mortality in the following 14 days.

### Tissue and specimen processing

At indicated endpoints, mice were euthanized by cervical dislocation. Animals were terminally bled by cardiac puncture using manually heparinized syringes with 26G needles. Non-terminal mouse bleeding was conducted via saphenous venipuncture. Isolated serum samples were frozen at -80°C until they were used for bead-based cytokine multiplexing by Eve Technologies (Calgary, AB) or for antibody titration using enzyme-linked immunosorbent assays (ELISA).

Peritoneal lavage was performed using 10-mL syringes with 18G needles and sterile PBS to obtain peritoneal fluid.

Spleens and livers were harvested aseptically before they were mechanically homogenized. Lungs were minced into small pieces, which were then subjected to enzymatic digestion for 1 hour at 37°C in RPMI-1640 containing 0.5 mg/mL of collagenase type IV (Sigma) and 25 μg/mL of DNase I (Sigma). Hepatic parenchymal cells were removed by density gradient centrifugation at 700 × *g* in 33.75% Percoll PLUS (GE Healthcare). The resulting splenic, pulmonary and hepatic cell preparations were washed, filtered, exposed to ACK (Ammonium-Chlorine-Potassium) Lysing Buffer to eliminate erythrocytes, washed and filtered again. Trypan blue dye exclusion was used to ensure high cellular viability.

To obtain human peripheral blood mononuclear cells (PBMCs), uncoagulated whole blood from male and female donors (age range: 21–66) was spun at 1,200 × *g* in 50-mL SepMate tubes (STEMCELL Technologies) containing Ficoll-Paque PLUS (Cytiva, Uppsala, Sweden).

### Quantitative polymerase chain reaction (PCR) analyses

To isolate pulmonary mouse MAIT cells, non-parenchymal lung mononuclear cells (LMNCs) from indicated B6-MAIT^CAST^ cohorts were subjected to magnetic cell sorting. Briefly, phycoerythrin (PE)-conjugated, 5-OP-RU-loaded mouse MR1 tetramers were used to stain MAIT cells [7,42], which were then purified using Anti-PE MicroBeads UltraPure, LS Columns and a QuadroMACS Separator (Miltenyi Biotec). Isolated MAIT cells were always 90–99% pure after two separation rounds (S1 Fig). Cells were washed in PBS containing 10% BSA and 50 mM ethylenediaminetetraacetic acid before pellets were flash-frozen and stored at -80°C.

Total RNA was extracted using a PicoPure RNA Isolation Kit (Thermo Scientific) and converted to cDNA using SuperScript IV VILO Master Mix with ezDNase (Thermo Scientific). cDNA and TaqMan Fast Advanced Master Mix were added to each well of a custom-made, 96-well TaqMan Array Fast Plate (Thermo Scientific) containing lyophilized primer/probe sets listed in S1 Table. cDNA was amplified per manufacturer’s instructions, and cycle threshold (Ct) values were generated using a StepOne Plus Real-Time PCR System (Applied Biosystems). Normalized ΔCt values were determined by subtracting each Ct value by that of *Gapdh*, and the following formula was employed to calculate the relative mRNA content of MAIT cells for indicated genes: Fold Change = 2^-(ΔΔCt)^.

### *In vivo* MAIT cell proliferation assay

To investigate the proliferative capacity of mouse MAIT cells *in vivo*, we isolated naïve B6-MAIT^CAST^ LMNCs and non-parenchymal hepatic mononuclear cells (HMNCs), which were immediately pooled and subjected to magnetic MAIT cell sorting. Isolated MAIT cells were then expanded *ex vivo* following a recently published protocol by Parrot *et al*. [43]. MAIT cell-depleted fraction was irradiated at 35 Gy and used as a source of feeder cells, which were seeded at 2 × 10^5^ cells/well along with 2 × 10^4^ MAIT cells/well of a U-bottom plate. Cells were maintained in ImmunoCult T Cell Expansion Medium (STEMCELL Technologies) supplemented with 20 ng/mL of recombinant mouse interleukin (IL)-2 (R&D systems), 8% CTS Immune Cell Serum Replacement (Thermo Fisher), 100 μg/mL of Normocin (InvivoGen), and 100 U/mL Penicillin/Streptomycin. Cultures were split twice a week and replenished with fresh medium and feeder cells. After 3 weeks, expanded MAIT cells were washed, labeled with 1 μM CellTrace Far Red dye (Thermo Fisher), and injected *i*.*v*. at 5 × 10^5^ cells/B6-MAIT^CAST^ mouse. Twenty-four hours after adoptive transfer, the recipients were given PBS or immunized with PR8 and 5-OP-RU *i*.*p*. Three days later, mice were sacrificed for several organs in which MAIT cell proliferation was assessed by flow cytometry.

### *Ex vivo* cell stimulation protocols

Mouse splenocytes, peritoneal cells, LMNCs and/or HMNCs were resuspended in RPMI-1640 supplemented with 10% heat-inactivated fetal bovine serum (FBS), 2 mM GlutaMAX-I, 0.1 mM MEM nonessential amino acids, 1 mM sodium pyruvate, 10 mM HEPES, 100 U/mL penicillin and 100 μg/mL streptomycin, which is referred to as complete medium in this work.

Up to 2 × 10^6^ MNCs were seeded into each well of U-bottom plates. Cells were left untreated in complete medium, stimulated for 4 hours with 50 ng/mL of phorbol 12-myristate 13-acetate (PMA) plus 500 ng/mL of ionomycin, or stimulated for 24 hours with 5 ng/mL of recombinant mouse IL-12p70 (Peprotech) plus 5 ng/mL of recombinant mouse IL-18 (R&D Systems). To retain intracellular mediators for downstream cytofluorimetric analyses, 10 μg/mL of brefeldin A (Sigma) was added at the beginning of cultures containing PMA and ionomycin, or after 18 hours of stimulation with IL-12 and IL-18. Cultures were incubated inside a humidified incubator set at 37°C and 5% CO_2_.

In several experiments, MAIT cells were magnetically purified before stimulation. They were rested for 2 hours at 37°C before they were washed, seeded at ~120,000 cells/well, and stimulated with PMA and ionomycin or with IL-12 and IL-18 as described above. In other experiments, MAIT cells were stimulated with plate-coated anti-CD3 (clone 17A2) (Bio X Cell) in the presence of 2 μg/mL of an anti-CD28 mAb (clone 37.51) (Bio X Cell). After 24 hours, culture supernatants were collected, aliquoted and stored at -80°C until their cytokine contents were quantified using Ready-SET-Go! ELISA Kits from eBioscience.

Human PBMCs were seeded at 5 × 10^5^ cells/well in U-bottom microplates in the absence or presence of C1R-MR1 cells that had been left unmanipulated, infected with PR8 and/or pulsed with 2 nM 5-OP-RU. The MR1-overexpresing lymphoblastoid human B cell line C1R-MR1 was provided by Dr. Jose Villadangos (University of Melbourne). To infect C1R-MR1 cells, 10^5^ TCID_50_ (50% tissue culture infectious dose) of PR8 was added to 10^7^ cells/mL in Opti-MEM I Reduced Serum Medium (Thermo Fisher). After 1 hour at 37°C, infected cells were washed, resuspended in complete medium, and co-incubated at a 1:2 ratio with PBMCs for 16, 48 or 96 hours prior to cytofluorimetric analyses of indicated T cell surface markers.

In additional experiments, 10^7^ PBMCs/mL were incubated with 5 μM carboxyfluorescein diacetate succinimidyl ester (CFDA-SE) (Invitrogen) for 20 minutes at 37°C. Labeled cells were then washed, resuspended in complete medium and seeded at 10^6^ cells/well in U-bottom plates either alone or in the presence of unmanipulated, PR8-infected, 5-OP-RU-pulsed, or PR8-infected and 5-OP-RU-pulsed C1R-MR1 cells. Seven days later, CFDA-SE dye dilution was assessed by flow cytometry as a measure of cellular proliferation.

### Flow cytometry

Mouse cells were incubated for 15 minutes on ice with 2.4G2 B cell hybridoma culture supernatant containing a CD16/CD32-blocking mAb. This was to prevent false positive staining due to non-specific binding of fluorochrome-conjugated mAbs to Fcγ receptors.

5-OP-RU-loaded MR1 tetramers and PBS-57-loaded CD1d tetramers were provided by the NIH Tetramer Core Facility (Atlanta, GA) for MAIT and invariant natural killer T (*i*NKT) cell staining, respectively. As negative staining controls, 6-formylpterin (6-FP)-loaded MR1 tetramers and empty CD1d tetramers, also supplied by the NIH Tetramer Core Facility, were utilized. B220^+^ events were excluded to reduce background noise during mouse MAIT cell staining.

Fluorochrome-conjugated mAbs against various cell surface markers and corresponding isotype controls are listed in S2 Table. These reagents were prepared in a staining buffer containing 2% FBS in PBS and added to cell preparations for 20 minutes at room temperature. To detect intracellular mediators, we used the Foxp3/Transcription Factor Staining Buffer Set from Thermo Fisher. After fixation and permeabilization for 20 minutes at room temperature, samples were incubated with indicated mAbs or isotype controls in a permeabilization buffer for 30 minutes at room temperature in the dark. Staining with 7-aminoactinomycin D (7-AAD) or Fixable Viability Dye (eBioscience) allowed for dead cell exclusion in our analyses.

To detect IAV-specific CD8^+^ T cells on day 8 post-FluMist instillation, ~10^6^ LMNCs and 100 μL of whole blood from vaccinated BALB/c mice were stained with an anti-CD8α mAb (clone 53–6.7) and Alexa 488-labeled H-2K^d^:TYQRTRALV tetramers (NIH Tetramer Core Facility). TYQRTRALV is a linear peptide corresponding to the immunodominant peptide epitope of the IAV nucleoprotein (NP), namely NP_147-155_, in the BALB/c strain [44]. Erythrocytes were removed from the whole blood specimen after cell surface staining.

NP_147-155_-specific T cells were also enumerated using intracellular cytokine staining (ICS) for IFN-γ as we previously described [45]. In brief, 2 × 10^6^ LMNCs from vaccinated mice were left untreated, exposed to 500 nM of an H-2^d^-restricted irrelevant peptide derived from the lymphocytic choriomeningitis virus (LCMV) NP (NP_112-126_: RPQASGVYM) [46], or stimulated with 500 nM of TYQRTRALV for 5 hours at 37°C. Brefeldin A was present at 10 μg/mL during the final 3 hours, after which cells were washed, stained with anti-CD8α, fixed and permeabilized, incubated with an anti-IFN-γ mAb (clone XMG1.2), washed again and analyzed.

A similar ICS protocol was used to detect splenic and pulmonary CD8^+^ T cells recognizing an immunodominant epitope of the SARS-CoV-2 Spike protein, namely S_535-543_ (KNKCVNFNF) [47], in vaccinated BALB/c mice.

Stained cells were interrogated using a BD FACSCanto II flow cytometer, and data were analyzed using FlowJo software version 10.0.7 (Tree Star).

### IAV-specific Ab detection assays

*Mr1*^+/+^ and *Mr1*^-/-^ B6-MAIT^CAST^ mice were injected *i*.*p*. with PR8 (± 5-OP-RU) as indicated. Ten and 21 days later, mice were bled, and coagulated whole blood was centrifuged at 17,000 × *g* for 40 minutes at 4°C before serum samples were collected, aliquoted and stored at -80°C.

PR8-infected Madin-Darby Canine Kidney (MDCK) cell lysate was prepared as previously described [48]. Briefly, ~2,500 hemagglutinating units of PR8 were added onto an MDCK monolayer at ~80% confluence inside a T75 flask containing Dulbecco’s Modified Eagle Medium, 100 U/mL Penicillin/Streptomycin and 2 μg/mL N-tosyl-L-phenylalanine chloromethyl ketone-treated trypsin (Sigma). Cells were harvested 36 hours later, washed with PBS, and treated with 1 mM phenylmethylsulfonyl fluoride reconstituted in isopropanol. After three freeze-thaw cycles and centrifugation at 700 × *g* for 15 minutes, a clear cell lysate preparation was obtained, aliquoted and stored at -80°C.

Corning flat-bottomed polystyrene microplates with a high binding surface (Sigma) were coated with a 1:200 dilution of the above lysate in PBS and incubated overnight at 4°C. To block the plates, PBS containing 2% FBS was used for 1 hour at 37°C. After washing the plates with an ELISA wash buffer (0.05% Tween 20 in PBS), serially diluted mouse sera were added, and plates were incubated for 2 hours at room temperature. Plates were then washed to remove unbound sera before they received a horseradish peroxidase-conjugated goat anti-mouse IgM or IgG (Novus Biologicals). After 1 hour at room temperature, plates were washed again and received 100 μL/well of a tetramethylbenzidine substrate solution (Thermo Scientific). The enzymatic reaction was stopped after ~15 minutes by adding 50 μL/well of 1 M phosphoric acid, and optical density (OD) values at 450 nm and 570 nm were measured using a BioTek Cytation 5 Cell Imaging Multimode Reader. OD_570_ readings were subtracted from OD_450_ values to correct for optical imperfections.

### Statistical analyses

Statistical comparisons were carried out using GraphPad Prism 8.0.1 software. Chi-squared tests, Student’s *t*-test, or ANOVA with relevant post-hoc tests were employed as appropriate, and differences with *p* ≤ 0.05 were considered significant. Sample sizes and the statistical methods used are detailed within figure legends.

## Results

### 5-OP-RU synergizes with a PR8-based vaccine to induce robust MAIT cell accumulation in multiple tissues

MAIT cells can be activated by viral infections or in response to anti-pathogen vaccines [22,27]. However, whether MR1 ligands, typified by 5-OP-RU, optimize the efficacy of antiviral immunization strategies has been unclear. We began to address this question in a mouse model in which PR8 is inoculated *i*.*p*. This well-established model simulates seasonal flu vaccination protocols through an unnatural route, resulting in robust local and systemic immune responses in the absence of severe complications and morbidity [36,45,48–52].

Unlike in humans, MAIT cells are scarce in conventional strains of laboratory mice. Therefore, we used a congenic strain, namely B6-MAIT^CAST^, which harbors ~20 times more MAIT cells compared with WT B6 mice [34] and, as such, phenocopy human MAIT cell repertoires.

**Fig 1:**
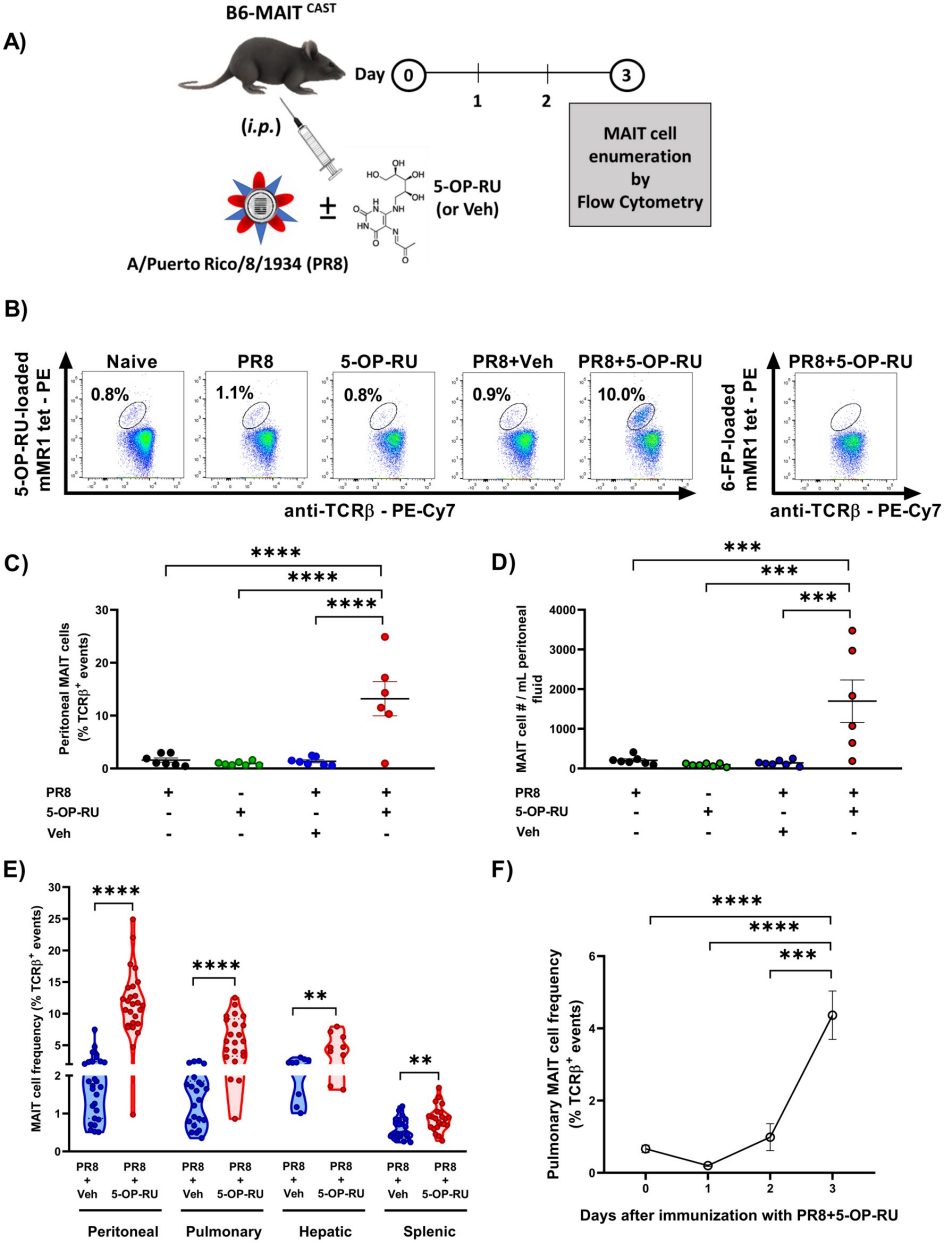
Intraperitoneal co-administration of PR8 and 5-OP-RU results in MAIT cell accumulation in multiple tissues. B6-MAIT^CAST^ mice were left unimmunized or inoculated *i*.*p*. with PR8, 5-OP-RU/vehicle, or both followed, 3 days later (**A-E**) or at indicated time points (**F**), by cytofluorimetric enumeration of MAIT cells using 5-OP-RU-loaded mouse MR1 tetramers in the peritoneal cavity (**B-E**), lungs (**E-F**), liver and spleen (**E**). 6-FP-loaded mouse MR1 tetramers were used as a staining control (**B**). Peritoneal MAIT cell frequencies (**B-C**) and their absolute numbers (**D**) are illustrated. Each circle (**C-E**) represents an individual mouse. For the kinetics of the pulmonary MAIT cell response to PR8 plus 5-OP-RU, data from day 0 (n = 5), day 1 (n = 3), day 2 (n = 5) and day 3 (n = 5) were analyzed, with error bars representing the standard errors of the mean (SEM) (**F**). Data shown in panels **C**, **D**, **E** and **F** are from 2, 2, 15 and 2 independent experiments, respectively. One-way ANOVA was followed by Dunnett’s post-hoc multiple comparisons (**C, D and F**). For group comparisons, Mann-Whitney *U* test and unpaired *t*-tests were employed as appropriate (**E**). **, *** and **** denote significant differences with *p* ≤ 0.01, *p* ≤ 0.001 and *p* ≤ 0.0001, respectively.

MAIT cells comprised only a tiny fraction of peritoneal TCRβ^+^ cells in naïve B6-MAIT^CAST^ mice or in animals that had received either PR8 or 5-OP-RU alone 3 days earlier (Fig 1A–1D). However, combining PR8 and 5-OP-RU resulted in massive MAIT cell accumulation inside the peritoneal cavity (Fig 1B–1D). This was not a transient response since both the frequency and the absolute number of peritoneal MAIT cells remained high on day 7 (S2 Fig).

Importantly, *i*.*p*. inoculation of 5-OP-RU plus PR8 enlarged the MAIT cell compartment not only in the peritoneal cavity but also in the lungs, liver and spleen (Fig 1E). The pulmonary response kinetics pointed to early MAIT cell activation since these cells were less detectable by MR1 tetramer staining on day 1 (Fig 1F), due likely to activation-induced *i*TCR internalization.

We confirmed the specificity of 5-OP-RU, as an adjuvant candidate, for MAIT cells by enumerating other T cell types, including *i*NKT cells, a prominent invariant T cell type in rodents. As expected, pulmonary, hepatic and splenic *i*NKT cell frequencies remained unaltered post-vaccination (S3 Fig).

### Incorporating 5-OP-RU in secondary IAV immunization leads to increased MAIT cell numbers

**Fig 2:**
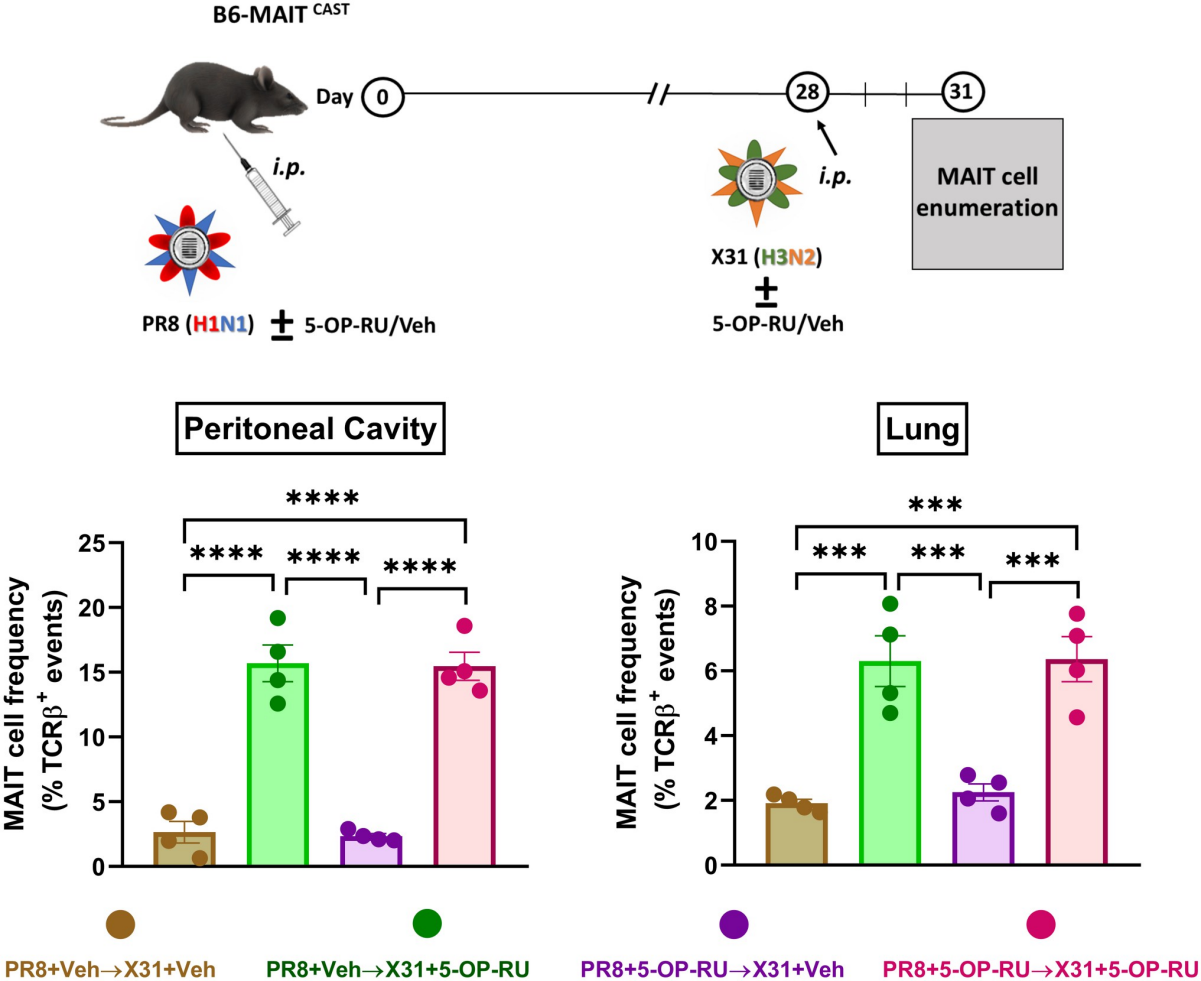
5-OP-RU synergizes with IAV in a prime-boost immunization model. In two independent experiments, B6-MAIT^CAST^ mice (n = 4/cohort) were primed *i*.*p*. with PR8 (H1N1) plus 5-OP-RU/vehicle four weeks before they received a boosting injection of the reassortant X31 virus (H3N2) with 5-OP-RU/vehicle. Three days later, peritoneal and pulmonary MAIT cell frequencies were determined by flow cytometry. Each circle corresponds to an individual mouse, and error bars represent SEM values. Statistical analyses were performed using one-way ANOVA followed by Tukey’s Multiple Comparisons tests. *** and **** denotes significant differences with *p* ≤ 0.001 and *p* ≤ 0.0001, respectively.

To test the efficacy of 5-OP-RU in a heterosubtypic prime-boost immunization model mimicking annual IAV booster vaccinations, we inoculated B6-MAIT^CAST^ mice *i*.*p*. with PR8 (H1N1) followed, 4 weeks later, by an *i*.*p*. injection of the reassortant H3N2 virus X31 [35,36]. This strategy prevents the Abs generated through PR8 priming from quickly removing the boosting X31 virions from the system. As with the primary response, the enhancing effect of 5-OP-RU was evident when this compound was added to X31 (Fig 2). Pulmonary MAIT cell numbers were also elevated 3 days after immunization with X31 and 5-OP-RU when WT B6 mice were used in lieu of B6-MAIT^CAST^ mice (S4 Fig).

We next asked whether including 5-OP-RU in both primary and secondary immunizations further increases MAIT cell frequencies. This was clearly not the case since peritoneal and pulmonary MAIT cell percentages were similar in animals receiving 5-OP-RU during both phases and those receiving 5-OP-RU in the boosting phase only (Fig 2). A similar pattern emerged when hepatic and splenic MAIT cells were enumerated (S5A and S5B Fig). This experiment also demonstrates that a prior exposure to 5-OP-RU does not prevent MAIT cell expansion in a subsequent response to the same molecule.

### 5-OP-RU-induced MAIT cell accumulation is observed in both sexes, in young and old mice, and across multiple genetic strains and vaccines

**Fig 3:**
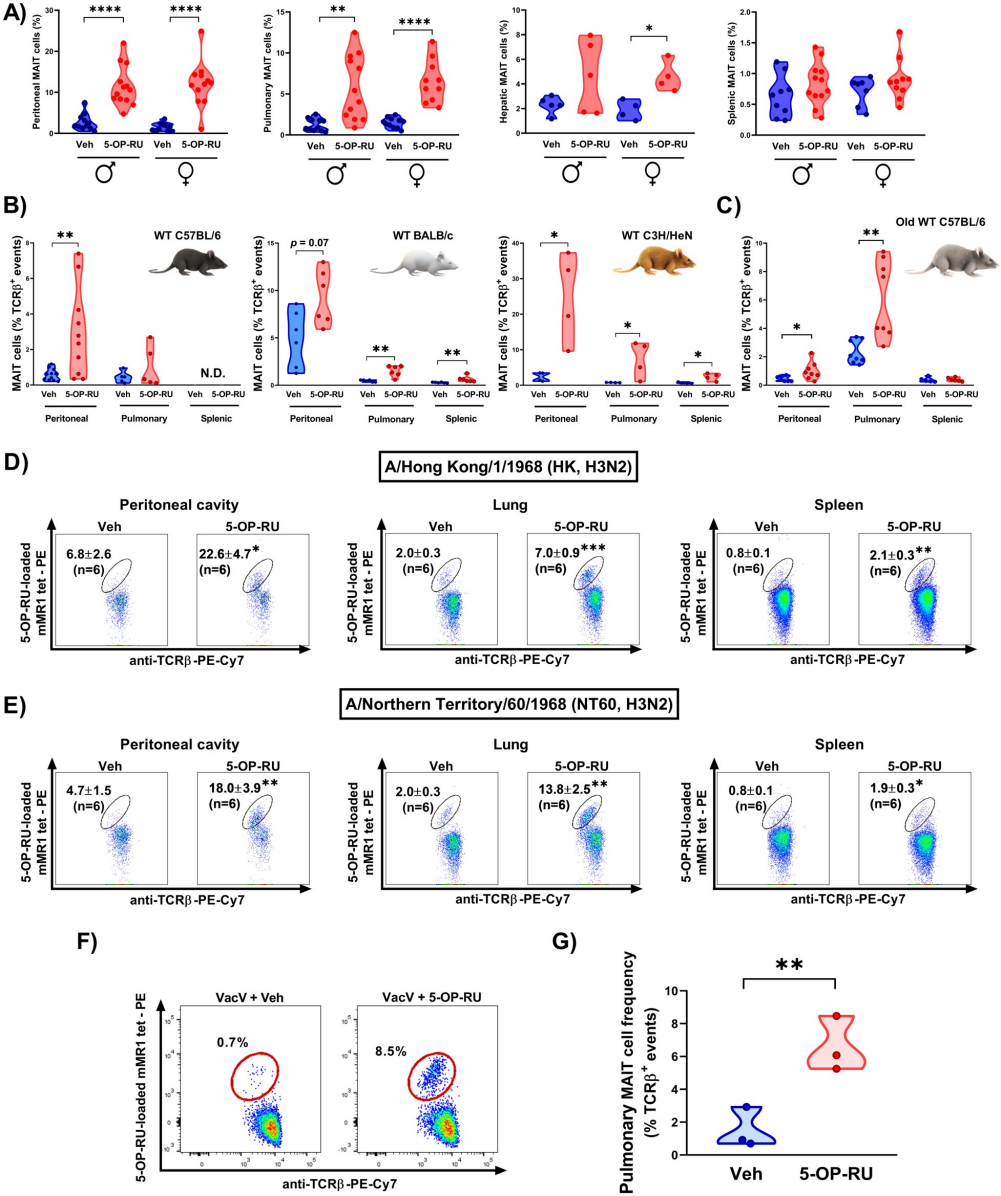
The adjuvanticity of 5-OP-RU manifests itself in both sexes, in young and old animals, and in different mouse strains receiving immunization with various IAV strains or VacV. Age-matched male (♂) and female (♀) B6-MAIT^CAST^ mice (**A**), sex-matched young (6-14-week-old) and old (18-22-month-old) wild-type C57BL/6 (H-2^b^) mice (**B-C**), and age/sex-matched wild-type BALB/c (H-2^d^) and C3H/HeN (H-2^k^) mice (**B**) were inoculated *i*.*p*. with PR8 and 5-OP-RU (or vehicle). Separate cohorts of B6-MAIT^CAST^ mice were immunized *i*.*p*. with the H3N2 IAV strain HK (**D**) or NT60 (**E**) or with vaccinia virus (VacV) (**F-G**) in combination with either 5-OP-RU or vehicle as indicated. MAIT cell frequencies in specified sites were determined by flow cytometry on day 3 post-immunization. Each circle corresponds to an individual mouse in violin plots (**A-C and G**). Representative plots are also depicted (**D-F)** with mean ± SEM values indicated (**D-E**). Data shown in panels **B-E** summarize experiments in which equal or close to equal numbers of female and male mice were used. Panel **G** illustrates data from 3 male mice/group. Data shown in panel **A** are from 11, 10, 3 and 11 experiments in which peritoneal, pulmonary, hepatic and splenic MAIT cells were enumerated, respectively. Data shown in panel **B** are from 5, 2 and 2 experiments in which C57BL/6, BALB/c and C3H/HeN mice were used, respectively. Data shown in panels **C**, **D** and **E** are from 3, 2 and 2 experiments, respectively. Unpaired *t*-tests were employed for group comparisons. *, **, *** and **** denote significant differences with *p* ≤ 0.05, *p* ≤ 0.01, *p* ≤ 0.001 and *p* ≤ 0.0001, respectively.

To investigate whether the enhancing effect of 5-OP-RU is present in both sexes, we retrospectively determined MAIT cell frequencies in closely age-matched female and male mice receiving PR8 in combination with either 5-OP-RU or vehicle. These analyses confirmed that both sexes mount similarly rigorous responses to a 5-OP-RU-adjuvanted PR8 vaccine (Fig 3A).

In the next series of experiments, we tested the efficacy of 5-OP-RU in several WT mouse strains, including B6 (H-2^b^), BALB/c (H-2^d^) and C3H/HeN (H-2^k^). Although MAIT cells are rare in these animals, *i*.*p*. co-administration of PR8 and 5-OP-RU made tissue MAIT cells readily detectable and raised their frequencies (Fig 3B).

MAIT cells undergo a numerical decline with age [53,54], and an optimal vaccine should work in both young and old individuals. We found the combination of 5-OP-RU and PR8 to induce a MAIT cell surge not only in 6-14-week-old young animals but also in 18-22-month-old B6 mice (Fig 3C).

Finally, we sought to explore the adjuvanticity of 5-OP-RU when used in conjunction with IAV strains other than the mouse-adapted PR8 strain (H1N1) or even non-IAV vaccines. We found a dramatic rise in MAIT cell numbers following *i*.*p*. immunization with two non-mouse-adapted H3N2 strains, namely HK (Fig 3D) and NT60 (Fig 3E). Also interestingly, this phenomenon was reproducible when we used VacV (Fig 3F and 3G), a DNA virus with a radically different genomic structure and tissue tropism compared with IAVs. Immunization with VacV prevents smallpox and is also thought to elicit cross-protective responses against monkeypox [55].

### *In vivo* proliferation of MAIT cells accounts for their tissue accumulation after 5-OP-RU-adjuvanted anti-IAV vaccination

**Fig 4:**
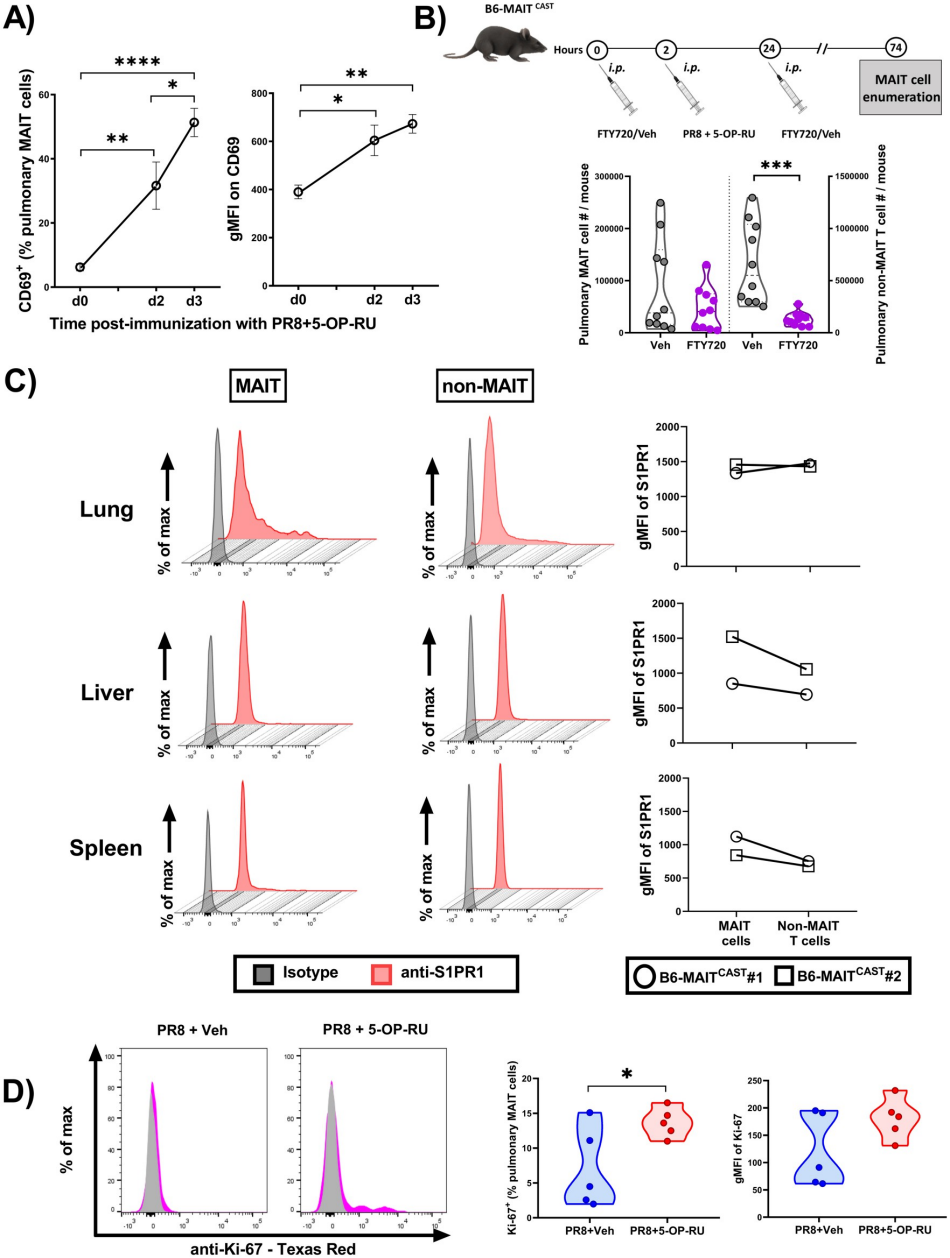
Immunization with 5-OP-RU-adjuvanted PR8 leads to CD69 and Ki-67 upregulation on MAIT cells and their tissue expansion in an S1PR1-independent manner. B6-MAIT^CAST^ mice (n = 5/group in two independent experiments) were injected *i*.*p*. with PR8 plus 5-OP-RU (**A** and **D**) or vehicle (**D**) 3 days before pulmonary MAIT cells were analyzed for CD69^+^ (**A**) and Ki-67^+^ (**D**) cell frequencies and for the geometric mean fluorescence intensity (gMFI) of staining for these markers (**A** and **D**). Separate cohorts (n = 10/group pooled from four independent experiments yielding similar results) received *i*.*p*. injections of FTY720 (or vehicle) 2 hours before and 22 hours after immunization with PR8 and 5-OP-RU, followed by pulmonary TCRβ^+^ MR1 tetramer^+^ MAIT and TCRβ^+^ MR1 tetramer^-^ non-MAIT T cell enumeration on day 3 post-immunization (**B**). The surface expression levels of S1PR1 on tissue MAIT and non-MAIT T cells were also evaluated in naïve B6-MAIT^CAST^ mice (n = 2) (**C**). Representative plots from one mouse and gMFI values for both animals are depicted (**C**). Statistical differences were computed using one-way ANOVA followed by Tukey’s Multiple Comparisons test (**A**), Mann-Whitney *U* test (**B**) and unpaired *t*-test (**D**). *, **, *** and **** denote differences with *p* ≤ 0.05, *p* ≤ 0.01, *p* ≤ 0.001 and *p* ≤ 0.0001, respectively.

To begin to address the observed phenotype mechanistically, we asked whether MAIT cell accumulation was a result of their enhanced proliferative capacity or altered migratory behavior. We took several approaches to answer this question. First, we examined MAIT cells from B6-MAIT^CAST^ mice for their expression of CD69, an early activation marker that mediates tissue retention by suppressing S1PR1 [56,57]. Animals that had received PR8 plus 5-OP-RU showed a gradual rise in both the proportion of CD69^+^ MAIT cells and the geometric mean fluorescence intensity (gMFI) of CD69 staining, indicating the higher expression level of this molecule on a per-cell basis (Fig 4A and S6 Fig). Second, treating mice with FTY720 (*aka*., fingolimod), a drug that antagonizes S1PR1 to block lymphocyte trafficking [40,41], failed to reverse MAIT cell accumulation in the lungs (Fig 4B), liver and peritoneal cavity (S7 Fig). In contrast, non-MAIT T lymphocytes that consist primarily of T_conv_ cells were significantly decreased in the above sites. The noted difference was not due to absent S1PR1 expression by MAIT cells. In fact, S1PR1 expression levels on the surface of MAIT cells were comparable with or higher than those displayed by non-MAIT T cells (Fig 4C). Third, we found vaccination with PR8 plus 5-OP-RU to upregulate Ki-67, a proliferation marker, on MAIT cells (Fig 4D). Finally, to evaluate MAIT cell expansion more definitively, we adoptively transferred magnetically purified, *ex vivo*-expanded, CellTrace-labeled MAIT cells into B6-MAIT^CAST^ mice, which were then injected with PBS or immunized with PR8 plus 5-OP-RU. Three days later, substantial CellTrace dye dilution among pulmonary, hepatic and splenic MAIT cells was detectable in vaccinated animals only, indicative of *in vivo* MAIT cell proliferation (S8 Fig).

Taken together, the above results demonstrate that MAIT cell accumulation following 5-OP-RU-adjuvanted IAV immunization stems from the *in situ* expansion of these cells in indicated tissues as opposed to their recruitment from other locations.

### The adjuvant effect of 5-OP-RU depends on vaccine replication, TLR3 engagement and IFNAR signaling

**Fig 5:**
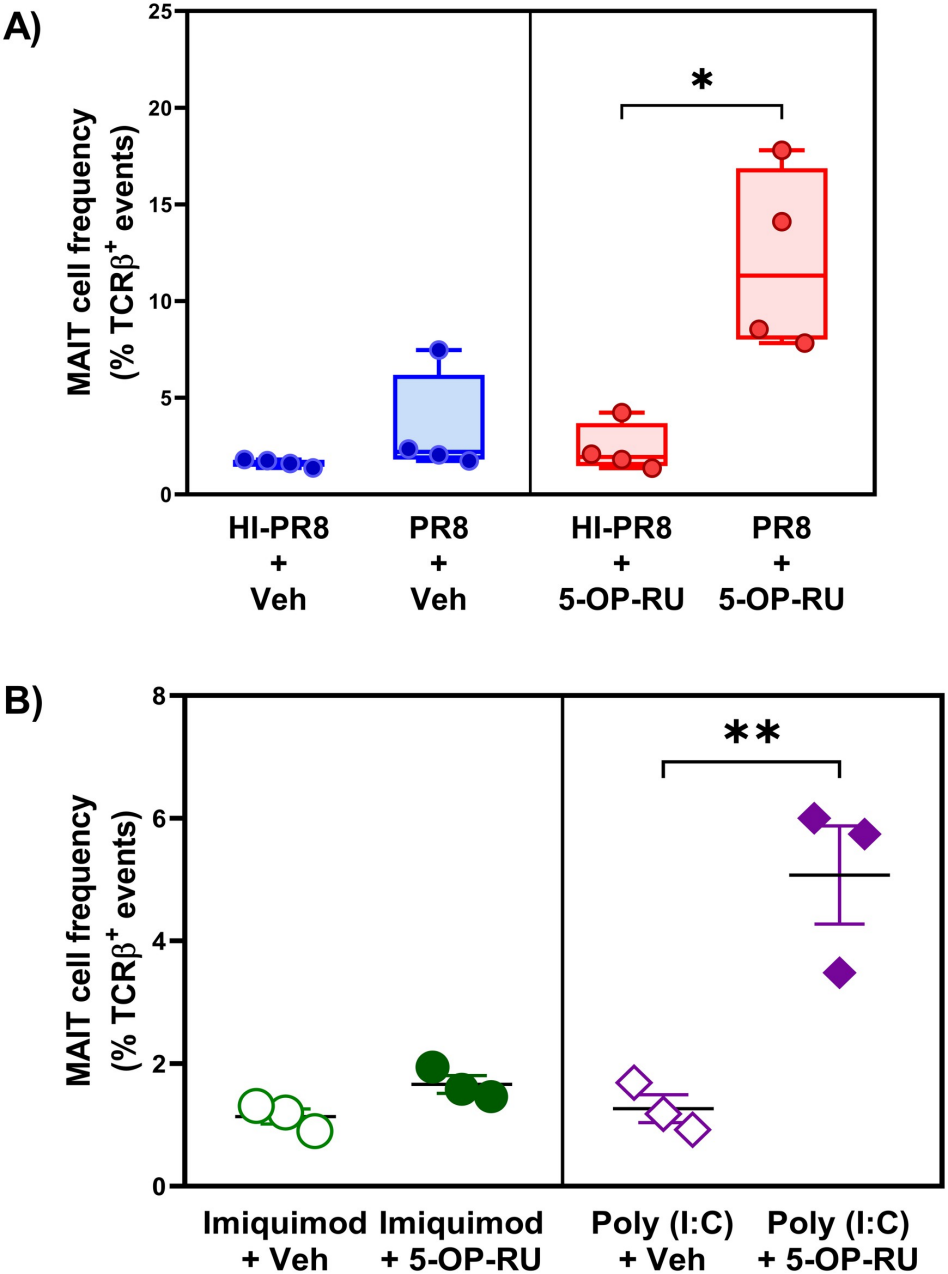
5-OP-RU can be combined with poly (I:C), but not with heat-inactivated PR8, to expand the peritoneal MAIT cell population. B6-MAIT^CAST^ mice (n = 4/group in two independent experiments) were inoculated *i*.*p*. with intact or heat-inactivated PR8 (HI-PR8) in combination with 5-OP-RU or vehicle as indicated (**A**). Additional cohorts (n = 3/group) were injected *i*.*p*. with poly (I:C) or imiquimod plus 5-OP-RU or vehicle (**B**). Three days later, peritoneal MAIT cell frequencies were determined by flow cytometry. Each circle or diamond represents an individual mouse. Statistical comparisons were made using unpaired *t*-tests, with significant differences identified.

To determine whether active viral propagation was required for the adjuvant effect of 5-OP-RU, we compared intact PR8 and heat-inactivated PR8 (HI-PR8). As illustrated in Fig 5A, HI-PR8 failed to synergize with 5-OP-RU to prompt MAIT cells to accumulate in the peritoneal cavity. This finding, coupled with our observation that fundamentally different viruses (*i*.*e*., IAVs and VacV) could be combined with 5-OP-RU to induce MAIT cell expansion (Figs 1 and 3D–3G), suggested a role for double-stranded ribonucleic acid (dsRNA) in our model.

The presence of dsRNA, a by-product of viral replication, can be sensed by TLR3, resulting in a TI-IFN response [58]. We found poly (I:C), a synthetic analog of viral dsRNA and a TLR3 agonist, to expand MAIT cells when co-administered with 5-OP-RU (Fig 5B), thus partially simulating the effect of the PR8 virions (S9 Fig). In contrast, using imiquimod to trigger TLR7, a major IAV recognition receptor [59,60], did not increase the frequency of MAIT cells (Fig 5B and S9 Fig).

**Fig 6:**
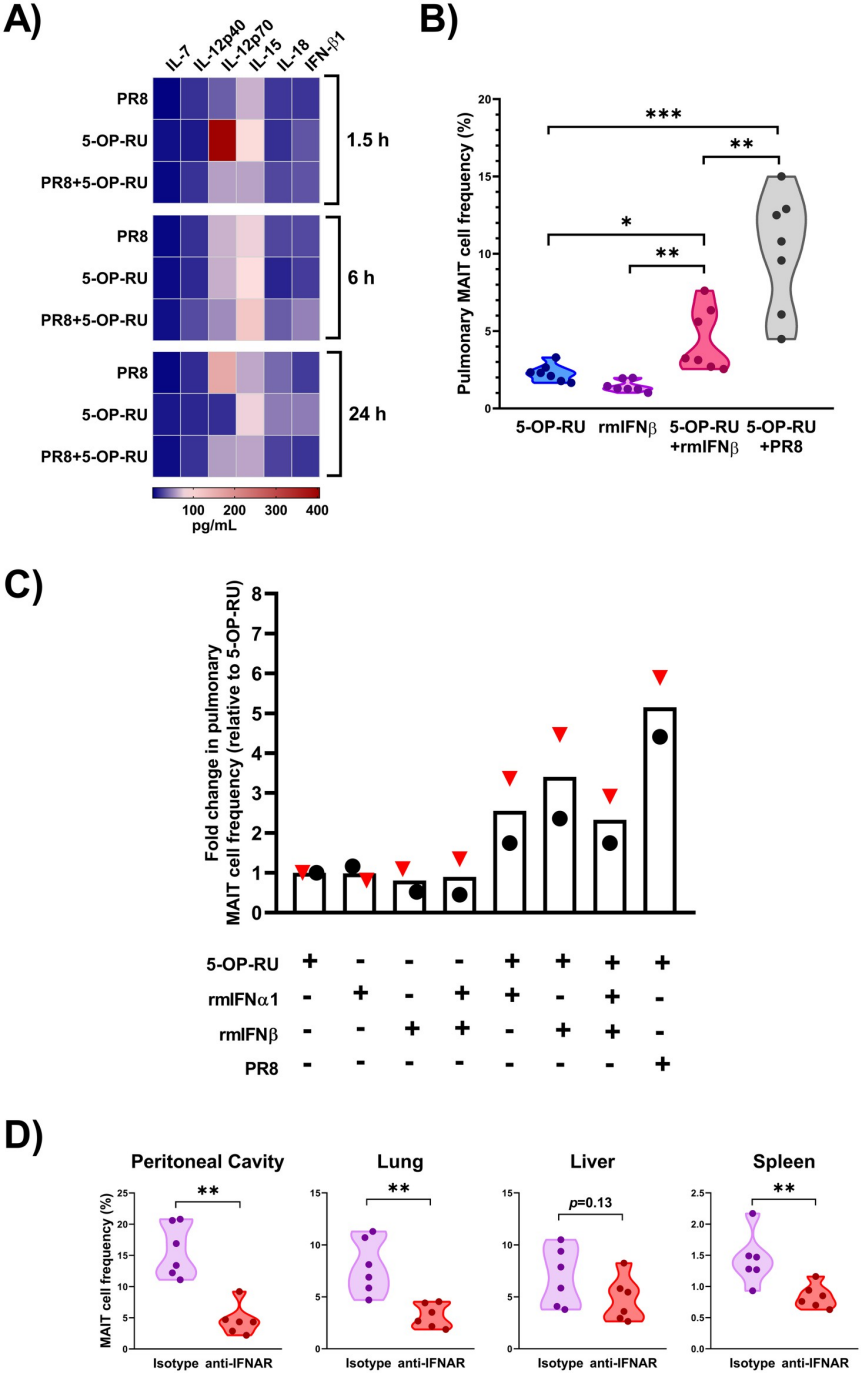
IFNAR signaling is required for tissue MAIT cell accumulation following immunization with PR8 plus 5-OP-RU. (**A**) B6-MAIT^CAST^ mice were injected *i*.*p*. with PR8 (n = 12), 5-OP-RU (n = 12), or both (n = 12). Animals (n = 4/timepoint) were sacrificed 1.5, 6 or 24 hours later, and indicated cytokines were quantified in serum samples. A heatmap was generated to visualize average values at specified timepoints. (**B**) B6-MAIT^CAST^ mice (n = 7/group in four independent experiments) were injected *i*.*p*. with 5-OP-RU, recombinant mouse IFN-β (rmIFNβ), 5-OP-RU plus rmIFNβ, or 5-OP-RU plus PR8. (**C**) In two additional experiments, mice (n = 2/group) received 5-OP-RU, rmIFNα1, rmIFNβ and/or PR8 in indicated combinations. Pulmonary MR1 tetramer^+^ cell percentages among TCRβ^+^ events (**B**) and fold changes in MAIT cell frequencies relative to 5-OP-RU treatment alone (**C**) are shown. Black circles and red triangles each represent an individual mouse used in experiment 1 and experiment 2, respectively. (**D**) Additional cohorts (n = 6/group in two independent experiments) received an IFNAR-blocking monoclonal antibody or isotype control twice before and twice after *i*.*p*. immunization with PR8 and 5-OP-RU as detailed in Materials and Methods. Peritoneal, pulmonary, hepatic and splenic MAIT cells were enumerated by flow cytometry on day 3 post-immunization. Unpaired *t*-tests were employed for statistical comparisons, and *, ** and *** denote differences with *p* ≤ 0.05, *p* ≤ 0.01 and *p* ≤ 0.001, respectively.

Among detectable cytokines in the sera of vaccinated B6-MAIT^CAST^ mice (S10 Fig), we focused on IL-7, IL-12, IL-15, IL-18 and TI-IFNs, which are known to stimulate MAIT cells [11,22]. These analyses revealed a noticeable rise in IFN-β levels in animals receiving PR8 plus 5-OP-RU at the six-hour timepoint (Fig 6A). Given the importance of the TLR3-IFN axis in antiviral responses, we sought to investigate the role of TI-IFNs in our model. First, we injected mice with 5-OP-RU, rmIFNβ, or both before enumerating pulmonary MAIT cells. We found rmIFNβ administration to raise the frequency of MAIT cells only if it was combined with 5-OP-RU (Fig 6B). This was a robust response although it did not reach the same magnitude as that achieved by a combination of 5-OP-RU and PR8 at least on day 3 (Fig 6B).

Our cytokine multiplexing panel (S10 Fig) did not include IFN-α, another TI-IFN that signals through IFNAR-1. Therefore, we tested the efficacy of rmIFNα1, alone or in conjunction with rmIFNβ, in additional experiments. In the absence of 5-OP-RU, these TI-IFNs failed to enlarge the population of pulmonary MAIT cells (Fig 6C). When combined with 5-OP-RU, rmIFNα1 and rmIFNβ were comparable in their ability to expand MAIT cells. However, this effect could not be further augmented by injecting mice with a cocktail of 5-OP-RU, rmIFNα1 and rmIFNβ used at indicated doses (Fig 6C).

Importantly, mAb-mediated blockade of IFNAR-1 prevented tissue MAIT cell accumulation in animals receiving PR8 and 5-OP-RU (Fig 6D). Therefore, IFNAR signaling was strictly required for MAIT cell expansion in response to this vaccination strategy.

### Immunization with PR8 plus 5-OP-RU promotes the MAIT1 program

Most MAIT cells in WT B6 and B6-MAIT^CAST^ mice express retinoic acid receptor-related orphan receptor γt (RORγt) consistent with a MAIT17 phenotype [61]. There also exists a smaller MAIT1 population expressing T-box expressed in T cells (T-bet). These transcription factors govern MAIT cells’ immunomodulatory activities and inflammatory cytokine profiles. Therefore, we were curious to know whether vaccination with PR8 and 5-OP-RU reprograms MAIT cells.

**Fig 7:**
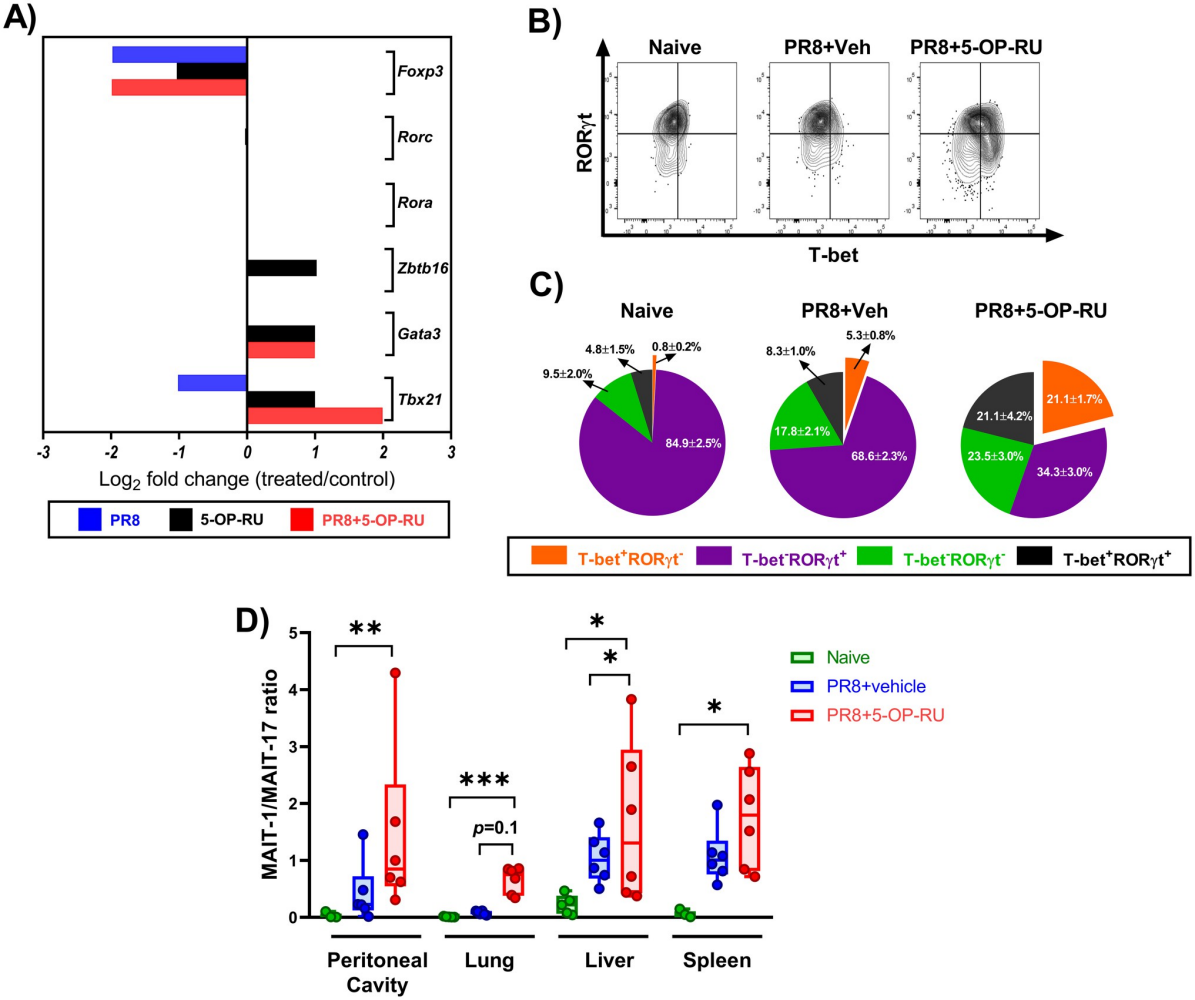
Immunization with PR8 plus 5-OP-RU promotes a MAIT1 program at transcriptional and protein levels. (**A**) B6-MAIT^CAST^ mice were injected *i*.*p*. with PBS (n = 10), PR8 (n = 10), 5-OP-RU (n = 10), or PR8 plus 5-OP-RU (n = 5). Three days later, pooled pulmonary MAIT cells were purified and subjected to RNA extraction, cDNA generation and quantitative PCR to assess the expression of indicated transcription factors. Gene expression fold changes in MAIT cells from each cohort relative to those isolated from control (PBS-injected) animals were calculated using the 2^-(ΔΔCt)^ method. (**B-C**) In additional experiments, B6-MAIT^CAST^ mice were left unimmunized (n = 5) or inoculated *i*.*p*. with PR8 plus 5-OP-RU (or vehicle) (n = 6/group). Three days later, pulmonary MAIT cells were interrogated by flow cytometry for their intracellular RORγt and T-bet contents. Representative plots illustrate the staining for the above transcription factors within TCRβ^+^ MR1 tetramer^+^ cells after using isotype controls to set quadrant gates (**B**), and pie charts depict the frequencies (± SEM) of T-bet^+^RORγt^-^, T-bet^-^RORγt^+^, T-bet^+^RORγt^+^ and T-bet^-^RORγt^-^ MAIT cell subsets (**C**). The percentages of T-bet^+^RORγt^-^ and T-bet^-^RORγt^+^ MAIT cells were used to calculate MAIT1:MAIT17 ratios in indicated sites for each cohort (**D**). Each circle in Box-and-Whisker plots corresponds to an individual animal (or pooled peritoneal sample). Data shown in panels **C** and **D** are from five independent experiments. Statistical comparisons were performed using the Kruskal-Wallis test followed by the Dunn’s post-hoc test. *, ** and *** denote differences with *p* ≤ 0.05, *p* ≤ 0.01 and *p* ≤ 0.001, respectively.

In immunophenotyping experiments, we found the vast majority of expanded MAIT cells to be low CD103 (S11A and S11B Fig) and high CD122 expressors (S12 Fig), suggesting a functional MAIT1 lineage bias [62,63]. Moreover, apart from their increased *Il2rb* transcript levels, the expanded population showed diminished *Il23r* and *Ccr6* (S13 and S14 Figs) and unaltered *Icos* expression (S15 Fig), all of which can be viewed as MAIT1 characteristics [62,63]. In fact, *Tbx21*, which encodes T-bet, was strongly upregulated in MAIT cells following vaccination with PR8 plus 5-OP-RU (Fig 7A).

To define MAIT1 and MAIT17 subsets unequivocally, we assayed for intracellular transcription factors. As expected, T-bet^-^RORγt^+^ cells were the prominent MAIT subset in the lungs (Fig 7B and 7C), liver, spleen and peritoneal cavity of B6-MAIT^CAST^ mice (S16 Fig) whereas T-bet^+^RORγt^-^ and T-bet^+^RORγt^+^ subpopulations were minorities. Moreover, PR8 immunization without 5-OP-RU elevated T-bet^+^ cell frequencies only marginally. Remarkably, however, 5-OP-RU-adjuvanted vaccination gave rise to a large T-bet^+^ subset (Fig 7B and 7C and S16 Fig). As a result, a significantly higher MAIT1:MAIT17 ratio emerged (Fig 7D), indicating a skewed response towards T_H_1-type cytokine production. We also detected a T-bet^-^RORγt^-^ MAIT cell subset at varying frequencies across different tissues and cohorts (Fig 7B and 7C and S16 Fig). However, we ruled out the possibility that GATA binding protein 3 (GATA-3)^+^ cells were a major component of this double-negative subpopulation (S17 Fig).

**Fig 8:**
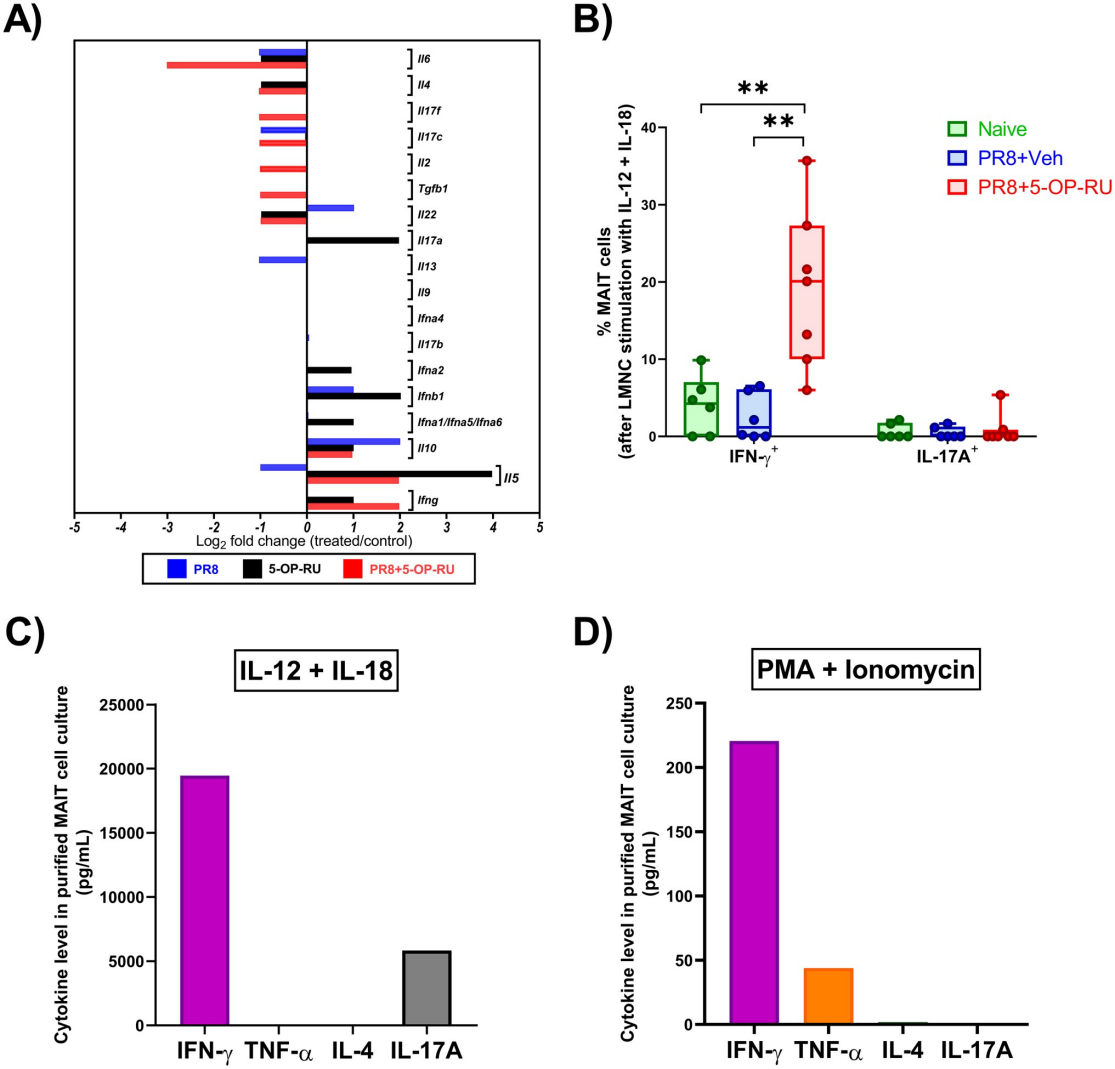
Co-administration of PR8 and 5-OP-RU confers upon MAIT cells a T_H_1-type cytokine profile. (**A**) B6-MAIT^CAST^ mice were injected *i*.*p*. with PBS (n = 10), PR8 (n = 10), 5-OP-RU (n = 10), or PR8 plus 5-OP-RU (n = 5). Three days later, the expression of indicated cytokines by pulmonary MAIT cells was quantified by real-time PCR for each cohort relative to PBS-injected animals. (**B**) Unfractionated non-parenchymal lung mononuclear cells (LMNCs) from naïve mice (n = 6), animals immunized with PR8 plus 5-OP-RU (n = 7), and animals immunized with PR8 plus vehicle (n = 6) were isolated in five independent experiments and stimulated *ex vivo* with recombinant mouse IL-12 and IL-18. After 18 hours, the frequencies of IFN-γ^+^ and IL-17A^+^ cells among TCRβ^+^ MR1 tetramer^+^ MAIT cells were determined by flow cytometry. One-way ANOVA was used, followed by the Tukey’s Multiple Comparison test, for statistical comparisons. ** denotes significant differences with *p* ≤ 0.01. (**C-D**) Pulmonary MAIT cells were purified from mice previously injected with PR8 and 5-OP-RU (n = 10), pooled and stimulated with either a combination of IL-12 and IL-18 (**C**) or a combination of PMA and ionomycin (**D**). After 24 hours, cytokines levels were measured by ELISA in culture supernatants.

Global cytokine gene expression analyses demonstrated increased *Ifng* and decreased *Il4* levels in MAIT cells after immunization with PR8 and 5-OP-RU (Fig 8A). To determine the extent to which MAIT cells were skewed, we stimulated bulk MNCs from naïve and vaccinated mice to the T_H_1-polarizing cytokines IL-12 and IL-18 before measuring MAIT cells’ IFN-γ and IL-17A contents. We found significantly higher IFN-γ^+^ cell percentages in mice that had received 5-OP-RU (Fig 8B and S18 Fig). By comparison, IL-17A^+^ MAIT cell frequencies were always low and comparable in the three experimental groups.

To assess MAIT cells’ cytokine production capacity in the absence of transactivating signals provided by other cell types, we incubated purified MAIT cells from mice that had received PR8 and 5-OP-RU with a panel of stimuli. First, exposure to IL-12 and IL-18 generated a large quantity of IFN-γ, which was ~4 times more than IL-17A (Fig 8C), consistent with cytofluorimetric data obtained from unfractionated MNC cultures (Fig 8B and S18 Fig). In contrast, using plate-coated anti-CD3 to cross-link *i*TCRs, alone or in combination with an anti-CD28 mAb, resulted in IFN-γ and IL-17A secretion (S19A and S19B Fig). Therefore, a prior *in vivo* exposure to 5-OP-RU had not impeded MAIT cells’ ability to signal through their Ag receptors.

To examine MAIT cells’ polarization in an unbiased manner, without relying on *i*TCR and cytokine receptor signaling, we used a combination of PMA and ionomycin. PMA activates protein kinase C and ionomycin releases Ca^++^ from intracellular stores, thus inducing cytokine secretion [64]. In these experiments, purified MAIT cells from vaccinated animals produced large quantities of IFN-γ and some tumor necrosis factor (TNF)-α, but no detectable IL-4 or IL-17A (Fig 8D).

Collectively, the above results indicate that vaccination with PR8 and 5-OP-RU expands, activates and skews MAIT cells towards a MAIT1 phenotype, which should favor antiviral immunity.

### Incorporating 5-OP-RU in an anti-IAV vaccination protocol affords heterosubtypic protection against IAV infection

**Fig 9:**
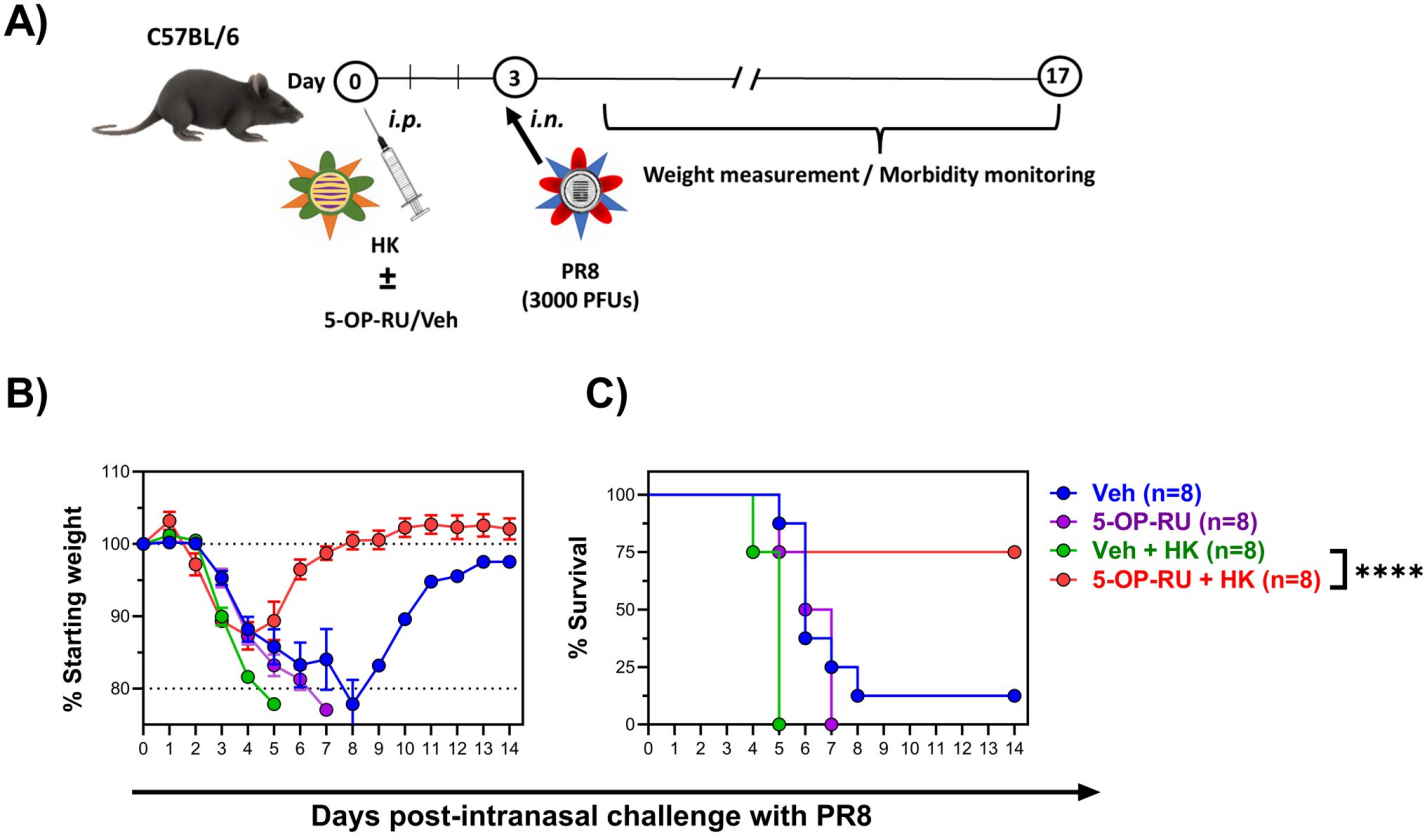
Vaccination with 5-OP-RU-adjuvanted IAV protects against a subsequent heterosubtypic challenge. (**A**) In two independent experiments, B6 mice were injected *i*.*p*. with the H3N2 IAV strain HK, with 5-OP-RU (or vehicle), or with a combination of HK and 5-OP-RU (or vehicle). Three days later, animals were challenged with an intranasal (*i*.*n*.) PR8 inoculum and monitored for weight loss (**B**) and mortality (**C**). Chi-squared tests were used in statistical analyses. **** denotes a significant difference with *p* ≤ 0.0001.

To test the above hypothesis, we sought to explore the adjuvanticity of 5-OP-RU in a vaccination model in which *i*.*p*. priming with HK, which is not pathogenic in mice, precedes an *i*.*n*. challenge with the mouse-adapted PR8 strain (Fig 9A).

As expected, MAIT cells amassed in several tissues, including in the lungs, 3 days after the *i*.*p*. inoculation of WT B6 mice with HK and 5-OP-RU (S20 Fig). In these experiments, administering HK or 5-OP-RU alone failed to prevent PR8-induced weight loss and mortality (Fig 9B and 9C). However, a combination of HK and 5-OP-RU protected the animals. Therefore, 5-OP-RU can be used as an adjuvant in anti-IAV immunization strategies.

### Adding 5-OP-RU to *i.n.* and *i.m.* vaccines for influenza and COVID-19 elevates MAIT cell numbers and augments CD8^+^ T_conv_ cell responses to immunodominant viral epitopes

**Fig 10:**
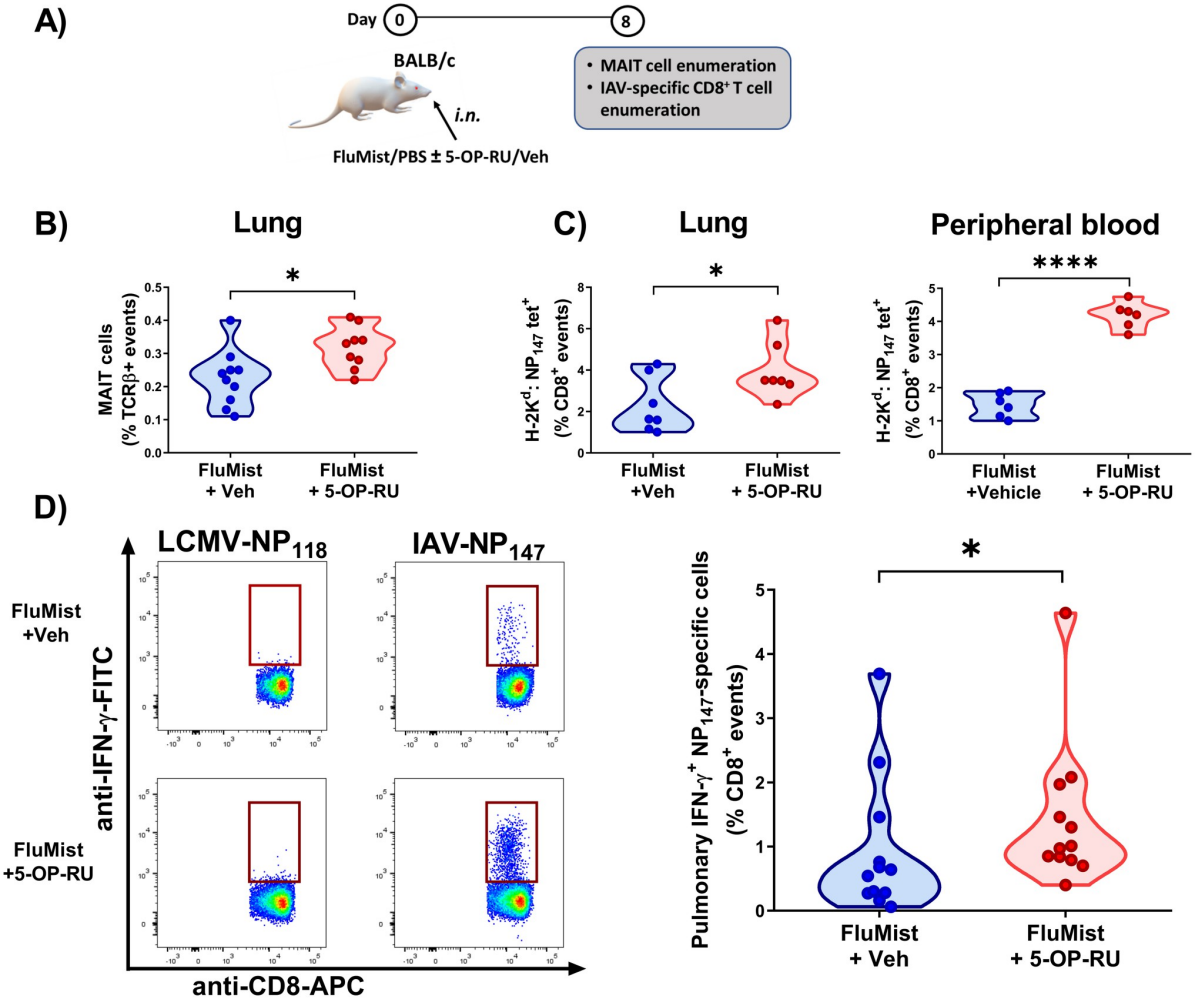
Intranasal co-administration of FluMist and 5-OP-RU to BALB/c mice increases the frequencies of pulmonary MAIT cells and immunodominant IAV-specific CD8^+^ T cells. (**A**) In three independent experiments, BALB/c mice were inoculated intranasally (*i*.*n*.) with the live attenuated influenza vaccine FluMist plus 5-OP-RU (or vehicle). Eight days later, pulmonary MAIT cells were enumerated by MR1 tetramer staining (**B**), and NP_147_-specific CD8^+^ T cell frequencies were determined by MHC I tetramer staining in the lungs and in the peripheral blood (**C**). (**D**) In parallel or in addition, immunodominant NP_147_-specific CD8^+^ T cells were identified by intracellular IFN-γ staining after non-parenchymal lung mononuclear cells from vaccinated animals were stimulated *ex vivo* with TYQRTRALV. LMNCs were also incubated with an irrelevant peptide (RPQASGVYM) corresponding to the most immunodominant epitope of the lymphocytic choriomeningitis virus nucleoprotein (LCMV-NP_118_). Representative plots and summary data are depicted (**D**). Each circle represents an individual mouse (**B-D**). Statistical analyses were conducted using unpaired *t*-tests (**B-C**) and Mann-Whitney *U* test (**D**). * and **** denote differences with *p* ≤ 0.05 and *p* ≤ 0.0001, respectively.

To test the efficacy of 5-OP-RU in inducing mucosal immunity, we inoculated BALB/c mice *i*.*n*. with the live attenuated influenza vaccine FluMist (Fig 10A). We used BALB/c mice in this model because the IAVs used in FluMist, similarly to the PR8 strain, harbor an immunodominant peptide called NP_147-155_, which is restricted by H-2K^d^ and therefore detectable in this mouse strain [65]. As such, these experiments enabled us to enumerate not only MAIT cells but also NP_147_-specific CD8^+^ T_conv_ cells.

As with our *i*.*p*. vaccination models, combining FluMist with 5-OP-RU raised MAIT cell frequencies in the lungs (Fig 10B). Furthermore, MHC class I tetramer staining revealed increases in pulmonary and peripheral blood NP_147_-specific T_conv_ cell numbers (Fig 10C). These were functional, Ag-specific T cells as judged by their ability to produce IFN-γ upon brief *ex vivo* stimulation with a synthetic peptide corresponding to NP_147-155_, but not an irrelevant peptide derived from LCMV (NP_112-126_), in ICS assays (Fig 10D).

**Fig 11:**
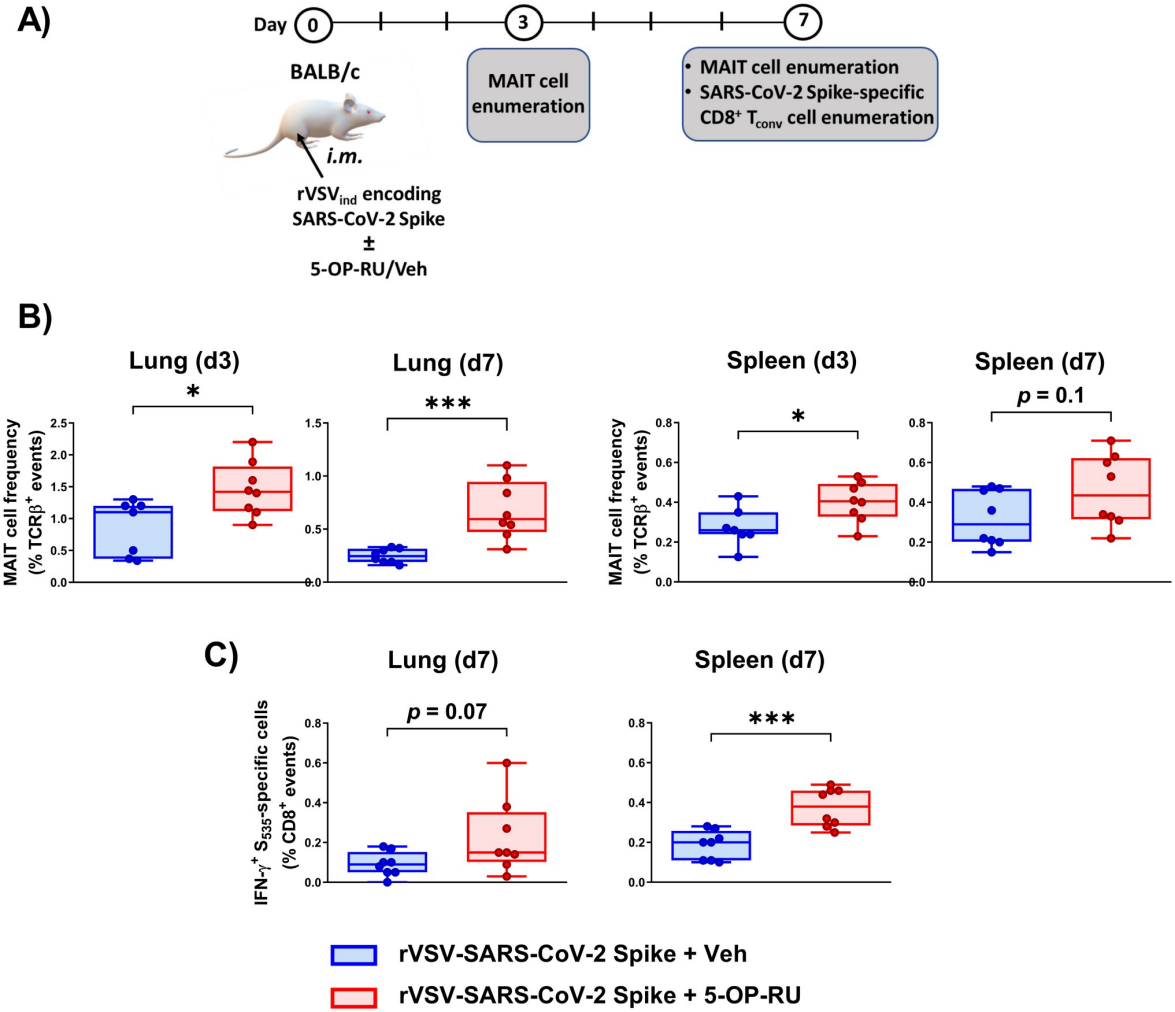
Intramuscular co-administration of an rVSV-based COVID-19 vaccine and 5-OP-RU raises the frequencies of pulmonary and splenic MAIT cells and cognate CD8^+^ T cell. In two independent experiments, BALB/c mice were injected intramuscularly (*i*.*m*.) with rVSV_Ind_ expressing the SARS-CoV-2 Spike (S) gene plus 5-OP-RU (or vehicle) (**A**). Three or 7 days later, pulmonary and splenic MAIT cells were enumerated by MR1 tetramer staining (**B**). On day 7 post-immunization, the percentages of S_535_-specific CD8^+^ T cells were also determined by intracellular cytokine staining for IFN-γ (**C**). Each circle in Box-and-Whisker plots represents an individual mouse. * and *** denote statistically significant differences with *p* ≤ 0.05 and *p* ≤ 0.001, respectively, by unpaired *t*-tests.

Next, we evaluated the adjuvanticity of 5-OP-RU in *i*.*m*. immunization against another respiratory pathogen. To this end, BALB/c mice were injected in the hind limb with an rVSV-vectored vaccine candidate encoding the SARS-CoV-2 Spike (Fig 11A), which we recently generated [37]. In this setting, we again found more MAIT cells in mice receiving 5-OP-RU on days 3 and 7 post-vaccination, especially in the lungs (Fig 11B). In addition, cognate CD8^+^ T_conv_ cells recognizing an immunodominant determinant of the SARS-CoV-2 Spike (S_535-543_) [47] were more abundant in animals that had received 5-OP-RU (Fig 11C and S21 Fig).

In a limited number of experiments, we included 5-OP-RU or vehicle during *i*.*m*. priming with rVSV_Ind_-Spike and *i*.*m*. boosting with rVSV_NJ_-Spike. Using this prime-boost immunization model, a significant increase in pulmonary and splenic MAIT cell numbers was noticeable in the cohort that had received 5-OP-RU during both phases (S22 Fig).

### Exposing human PBMCs to propagating PR8 virions and 5-OP-RU results in MAIT cell activation and proliferation

**Fig 12:**
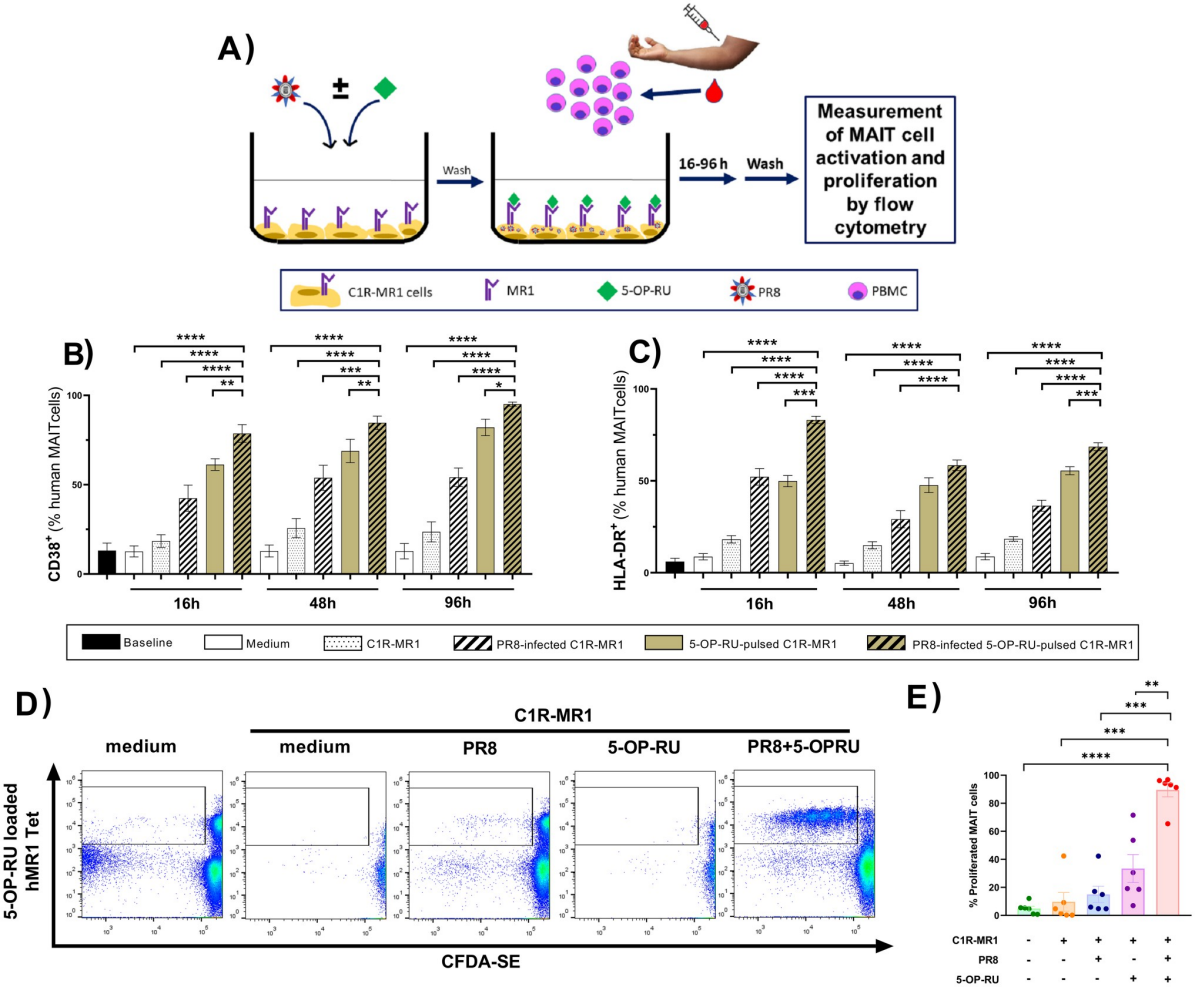
*In vitro* stimulation of human PBMCs with live PR8 and 5-OP-RU activates and expands MAIT cells. Human peripheral blood mononuclear cells (PBMCs) from healthy donors were incubated in complete medium (n = 6) or stimulated with C1R-MR1 cells that had been infected with PR8 and/or pulsed with 5-OP-RU (n = 10 per condition) (**A**). At indicated timepoints, the frequencies of CD38^+^ (**B**) and HLA-DR^+^ (**C**) cells among CD3^+^ hMR1 tetramer^+^ MAIT cells were determined by flow cytometry. (**D-E**) In additional experiments, PBMCs were labelled with CFDA-SE before they were stimulated as described above. Seven days later, CFDA-SE dye dilution by hMR1 tetramer-positive (MAIT) and -negative (non-MAIT T) cells was assessed after gating on CD3^+^ events. Representative plots (**D**) and summary data (n = 6 donors) (**E**) are depicted. Data are shown as mean ± SEM (**B, C and E**). Statistical comparisons were made using Repeated Measures one-way ANOVA with the Dunnett’s Multiple Comparisons test (**B-C**) and Friedman tests followed by Dunn’s post-hoc analyses (**E**). *, **, *** and **** denote differences with *p* ≤ 0.05, *p* ≤ 0.01, *p* ≤ 0.001 and *p* ≤ 0.0001, respectively.

To extend our studies to human MAIT cells, we cultured PBMCs with uninfected or PR8-infected C1R-MR1 cells in the absence or presence of 5-OP-RU before we determined the expression levels of several activation markers (Fig 12A). We found significant upregulation of CD38 (Fig 12B) and human leukocyte antigen (HLA)-DR (Fig 12C) in cultures containing PR8-infected, 5-OP-RU-pulsed C1R-MR1 cells. This was clearly evident as early as 16 hours after the cultures were initiated and prevailed at least for 4 days (Fig 12B and 12C).

In separate cultures, we used CFDA-SE-labeled PBMCs in order to test the proliferative capacity of human MAIT cells. Seven days later, CFDA-SE dye dilution, indicating up to six cell division cycles, was detectable when PR8-infected, 5-OP-RU-pulsed C1R-MR1 cells were present in cultures (Fig 12D and 12E). This response was exclusive to MAIT cells since non-MAIT T cells did not divide (Fig 12D). The *in vitro* nature of these experiments also reinforces the idea that MAIT cell expansion can be due primarily, if not purely, to their inherent proliferative activity after they are exposed to IAVs and 5-OP-RU.

## Discussion

The presence and activation status of MAIT cells influences antiviral immune responses and the severity of viral diseases, with both protective and pathogenic roles demonstrated or proposed for these powerful lymphocytes [21,22]. For instance, while MAIT cells are thought to contribute to anti-IAV immunity [12,14], their CD69 or HLA-DR levels have been reported to predict the severity or mortality of COVID-19 in most, but not all, studies conducted on the subject to date [66–70].

MAIT cells are MR1-restricted T cells with potent antibacterial activities. Therefore, it is only fitting that administration of live bacterial vaccine strains or the MR1 ligand 5-OP-RU promotes protection against *Legionella longbeachae* [71], *Francisella tularensis* [72], and *Vibrio cholerae* [73]. However, since viruses do not generate MR1 ligands, our initial observation that 5-OP-RU synergized with IAV vaccines to potentiate MAIT cell accumulation in multiple tissues was intriguing. Ki-67 staining, *in vivo* S1PR1 antagonism, and adoptive transfer experiments collectively demonstrated that MAIT cell proliferation, rather than their emigration from other sites, was responsible for the above phenomenon, a finding that could also be recapitulated in human MAIT cell cultures. Of note, in a similar PBMC culture system, Lamichhane *et al*. [74] found PR8 to augment *i*TCR-mediated MAIT cell activation and degranulation as judged by their increased CD69, PFN, GZM B and CD107a levels. However, no MAIT cell proliferation was reported. This may be due to the fact that the PR8 virions were subjected to ultraviolet radiation by these investigators. By contrast, we used replication-competent PR8, which induced MAIT cell proliferation.

Several lines of evidence point to a requirement for dsRNA-sensing pattern recognition receptors (PRRs), such as TLR3, in our system. First, HI-PR8, which is immunogenic but unable to propagate, failed to synergize with 5-OP-RU to induce peritoneal MAIT cell proliferation. Therefore, live viral replication, which generates dsRNA, appeared to be necessary for the observed MAIT cell response. We are cognizant of the previous reports that negative-sense RNA viruses like PR8 are not effective producers of dsRNA [75–77]. However, the fact that MAIT cell expansion was evident when 5-OP-RU was combined with a variety of viruses and vaccines, including multiple influenza strains (*i*.*e*., PR8, NT60, HK, X31 and FluMist virions), a vaccine vector based on a different negative-strand RNA virus (*i*.*e*., VSV), and a DNA virus (*i*.*e*., VacV), supports the notion that a common pathogen-associated molecular pattern (PAMP) was at play. We posited that viral dsRNA and TLR3 were the PAMP and the PRR involved, respectively. Indeed, poly (I:C), a synthetic mimic of dsRNA that is sensed by TLR3, induced MAIT cell proliferation when co-administered *i*.*p*. with 5-OP-RU. This finding is consistent with a previous report that *i*.*n*. inoculation of poly (I:C) before 5-OP-RU expands pulmonary MAIT cells in WT B6 mice [78]. Based on our preliminary experiments, naked low-molecular-weight poly (I:C) may be more efficient than LyoVec-complexed high-molecular-weight poly (I:C) in this capacity. Therefore, melanoma differentiation-associated gene 5 (MDA5), a cytosolic PRR that recognizes long dsRNA [79], seems not to play a prominent role in MAIT cell expansion in our model. We are currently studying the molecular intermediates of intracellular signaling pathways that contribute to or control MAIT cell responses, including TLR3- and possibly retinoic acid-inducible gene-I (RIG-I)-coupled pathways.

A key consequence of TLR3 engagement is the production of TI-IFNs, to which MAIT cells react [11,13,27]. The elevated blood levels of IFN-β in mice receiving PR8 plus 5-OP-RU, and the observed synergy between 5-OP-RU and either rmIFNα1 or rmIFNβ implicated IFNAR signaling in our experimental systems. This was confirmed by using an IFNAR-1-blocking mAb, which prevented MAIT cell proliferation. We are currently investigating whether IFNAR signaling is required in a MAIT cell-intrinsic or -extrinsic fashion in different mouse tissues although these possibilities may not be mutually exclusive. In a previous study, exposing purified human peripheral blood Vα7.2^+^ cells, which contain a large MAIT cell fraction, to IFN-α or IFN-β enhanced the IFN-γ and GZM B production capacities of these cells in response to anti-CD3/CD28-coated beads [74]. This lends support to the importance of MAIT cell-autonomous IFNAR signaling for the generation of certain effector molecules.

How exactly *i*TCR triggering synergizes with TI-IFNs to expand tissue MAIT cell populations is speculative at this point. According to Stevens *et al*. [80], some of the early features of TCR- and IFNAR-coupled cascades, including mitogen-activated protein kinase (MAPK) phosphorylation, are identical in Jurkat cells and primary peripheral blood T_conv_ cells. However, TI-IFN-induced MAPK activation is relatively short-lived and does not induce the same transcriptional responses that follow TCR stimulation. Therefore, the possibility that 5-OP-RU and TI-IFNs generate cell division-promoting signals that merge propitiously and culminate in MAIT cell proliferation seems far-fetched. During LCMV infection, TI-IFNs act directly on antiviral CD8^+^ T_conv_ cells to keep them alive in the Ag-specific clonal expansion phase [81,82]. By extrapolating from these findings, one could envisage a scenario in which IFNAR signaling in MAIT cells allows them to survive in order to receive and react to a cognate proliferative signal that is transmitted upon 5-OP-RU recognition. Consistent with this theory, there was no indication of proneness to apoptotic death in expanded MAIT cells in our gene expression analyses (S23 Fig).

While many cell types release TI-IFNs during viral infections or vaccination, plasmacytoid DCs (pDCs) are particularly efficient in producing copious amounts of these cytokines [83,84]. Our current understanding of how pDCs may communicate with MAIT cells is rudimentary. The immunogenicity of adenovirus-based vaccine vectors was recently reported to depend on MAIT cells whose activation in turn required IFN-α production by pDCs [27]. Unlike myeloid DCs, pDCs rely on TLR7, not TLR3, to sense the presence of viruses [85,86], and the TLR7 agonist imiquimod did not synergize with 5-OP-RU to expand MAIT cells. Therefore, pDCs are unlikely to play a critical role in our models.

Provine *et al*. [27] investigated the activation status of MAIT cells and their potential targetability when using replication-incompetent vectors. In contrast, we have explored and documented the adjuvanticity of an MR1 ligand, namely 5-OP-RU, using multiple immunization modes and routes. Furthermore, we demonstrate that MAIT cell proliferation in vaccination settings requires replication-competent vaccine strains or vectors. This may be why Provine *et al*. did not report MAIT cell expansion in their study.

Importantly, 5-OP-RU-adjuvanted IAV vaccines not only expanded MAIT cells but also protected against a lethal challenge with PR8. This may be attributable to a skewed MAIT1 response characterized by T-bet expression and pro-inflammatory cytokine production, which should enable MAIT cells to transactivate a number of antiviral secondary effectors, including NK cells and CD8^+^ T_conv_ cells. In fact, in our FluMist and rVSV-based immunization models for influenza and COVID-19, respectively, we had the opportunity to enumerate immunodominant CD8^+^ T_conv_ cells, which were markedly increased in animals receiving 5-OP-RU-adjuvanted vaccines.

In our prime-boost anti-IAV immunization protocol, a secondary exposure to 5-OP-RU was sufficient to induce tissue MAIT cell accumulation (Fig 2 and S4 Fig). In our rVSV-based protocol for COVID-19, the optimal response was achieved when 5-OP-RU was given during both the priming and the boosting phase (S22 Fig). These experiments clearly demonstrate that 5-OP-RU inoculation does not render MAIT cells unresponsive to subsequent 5-OP-RU administrations. Consistent with these observations, we found no transcriptional signs of overt cellular exhaustion in MAIT cells from animals that had received 5-OP-RU-adjuvanted PR8 (S15 Fig). In fact, *Pdcd1* was the only activation/exhaustion-associated gene that was markedly upregulated, albeit to the same extent as in mice receiving 5-OP-RU alone. These studies will be extended in the future to include additional genes. Regardless, repeated 5-OP-RU administration, when/if necessary, should be feasible in preventative or even in therapeutic vaccination strategies. This is in sharp contrast with *i*NKT cells, sometimes dubbed as the mouse cousins of human MAIT cells, which undergo long-term anergy via a programmed death-1 (PD-1)/programmed death ligand-1 (PD-L1)-dependent mechanism in animals receiving a single injection of the prototypic CD1d ligand α-galactosylceramide (α-GalCer) [87,88]. As a result, anergic *i*NKT cells lose their responsiveness to future encounters with the same glycolipid Ag and fail to produce several cytokines, including IFN-γ. This may explain, at least partially, why repeated injections of free-floating α-GalCer have not worked optimally in clinical trials for solid tumors and viral diseases [89]. Future work will need to address the most tolerable and productive 5-OP-RU dosages, injection frequencies and time intervals.

MAIT cells have been implicated in B cell help and antibody responses [28,30]. Therefore, in a limited set of experiments, we compared *Mr1*^-/-^ and *Mr1*^+/+^ B6-MAIT^CAST^ mice for their humoral responses to PR8. These experiments revealed no marked differences in PR8-specific IgM and IgG blood levels between MAIT-deficient and -sufficient animals (S24 Fig). As such, in their steady state, MAIT cells appear dispensable for systemic humoral immunity to PR8. In addition, incorporating 5-OP-RU in our immunization strategy did not raise PR8-specific antibody titres in the serum (S25 Fig). Therefore, the protective effect of 5-OP-RU as a non-classical vaccine adjuvant may lie within its ability to augment cognate CD8^+^ T_conv_ cell responses that clear established infections, and perhaps innate effector cells (*e*.*g*., NK cells and macrophages). Whether MAIT cells and MR1 ligands enhance vaccine-elicited antibody responses in mucosal sites has been a subject of recent investigations [30,73,90] but remains to be fully elucidated.

We found 5-OP-RU to expand MAIT cells in young and old mice, and in female and male animals of different MHC backgrounds. The evolutionarily conserved nature of MAIT cells and their selection mode [91], the similarities found between mouse and human MAIT cells [92], and the fact that 5-OP-RU activates MAIT cells in both species collectively suggest that at least some of our findings should be translatable to clinical settings. Moreover, since MR1 is monomorphic, its ligands should work in genetically diverse human populations. This is unlike T_conv_ cell-based interventions that need to be tailored to individual HLA backgrounds.

Opportunistic bacterial and fungal infections are among the most catastrophic complications of viral infections. For instance, community-acquired and nosocomial bacterial pneumonia claim many lives after IAV and SARS-CoV-2 infections [93,94]. MAIT cells should help clear such infections by at least four mechanisms. First, their stimulation bolsters other innate and adaptive effectors with antibacterial activities [25–29]. Second, MAIT cells directly recognize and kill infected cells displaying bacterial (and fungal) MR1 ligands [2,31]. Third, cytolytic effector molecules within the MAIT cells’ arsenal may damage free-living extracellular bacteria, and synergize with the bactericidal activities of certain antibiotics such as carbapenems [32]. Of note, our global gene expression analyses revealed upregulated *Gzmb*, *Gzmk* and *Fasl* transcript levels in MAIT cells from mice receiving PR8 plus 5-OP-RU (S26 Fig). These cytolytic effector molecules should help augment MAIT cells’ antibacterial functions. Finally, MAIT cell roles in tissue repair and remodeling [38,95–98] should help resolve structural impairments that may elevate the risks of continued or *de novo* infections. For all of the above reasons, we propose that reinstating or reinforcing tissue MAIT cell compartments will protect against secondary infections and superinfections. Importantly, the above functions are typically, if not exclusively, dependent on the MR1/*i*TCR-dependent pathway, which is targeted by 5-OP-RU. Efforts to expand and store MAIT cells as donor MHC-unrestricted, off-the-shelf immunotherapeutics are underway [43]. Whether our *in vivo* findings that poly (I:C) and TI-IFNs enhance the ability of 5-OP-RU to expand MAIT cells can be extended to *in vitro* platforms for future human cell therapies is a subject of ongoing work.

In summary, although MR1 ligands are not generated by viruses and virus-based vaccines, we have herein demonstrated that MR1 can be targeted by 5-OP-RU towards more efficacious antiviral immunization strategies. 5-OP-RU synergizes with multiple vaccines to expand pro-inflammatory MAIT1 cells in a TLR3/IFNAR-dependent manner, to enhance antiviral CD8^+^ T_conv_ cell responses, and to enable heterosubtypic anti-IAV immunity. Therefore, we propose 5-OP-RU as a potent and versatile vaccine adjuvant against respiratory viral pathogens.

## Supporting information

S1 Table TaqMan-based quantitative PCR primer/probe sets used in this investigation. (PDF)

S2 Table List of Antibodies, tetramers and other reagents. (PDF)

S1 Data Raw data obtained in this study. (XLSX)

S1 Fig Representative cytofluorimetric plots depicting pulmonary MAIT cells prior to and after magnetic purification. Five B6-MAIT^CAST^ mice were injected *i*.*p*. with a combination of PR8 and 5-OP-RU. Three days later, non-parenchymal lung mononuclear cells (LMNCs) were isolated, pooled and stained with phycoerythrin (PE)-conjugated, 5-OP-RU-loaded mouse MR1 tetramers. MAIT cells were then magnetically purified using anti-PE microbeads as detailed in Materials and Methods. MAIT cell percentages among bulk LMNCs (pre-sort) and after two rounds of column purification (post-sort) are shown.
(TIF)

S2 Fig Intraperitoneal MAIT cell accumulation after immunization with PR8 and 5-OP-RU lasts longer than three days. B6-MAIT^CAST^ mice were inoculated *i*.*p*. with PR8, 5-OP-RU, or a combination of PR8 and 5-OP-RU (or vehicle) followed, 7 days later, by cytofluorimetric enumeration of MAIT cells in the peritoneal cavity. Peritoneal MAIT cell frequencies and absolute numbers are depicted. Each circle corresponds to an individual animal, and data are presented as mean ± SEM. Statistical comparisons were made using the one-way ANOVA followed by the Dunnett’s post-hoc Multiple Comparisons test. ** and *** signify differences with *p* ≤ 0.01 and *p* ≤ 0.001, respectively.
(TIF)

S3 Fig Immunization with 5-OP-RU-adjuvanted PR8 does not affect tissue *i*NKT cell frequencies. Naïve B6-MAIT^CAST^ mice (n = 5) and mice that had received PR8 plus 5-OP-RU (or vehicle) three days earlier (n = 6) were sacrificed for their lungs (**A and D**), liver (**B** and **D**) and spleen (**C-D**) in which TCRβ^+^ PBS-57-loaded mCD1d tetramer^+^ *i*NKT cells were enumerated. Empty mCD1d tetramers were used in parallel to draw cytofluorimetric gates (**A-C**). Representative plots (**A-C**) and summary data with mean ± SEM values (**D**) are shown. Group comparisons were made using the Kruskal-Wallis test followed by the Dunn’s post-hoc analysis.
(TIF)

S4 Fig 5-OP-RU elevates pulmonary MAIT cell numbers when administered during boosting immunization against IAV. B6 mice (n = 3–4) were primed *i*.*p*. with PR8 and boosted, six weeks later, with an *i*.*p*. inoculum of the reassortant X31 strain plus 5-OP-RU (or vehicle). Additional cohorts received 5-OP-RU or vehicle alone as indicated. Three days after the secondary injection/immunization, MAIT cells were enumerated by flow cytometry in the lungs. Each circle represents an individual animal. *** denotes a statistically significant difference with *p* ≤ 0.001 by one-way ANOVA followed by the Holm-Šidék Multiple Comparisons test.
(TIF)

S5 Fig Incorporating 5-OP-RU into boosting anti-IAV immunization elevates the frequency of hepatic MAIT cells. B6-MAIT^CAST^ mice were primed *i*.*p*. with PR8 (H1N1) and boosted *i*.*p*., four weeks later, with X31 (H3N2). 5-OP-RU (or vehicle) was co-administered in both phases. Three days after secondary immunization, hepatic (**A**) and splenic (**B**) MAIT cells were enumerated by flow cytometry. Each circle represents an individual mouse, and data are shown as mean ± SEM. * and ** denotes a significant difference with *p* ≤ 0.05 and *p* ≤ 0.01, respectively, using one-way ANOVA followed by the Tukey’s Multiple Comparisons test.
(TIF)

S6 Fig Intraperitoneal co-inoculation of PR8 and 5-OP-RU upregulates CD69 expression by pulmonary MAIT cells. B6-MAIT^CAST^ mice (n = 5/group) were injected *i*.*p*. with PR8 plus 5-OP-RU or vehicle. Three days later, CD69^+^ MAIT cell frequencies in the lungs and the geometric mean fluorescence intensity (gMFI) of CD69 staining were determined by flow cytometry. ** denotes significant differences with *p* ≤ 0.01 by unpaired *t*-tests.
(TIF)

S7 Fig Interfering with S1PR1 signaling does not prevent peritoneal or hepatic MAIT cell expansion in mice receiving PR8 plus 5-OP-RU. B6-MAIT^CAST^ mice were injected *i*.*p*. with FTY720 (or vehicle) 2 hours before and 22 hours after immunization with 5-OP-RU-adjuvanted PR8, followed by TCRβ^+^ MR1 tetramer^+^ MAIT and TCRβ^+^ MR1 tetramer^-^ non-MAIT T cell enumeration in the peritoneal cavity (left panel) and liver (right panel) on day 3 post-immunization. Each circle represents an individual mouse. Data were pooled from four (left panel) or three (right panel) independent experiments yielding similar results, and * denotes *p* ≤ 0.05 by Mann-Whitney *U* test.
(TIF)

S8 Fig MAIT cells proliferate *in vivo* following immunization with PR8 and 5-OP-RU. MAIT cells were magnetically purified out of pooled non-parenchymal pulmonary and hepatic mononuclear cells from naïve B6-MAIT^CAST^ mice, expanded as described in Materials and Methods, labeled with CellTrace Far Red dye, and adoptively transferred *i*.*v*. into B6-MAIT^CAST^ recipients. Twenty-four hours later, animals were injected *i*.*p*. with PBS or with a combination of PR8 and 5-OP-RU. After 3 days, mice were sacrificed for their lungs, liver and spleen in which CellTrace dye dilution by MAIT cells was examined by flow cytometry. The percentages of proliferated MAIT cells are shown for each organ.
(TIF)

S9 Fig Poly (I:C), but not imiquimod, partially simulates PR8 when combined with 5-OP-RU to expand MAIT cells. B6-MAIT^CAST^ mice were injected *i*.*p*. with 5-OP-RU in combination with PR8, poly (I:C) (50 μg/mouse) or imiquimod (50 μg/mouse). Three days later, peritoneal MAIT cell frequencies were determined by flow cytometry. Each symbol represents an individual animal. ** denotes significant differences with *p* ≤ 0.01 by the Mann-Whitney *U* test.
(TIF)

S10 Fig Cytokine serum levels after intraperitoneal administration of PR8 and/or 5-OP-RU. B6-MAIT^CAST^ mice were injected *i*.*p*. with PR8 (n = 12), 5-OP-RU (n = 12), or both (n = 12). After 1.5, 6 or 24 hours, animals were sacrificed (n = 4/timepoint) and their serum cytokine levels were measured. Statistical comparisons were made using unpaired *t*-tests. Asterisks (*) denote significant differences between sera from mice receiving PR8 plus 5-OP-RU and those injected with 5-OP-RU only. Hashtags (#) signify differences between animals receiving PR8 plus 5-OP-RU and those inoculated with PR8 only.
(TIF)

S11 Fig Tissue MAIT cells expanded via intraperitoneal immunization with PR8 and 5-OP-RU are low CD103 expressors. Naïve B6-MAIT^CAST^ mice and those receiving PR8, 5-OP-RU, or both, 3 or 7 days earlier were sacrificed before pulmonary, hepatic, peritoneal and splenic MAIT cells were assessed by flow cytometry for CD103 expression. Representative plots after gating on TCRβ^+^ cells (**A**) and summary data (**B**) are provided.
(TIF)

S12 Fig MAIT cells expanded after intraperitoneal co-administration of PR8 and 5-OP-RU abundantly express CD122 on their surface. A naïve B6-MAIT^CAST^ mouse and a mouse that had received PR8 plus 5-OP-RU three days earlier were sacrificed, and pulmonary and splenic MAIT cells were examined cytofluorimetrically for CD122 expression. Histograms corresponding to naïve mouse values are overlaid with those from the immunized animal.
(TIF)

S13 Fig Cytokine receptor gene expression by pulmonary MAIT cells after intraperitoneal administration of PR8 and/or 5-OP-RU. B6-MAIT^CAST^ mice were given an *i*.*p*. injection of PBS (n = 10), PR8 (n = 10), 5-OP-RU (n = 10), or PR8 plus 5-OP-RU (n = 5). Three days later, pooled pulmonary MAIT cells were purified and assessed for their transcript levels of indicated cytokine receptors by quantitative PCR. Gene expression fold changes for each cohort relative to the control (PBS-injected) cohort were calculated using the 2^-(ΔΔCt)^ method.
(TIF)

S14 Fig Comprehensive gene expression analysis of pulmonary MAIT cells after intraperitoneal administration of PR8, 5-OP-RU, or both. B6-MAIT^CAST^ mice were injected *i*.*p*. with PBS (n = 10), PR8 (n = 10), 5-OP-RU (n = 10), or PR8 plus 5-OP-RU (n = 5). Three days later, pooled pulmonary MAIT cells were isolated and examined by real-time PCR to quantitate the transcript levels of indicated molecules. Gene expression fold changes for each cohort were calculated relative to the control (PBS-injected) cohort using the 2^-(ΔΔCt)^ method.
(TIF)

S15 Fig Transcriptional examination of classic costimulatory, co-inhibitory and exhaustion-associated molecules expressed by pulmonary MAIT cells after intraperitoneal administration of PR8 and/or 5-OP-RU. B6-MAIT^CAST^ mice were given an *i*.*p*. injection of PBS (n = 10), PR8 (n = 10), 5-OP-RU (n = 10), or PR8 plus 5-OP-RU (n = 5). After 3 days, pulmonary MAIT cells were purified, pooled and assessed by quantitative PCR for their transcript levels of indicated molecules. Gene expression fold changes for each cohort were calculated in comparison with the PBS-injected (control) cohort as described in Materials and Methods.
(TIF)

S16 Fig Intraperitoneal immunization with PR8 plus 5-OP-RU skews peritoneal, splenic and hepatic MAIT cells towards a T-bet^+^RORγt^-^ phenotype. Naïve B6-MAIT^CAST^ mice (n = 5) and animals that had been inoculated 3 days earlier with PR8 plus 5-OP-RU (or vehicle) (n = 6/group) were sacrificed. Peritoneal, splenic and hepatic MAIT cells were then stained for intracellular T-bet and RORγt. Pie charts visualize the frequencies (± SEM) of T-bet^+^RORγt^-^, T-bet^-^RORγt^+^, T-bet^+^RORγt^+^ and T-bet^-^RORγt^-^ MAIT cell subsets.
(TIF)

S17 Fig PR8 and 5-OP-RU co-administration does not alter GATA-3^+^ MAIT cell frequencies in different tissues. Two naïve B6-MAIT^CAST^ mice and 12 animals that had received an *i*.*p*. injection of PR8 plus 5-OP-RU (or vehicle) (n = 6/group) three days earlier were sacrificed. Peritoneal, pulmonary, hepatic and splenic MAIT cells were interrogated by flow cytometry for their intracellular T-bet, RORγt and GATA-3 levels. Box-and-Whisker plots demonstrate GATA-3^+^ cell percentages among T-bet^-^RORγt^-^ MAIT cells.
(TIF)

S18 Fig *In vivo* co-administration of PR8 and 5-OP-RU gives rise to a higher percentage of MAIT cells capable of producing IFN-γ, but not IL-17A, in response to *ex vivo* stimulation with IL-12 and IL-18. Unfractionated peritoneal cells (**A**), non-parenchymal hepatic mononuclear cells (HMNCs) (**B**) and spleen cells (**C**) were obtained from indicated numbers of naïve and immunized B6-MAIT^CAST^ mice and stimulated *ex vivo* with recombinant mouse IL-12 and IL-18. After 18 hours, IFN-γ^+^ and IL-17A^+^ cell frequencies among TCRβ^+^ MR1 tetramer^+^ MAIT cells were determined by flow cytometry. Each circle corresponds to an individual sample. * and ** denote differences with *p* ≤ 0.05 and *p* ≤ 0.01, respectively, by unpaired *t*-tests (**A**) and one-way ANOVA followed by the Tukey’s Multiple Comparisons test (**C**).
(TIF)

S19 Fig Prior immunization with PR8 and 5-OP-RU does not compromise MAIT cells’ ability to respond to anti-CD3 alone or in combination with anti-CD28. Purified pulmonary MAIT cells were pooled from 10 B6-MAIT^CAST^ mice that had been inoculated *i*.*p*. with PR8 and 5-OP-RU three days earlier. Cells were stimulated with plate-coated anti-CD3 in the absence (**A**) or presence (**B**) of soluble anti-CD28 as described in Materials and Methods. Eighteen hours later, indicated cytokines were measured in culture supernatants by ELISA.
(TIF)

S20 Fig Intraperitoneal co-administration of A/Hong Kong/1/1968 and 5-OP-RU expands MAIT cells in several locations, including in the lungs. B6 mice (n = 3/group) were injected *i*.*p*. with the A/Hong Kong/1/1968 (HK) strain of IAV plus 5-OP-RU or vehicle. Three days later, peritoneal (left panel), pulmonary (middle panel) and splenic (right panel) MAIT cells were enumerated by flow cytometry. Unpaired *t*-tests were used for statistical comparisons. *, ** and *** indicate differences with *p* ≤ 0.05, *p* ≤ 0.01 and *p* ≤ 0.001, respectively.
(TIF)

S21 Fig Intramuscular administration of 5-OP-RU along with an rVSV-based COVID-19 vaccine elevates immunodominant Spike-specific CD8^+^ T cell numbers in the spleen. BALB/c mice were injected *i*.*m*. with rVSV_Ind_ expressing the SARS-CoV-2 Spike (S) gene plus 5-OP-RU (or vehicle). Seven days later, S_535_-specific CD8^+^ T cells were enumerated by intracellular cytokine staining for IFN-γ. Each circle represents an individual mouse, and ** denotes *p* ≤ 0.01 by unpaired *t*-test.
(TIF)

S22 Fig Robust MAIT cell expansion is achieved when 5-OP-RU is included during both primary and secondary immunizations with rVSV-based vaccines against SARS-CoV-2. BALB/c mice (n = 4 per group) were primed intramuscularly (*i*.*m*.) with rVSV_Ind_-Spike and boosted 24 days later (also *i*.*m*.) with rVSV_NJ_-Spike. 5-OP-RU or vehicle was added to each vaccine inoculum. The frequencies (left panels) and absolute numbers (right panels) of pulmonary (upper panels) and splenic (lower panels) MAIT cells were determined on day 7 post-secondary immunization. Data are shown as mean ± SEM. **, *** and **** denote differences with *p* ≤ 0.01, *p* ≤ 0.001 and *p* ≤ 0.0001, respectively, by one-way ANOVA followed by Tukey’s post-hoc Multiple Comparisons analyses.
(TIF)

S23 Fig Transcriptional assessment of pro/anti-survival gene expression by pulmonary MAIT cells after intraperitoneal administration of PR8 and/or 5-OP-RU. B6-MAIT^CAST^ mice were injected with PBS (n = 10), PR8 (n = 10), 5-OP-RU (n = 10), or PR8 plus 5-OP-RU (n = 5). Three days later, pooled pulmonary MAIT cells were evaluated by real-time PCR for their transcript levels of indicated molecules. Gene expression fold changes for each cohort relative to the control (PBS-injected) cohort were calculated as described in Materials and Methods.
(TIF)

S24 Fig PR8-specific IgM and IgG blood levels are comparable in MAIT cell-sufficient and -deficient mice. *Mr1*^+/+^ and *Mr1*^-/-^ B6-MAIT^CAST^ mice were bled either 10 (**A-B**) or 21 (**C-D**) days after they had been immunized with PR8 *i*.*p*. Total PR8-specific IgM (**A** and **C**) and IgG (**B** and **D**) titres were determined in serum samples by ELISA as described in Materials and Methods. Error bars represent SEM values (n = 3 mice/cohort).
(TIF)

S25 Fig 5-OP-RU does not raise PR8-specific IgM and IgG levels in the peripheral blood of MAIT cell-sufficient mice. B6-MAIT^CAST^ mice were bled 10 (**A-B**) or 21 (**C-D**) days after they had been injected *i*.*p*. with PR8 plus 5-OP-RU (or vehicle) as indicated. PR8-specific IgM (**A** and **C**) and IgG (**B** and **D**) titres were then determined in the sera of immunized mice by ELISA as detailed in Materials and Methods. Error bars represent SEM values (n = 6 mice/cohort).
(TIF)

S26 Fig Gene expression analysis of cytolytic effector molecules expressed by pulmonary MAIT cells following intraperitoneal injection of PR8 and/or 5-OP-RU. B6-MAIT^CAST^ mice were injected with PBS (n = 10), PR8 (n = 10), 5-OP-RU (n = 10), or PR8 plus 5-OP-RU (n = 5). After 3 days, pulmonary MAIT cells were purified, pooled and assessed by real-time PCR for their transcript levels of listed molecules. Gene expression fold changes were determined for each cohort compared with the PBS-injected (control) cohort as described in Materials and Methods.
(TIF)
