# Supplementary material for: Targeting the MR1-MAIT cell axis improves vaccine efficacy and affords protection against viral pathogens
Source: PLoS Pathog. 2023 Jun 29;19(6):e1011485. doi: 10.1371/journal.ppat.1011485 (PMC10337970; doi:10.1371/journal.ppat.1011485)
Supplement: S1 Table — (PDF) [file ppat.1011485.s001.pdf]

Supplementary Table 1. TaqMan-based quantitative PCR primer/probe sets used in this investigation

Target Gene	Assay Identifier
Actb	Mm00607939_s1
B2m	Mm00437762_m1
Bad	Mm00432042_m1
Bak1	Mm00432045_m1
Bax	Mm00432051_m1
Bcl2	Mm00477631_m1
Bcl6	Mm00477633_m1
Bid	Mm00432073_m1
Btla	Mm00616981_m1
Ccr3	Mm00515543_s1
Ccr4	Mm01963217_u1
Ccr6	Mm99999114_s1
Cd27	Mm01185212_g1
Cd28	Mm00483137_m1
Cd244	Mm00479575_m1
Cd40lg	Mm00441911_m1
Ctla4	Mm00486849_m1
Cxcr6	Mm02620517_s1
Fas	Mm01204974_m1
Fasl	Mm00438864_m1
Foxp3	Mm00475162_m1
Gapdh	Mm99999915_g1
Gata3	Mm00484683_m1
Gzma	Mm01304452_m1
Gzmb	Mm00442834_m1
Gzmk	Mm00492530_m1
Havcr2	Mm01294183_m1
Hmbs	Mm00660262_g1
Hprt1	Mm00446968_m1
Icos	Mm00497600_m1
Ifna1/Ifna5/Ifna6	Mm03030145_gH
Ifna2	Mm00833961_s1
Ifna4	Mm00833969_s1
Ifnb1	Mm00439552_s1
Ifng	Mm01168134_m1
Ifnar1	Mm00439544_m1
Ifngr1	Mm00599890_m1
Il2	Mm00434256_m1

Il4	Mm00445259_m1
Il5	Mm00439646_m1
Il6	Mm00446190_m1
Il9	Mm00434305_m1
Il10	Mm00439614_m1
Il13	Mm00434204_m1
Il17a	Mm00439618_m1
Il17b	Mm01258783_m1
Il17c	Mm00521397_m1
Il17f	Mm00521423_m1
Il22	Mm01226722_g1
Il2rb	Mm00434268_m1
Il4ra	Mm00439634_m1
Il12rb1	Mm00434189_m1
Il13ra1	*Mm00446726_m1*
Il15ra	*Mm04336046_m1*
Il18r1	Mm00515178_m1
Il23r	Mm00519943_m1
Il27ra	Mm00497259_m1
Ipo8	Mm01255158_m1
Lag3	Mm00493071_m1
Mapk1	Mm00442479_m1
Pdcd1	Mm01285676_m1
Pdcd1lg1	Mm03048248_m1
Pdcd1lg2	Hs00228839_m1
Pgk1	Mm00435617_m1
Prf1	Mm00812512_m1
Rora	Mm00443103_m1
Rorc	Mm01261022_m1
Rplp2	Mm00782638_s1
Slamf7	Mm00513808_m1
Tbp	Mm00446973_m1
Tbx21	Mm00450960_m1
Tfrc	Mm00441941_m1
Tgfb1	Mm01178820_m1
Tlr3	Mm01207404_m1
Tlr4	Mm00445273_m1
Tlr7	Mm04933178_g1
Tlr9	Mm00446193_m1
Tnf	Mm00443258_m1
Tnfsf4	Mm00437214_m1

Tnfsf8	Mm00437153_m1
Tnfsf9	Mm00437155_m1
Tnfsf10	Mm01283606_m1
Tnfsf11	Mm00441906_m1
Tnfsf12	Mm02583406_s1
Tnfsf13	Mm03809849_s1
Tnfsf13b	Mm00446347_m1
Tnfsf14	Mm00444567_m1
Tnfsf15	Mm00770031_m1
Tnfsf18	Mm00839222_m1
Tyk2	Mm00444469_m1
Ubc	Mm01201237_m1
Vsir	Mm00472312_m1
Xiap	Mm01311594_mH
Zbtb16	Mm01176868_m1
