# Supplementary material for: Targeting the MR1-MAIT cell axis improves vaccine efficacy and affords protection against viral pathogens
Source: PLoS Pathog. 2023 Jun 29;19(6):e1011485. doi: 10.1371/journal.ppat.1011485 (PMC10337970; doi:10.1371/journal.ppat.1011485)
Supplement: S2 Table — (PDF) [file ppat.1011485.s002.pdf]

S2 Table. List of Antibodies, Tetramers and other reagents

Antibody	Clone/Catalogue Number	Company
Alexa Fluor 700-conjugated anti-human CD3	UCHT1	Thermo Fisher Scientific
Anti-mouse CD3 monoclonal antibody	17A2	Bio X Cell
APC-conjugated anti-mouse CD8α	53-6.7	Thermo Fisher Scientific
Anti-mouse CD28 monoclonal antibody	37.51	Bio X Cell
PE-conjugated anti-human CD38	HIT2	Thermo Fisher Scientific
Alexa Fluor 700-conjugated anti-mouse CD45	30-F11	Thermo Fisher Scientific
Alexa Fluor 700-conjugated anti-mouse/human CD45R (B220)	RA3-6B2	Thermo Fisher Scientific
APC-conjugated anti-mouse/human CD45R (B220)	RA3-6B2	Thermo Fisher Scientific
PE-Cyanine7-conjugated anti-human CD69	FN50	Thermo Fisher Scientific
PerCP-Cyanine5.5-conjugated anti-mouse CD69	H1.2F3	Thermo Fisher Scientific
PE-conjugated anti-mouse CD103	2E7	Thermo Fisher Scientific
PE-conjugated anti-mouse CD122	TM-b1	Thermo Fisher Scientific
PE-eFluor610-conjugated anti-mouse GATA3	TWAJ	Thermo Fisher Scientific
APC-eFluor780-conjugated anti-human HLA-DR	LN3	Thermo Fisher Scientific
APC-conjugated anti-mouse IFN-γ	XMG1.2	Thermo Fisher Scientific
FITC-conjugated anti-mouse IFN-γ	XMG1.2	Thermo Fisher Scientific
Anti-IFNAR-1 monoclonal antibody	MAR1-5A3	Bio X Cell
Goat anti-mouse IgG (H+L) Secondary Antibody [HRP]	NBP1-75130	Novus Biologicals
Goat anti-mouse IgM (H+L) Secondary Antibody [HRP]	NBP1-75207	Novus Biologicals
PE-eFluor 610-conjugated anti-mouse IL-17A	eBio17B7	Thermo Fisher Scientific
PE-eFluor610-conjugated anti-mouse Ki-67	SolA15	Thermo Fisher Scientific
APC-conjugated anti-mouse/human RORγt	AFKJS-9	Thermo Fisher Scientific
APC-conjugated anti-mouse S1P1/EDG-1	713412	R&D Systems
PerCP-Cyanine5.5-conjugated anti-human/mouse/rhesus monkey T-Bet	eBio 4B10	Thermo Fisher Scientific
Alexa Fluor 700-conjugated anti-mouse TCRβ	H57-597	Thermo Fisher Scientific
PE-Cyanine7-conjugated anti-mouse TCRβ	H57-597	Thermo Fisher Scientific
**Isotype Controls**		
PerCP-Cyanine5.5-conjugated Armenian hamster IgG isotype control	eBio299Arm	Thermo Fisher Scientific
PE-conjugated mouse IgG1κ isotype control	P3.6.2.8.1	Thermo Fisher Scientific
PE-Cyanine7-conjugated mouse IgG1κ isotype control	P3.6.2.8.1	Thermo Fisher Scientific
Mouse IgG1κ isotype control	MOPC-21	Bio X Cell
PerCP-Cyanine5.5-conjugated mouse IgG1κ isotype control	P3.6.2.8.1	Thermo Fisher Scientific
APC-conjugated rat IgG1κ isotype control	eBRG1	Thermo Fisher Scientific
APC-conjugated rat IgG2a isotype control	54447	R&D Systems
APC-conjugated rat IgG2aκ isotype control	eBR2a	Thermo Fisher Scientific
PE-eFluor610-conjugated rat IgG2aκ isotype control	eBR2a	Thermo Fisher Scientific
APC-eFluor780-conjugated mouse IgG2bκ isotype control	eBMG2b	Thermo Fisher Scientific
PE-eFluor610-conjugated rat IgG2bκ isotype control	eB149/10H5	Thermo Fisher Scientific
**Tetramers**		
APC-conjugated 5-OP-RU-loaded mouse MR1 tetramer		
PE-conjugated 5-OP-RU-loaded mouse MR1 tetramer		
APC-conjugated 6-FP-loaded mouse MR1 tetramer		
PE-conjugated 6-FP-loaded mouse MR1 tetramer	N/A	NIH Tetramer Core Facility
APC-conjugated 5-OP-RU-loaded human MR1 tetramer		
APC-conjugated 6-FP-loaded human MR1 tetramer		
Alexa Fluor488-conjugted $NP_{147\text{-}155}$ peptide-loaded H-$2K^d$ tetramer		

APC-conjugated PBS-57-loaded mouse CD1d tetramer<br>APC-conjugated empty mouse CD1d tetramer	N/A	NIH Tetramer Core Facility

**Other Chemicals and Reagents Used**	Company/Source
4-(2-hydroxyethyl)-1-piperazineethanesulfonic acid (HEPES)	Gibco
5-amino-6-D-ribitylaminouracil (5-ARU)	Dr. Olivier Lantz
7-aminoactinomycin D (7-AAD) viability dye	Thermo Fisher Scientific
Anti-PE MicroBeads UltraPure	Miltenyi Biotec
Bovine Serum Albumin (BSA)	Sigma
Brefeldin A	Sigma-Aldrich
Carboxyfluorescein diacetate succinimidyl ester (CFDA-SE)	Invitrogen
CellTrace Far Red dye	Thermo Fisher Scientific
Collagenase type IV	Sigma
CTS Immune Cell Serum Replacement	Thermo Fisher Scientific
Dimethyl sulfoxide (DMSO)	Sigma
DNase I	Sigma
eBioscience Ready-SET-Go! Mouse IFN-γ ELISA Kit	Thermo Fisher Scientific
eBioscience Ready-SET-Go! Mouse IL-17A ELISA Kit	Thermo Fisher Scientific
eBioscience Ready-SET-Go! Mouse IL-4 ELISA Kit	Thermo Fisher Scientific
eBioscience Ready-SET-Go! Mouse TNF-α ELISA Kit	Thermo Fisher Scientific
Ethylenediaminetetraacetic acid (EDTA)	Invitrogen
Fetal bovine serum (FBS)	Gibco
Ficoll-Paque PLUS	Cytiva
FluMist Quadrivalent (2021-2022)	AstraZeneca Canada Inc.
Foxp3/Transcription Factor Staining Buffer Set	Thermo Fisher Scientific
FTY720	Sigma
GlutaMAX	Gibco
Imiquimod	InvivoGen
ImmunoCult T Cell Expansion Medium	STEMCELLTechnologies
Intracellular Fixation & Permeabilization Buffer Set	Thermo Fisher Scientific
Ionomycin	Sigma-Aldrich
Isoflurane	Baxter
MEM nonessential amino acids	Gibco
Methylglyoxal	Sigma-Aldrich
Normocin	InvivoGen
N-tosyl-L-phenylalanine chloromethyl ketone-treated trypsin	Sigma
Opti-MEM Reduced Serum Medium	Thermo Fisher Scientific
Penicillin/Streptomycin	Gibco
Percoll PLUS	GE Healthcare
Phenylmethylsulfonyl fluoride	Thermo Scientific
Phorbol 12-myristate 13-acetate (PMA)	Sigma-Aldrich
Phosphate-buffered saline (PBS)	Sigma
PicoPure RNA Isolation Kit	Thermo Fisher Scientific
Polyinosinic-polycytidylic acid [poly (I:C)]	InvivoGen
Recombinant mouse IL-2	R&D Systems
Recombinant mouse IL-12p70	Peprotech
Recombinant mouse IL-18	R&D Systems
Recombinant mouse Interferon-α1 (rmIFNα1)	R&D Systems
Recombinant mouse Interferon-β (rmIFNβ)	R&D Systems

RPMI-1640	Gibco
Sodium pyruvate	Gibco
SuperScript IV VILO Master Mix with ezDNase Enzyme	Thermo Fisher Scientific
TaqMan Fast Advanced Master Mix	Thermo Fisher Scientific
Trypan blue	Gibco
