# Supplementary figures and images for: Targeting the MR1-MAIT cell axis improves vaccine efficacy and affords protection against viral pathogens
Source: PLoS Pathog. 2023 Jun 29;19(6):e1011485. doi: 10.1371/journal.ppat.1011485 (PMC10337970; doi:10.1371/journal.ppat.1011485)

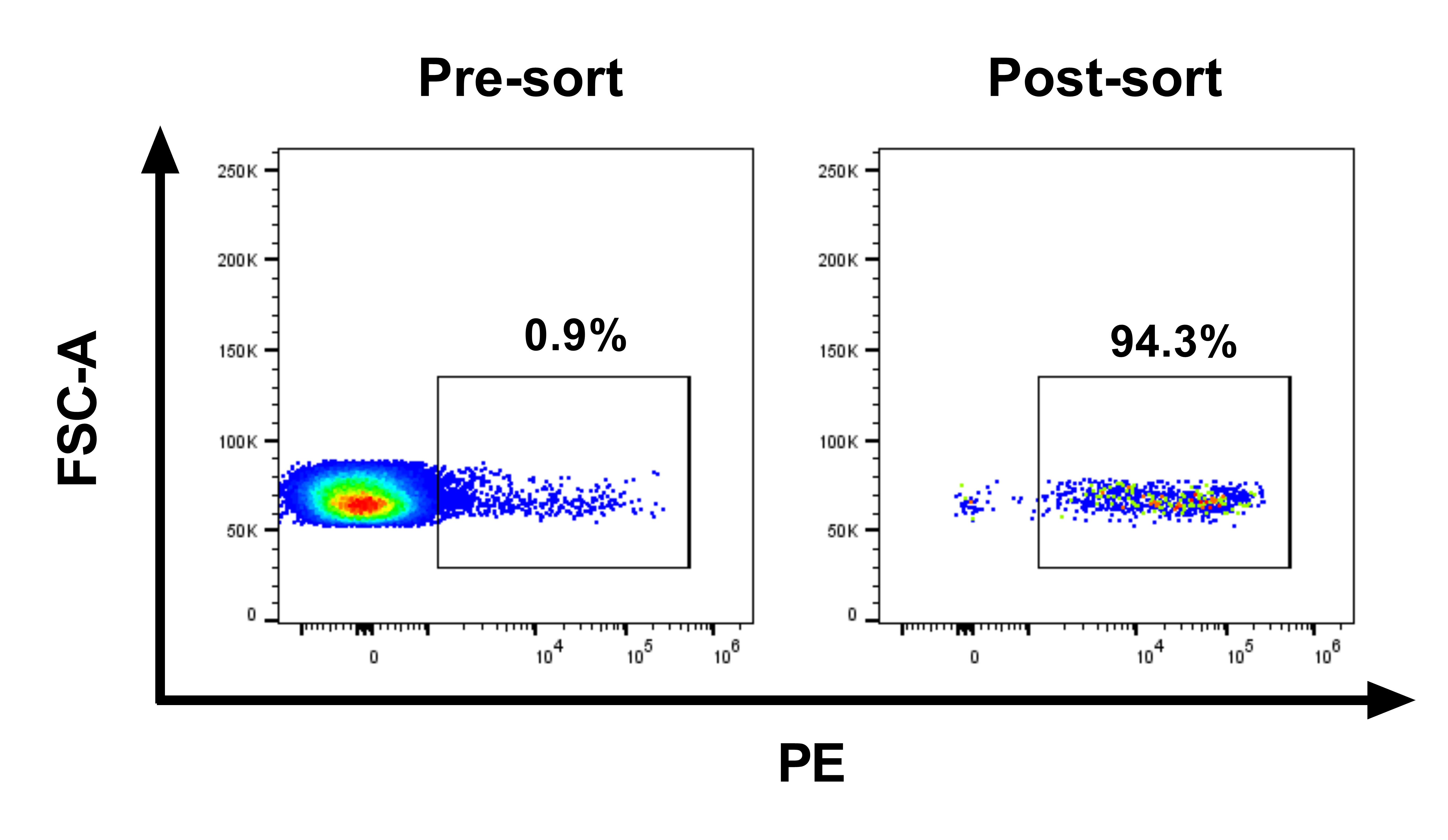

Supplement: S1 Fig — Five B6-MAITCAST mice were injected i.p. with a combination of PR8 and 5-OP-RU. Three days later, non-parenchymal lung mononuclear cells (LMNCs) were isolated, pooled and stained with phycoerythrin (PE)-conjugated, 5-OP-RU-loaded mouse MR1 tetramers. MAIT cells were then magnetically purified using anti-PE microbeads as detailed in Materials and Methods. MAIT cell percentages among bulk LMNCs (pre-sort) and after two rounds of column purification (post-sort) are shown. (TIF) [file ppat.1011485.s004.tif]

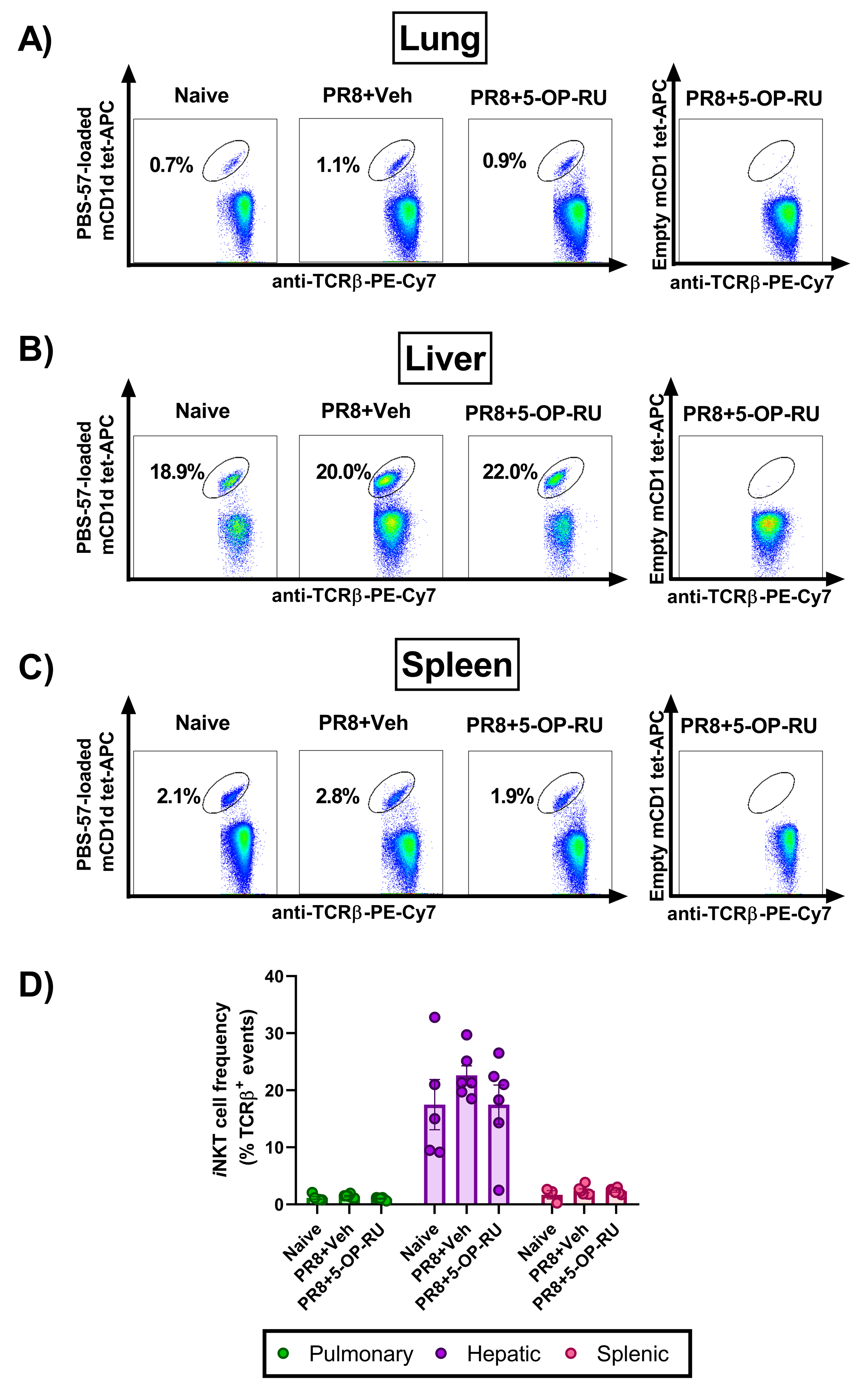

Supplement: S3 Fig — Naïve B6-MAITCAST mice (n = 5) and mice that had received PR8 plus 5-OP-RU (or vehicle) three days earlier (n = 6) were sacrificed for their lungs (A and D), liver (B and D) and spleen (C-D) in which TCRβ+ PBS-57-loaded mCD1d tetramer+ iNKT cells were enumerated. Empty mCD1d tetramers were used in parallel to draw cytofluorimetric gates (A-C). Representative plots (A-C) and summary data with mean ± SEM values (D) are shown. Group comparisons were made using the Kruskal-Wallis test followed by the Dunn’s post-hoc analysis. (TIF) [file ppat.1011485.s006.tif]

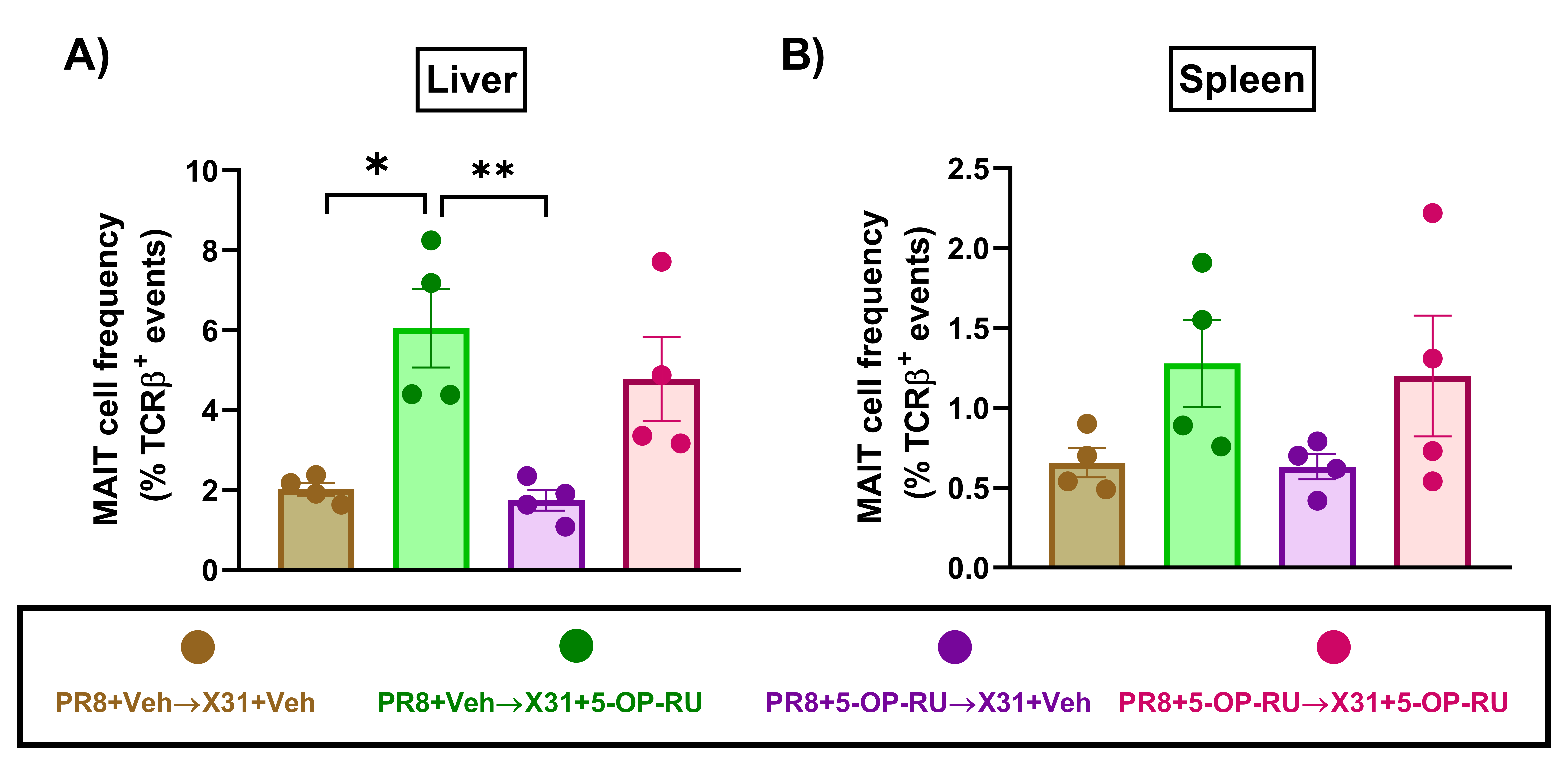

Supplement: S5 Fig — B6-MAITCAST mice were primed i.p. with PR8 (H1N1) and boosted i.p., four weeks later, with X31 (H3N2). 5-OP-RU (or vehicle) was co-administered in both phases. Three days after secondary immunization, hepatic (A) and splenic (B) MAIT cells were enumerated by flow cytometry. Each circle represents an individual mouse, and data are shown as mean ± SEM. * and ** denotes a significant difference with p ≤ 0.05 and p ≤ 0.01, respectively, using one-way ANOVA followed by the Tukey’s Multiple Comparisons test. (TIF) [file ppat.1011485.s008.tif]

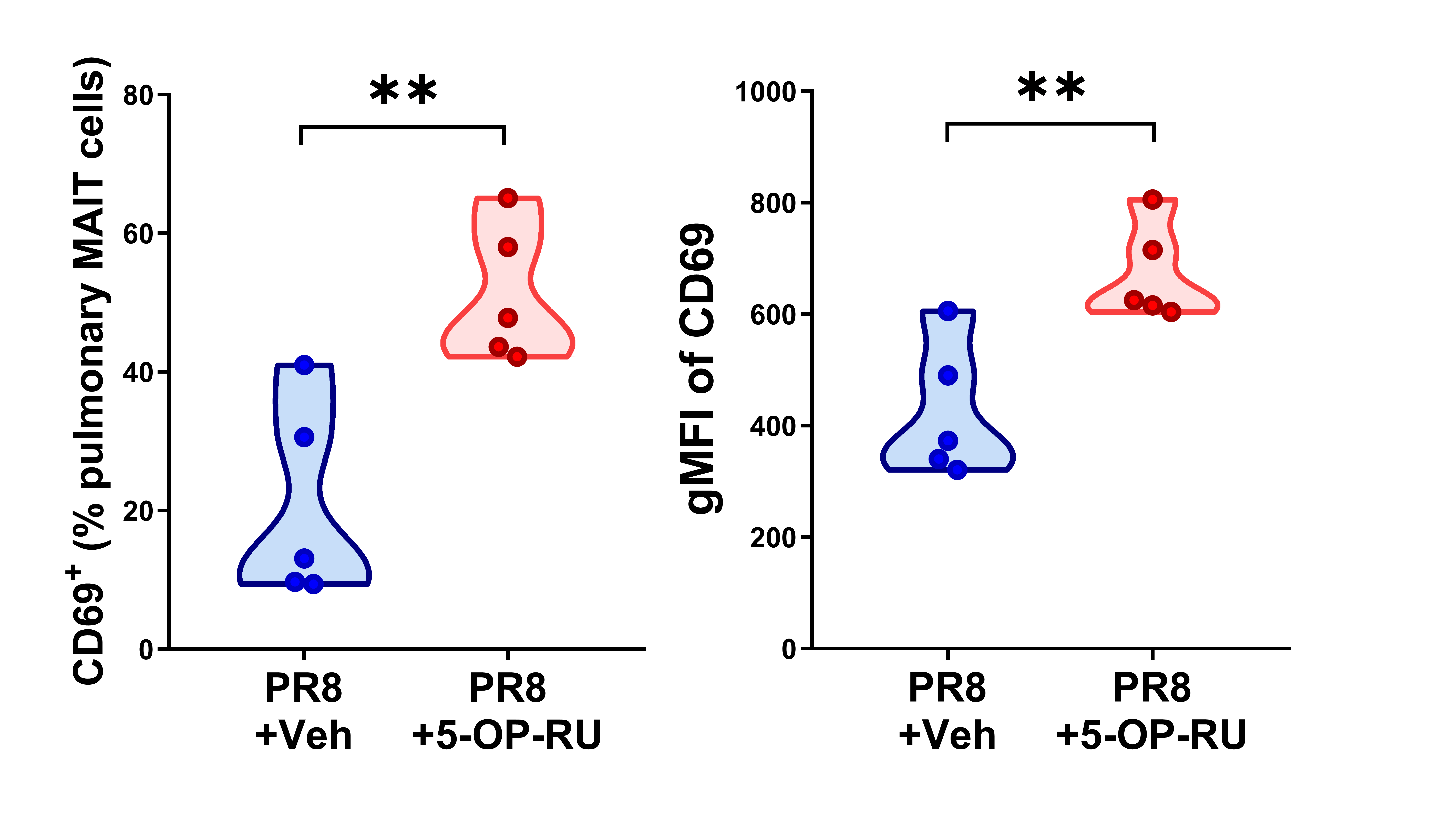

Supplement: S6 Fig — B6-MAITCAST mice (n = 5/group) were injected i.p. with PR8 plus 5-OP-RU or vehicle. Three days later, CD69+ MAIT cell frequencies in the lungs and the geometric mean fluorescence intensity (gMFI) of CD69 staining were determined by flow cytometry. ** denotes significant differences with p ≤ 0.01 by unpaired t-tests. (TIF) [file ppat.1011485.s009.tif]

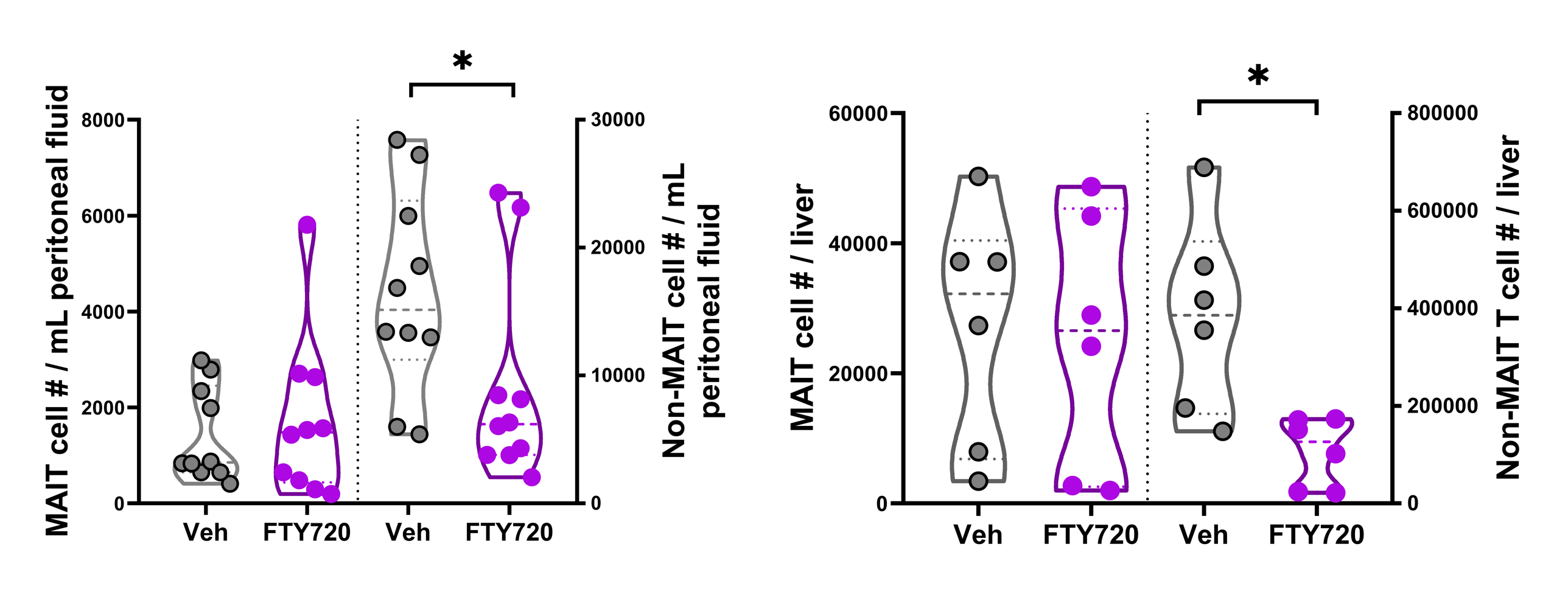

Supplement: S7 Fig — B6-MAITCAST mice were injected i.p. with FTY720 (or vehicle) 2 hours before and 22 hours after immunization with 5-OP-RU-adjuvanted PR8, followed by TCRβ+ MR1 tetramer+ MAIT and TCRβ+ MR1 tetramer- non-MAIT T cell enumeration in the peritoneal cavity (left panel) and liver (right panel) on day 3 post-immunization. Each circle represents an individual mouse. Data were pooled from four (left panel) or three (right panel) independent experiments yielding similar results, and * denotes p ≤ 0.05 by Mann-Whitney U test. (TIF) [file ppat.1011485.s010.tif]

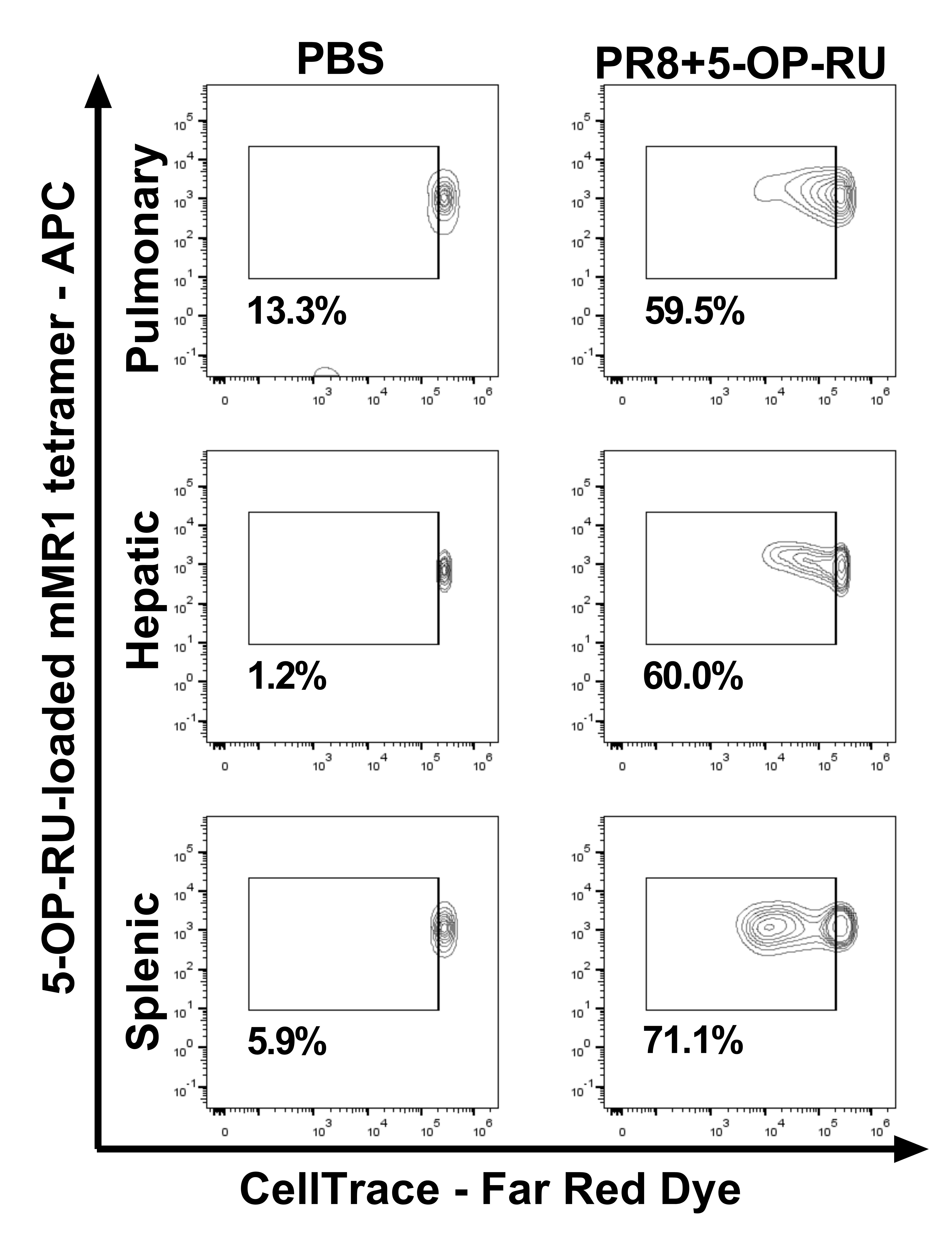

Supplement: S8 Fig — MAIT cells were magnetically purified out of pooled non-parenchymal pulmonary and hepatic mononuclear cells from naïve B6-MAITCAST mice, expanded as described in Materials and Methods, labeled with CellTrace Far Red dye, and adoptively transferred i.v. into B6-MAITCAST recipients. Twenty-four hours later, animals were injected i.p. with PBS or with a combination of PR8 and 5-OP-RU. After 3 days, mice were sacrificed for their lungs, liver and spleen in which CellTrace dye dilution by MAIT cells was examined by flow cytometry. The percentages of proliferated MAIT cells are shown for each organ. (TIF) [file ppat.1011485.s011.tif]

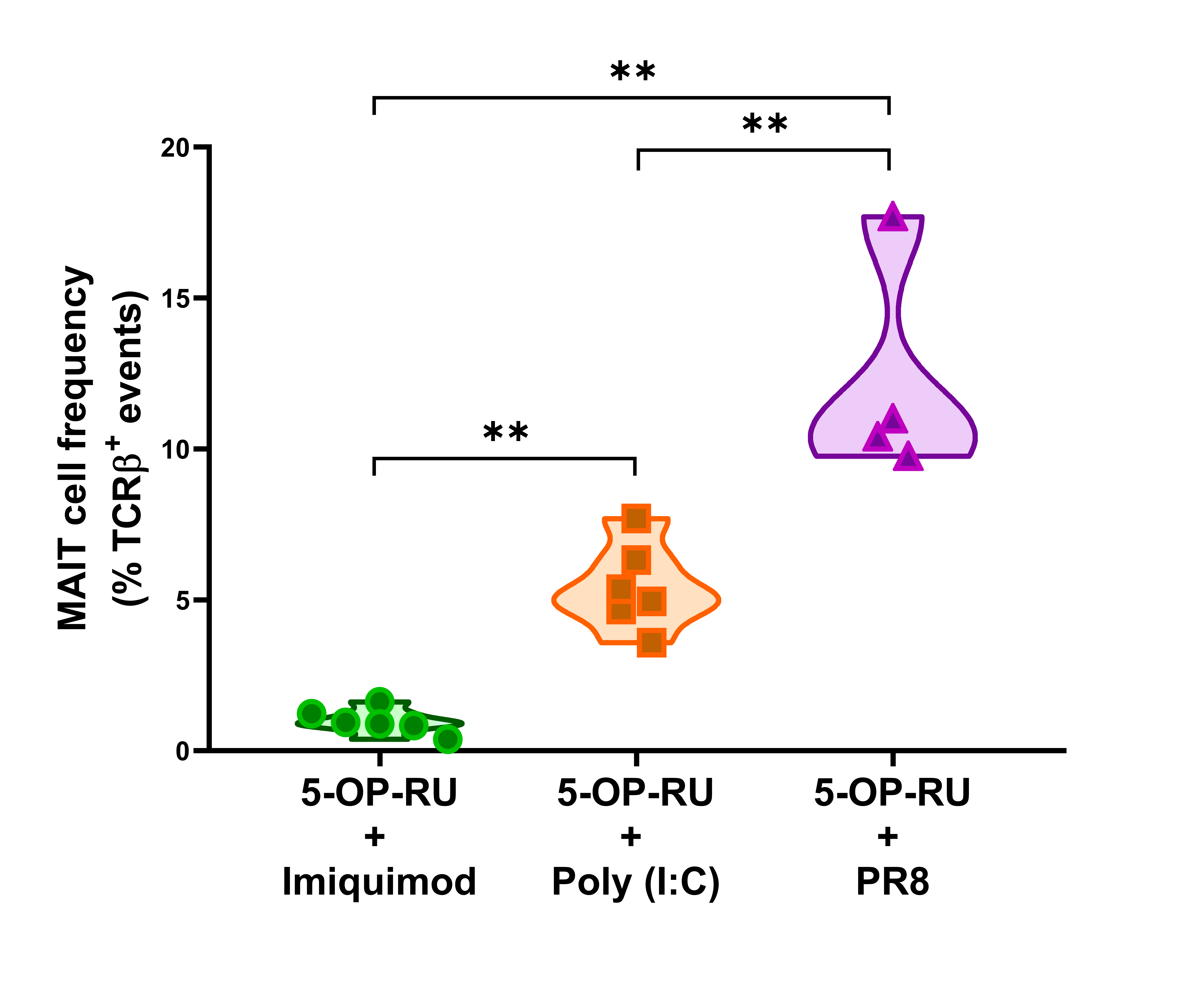

Supplement: S9 Fig — B6-MAITCAST mice were injected i.p. with 5-OP-RU in combination with PR8, poly (I:C) (50 μg/mouse) or imiquimod (50 μg/mouse). Three days later, peritoneal MAIT cell frequencies were determined by flow cytometry. Each symbol represents an individual animal. ** denotes significant differences with p ≤ 0.01 by the Mann-Whitney U test. (TIF) [file ppat.1011485.s012.tif]

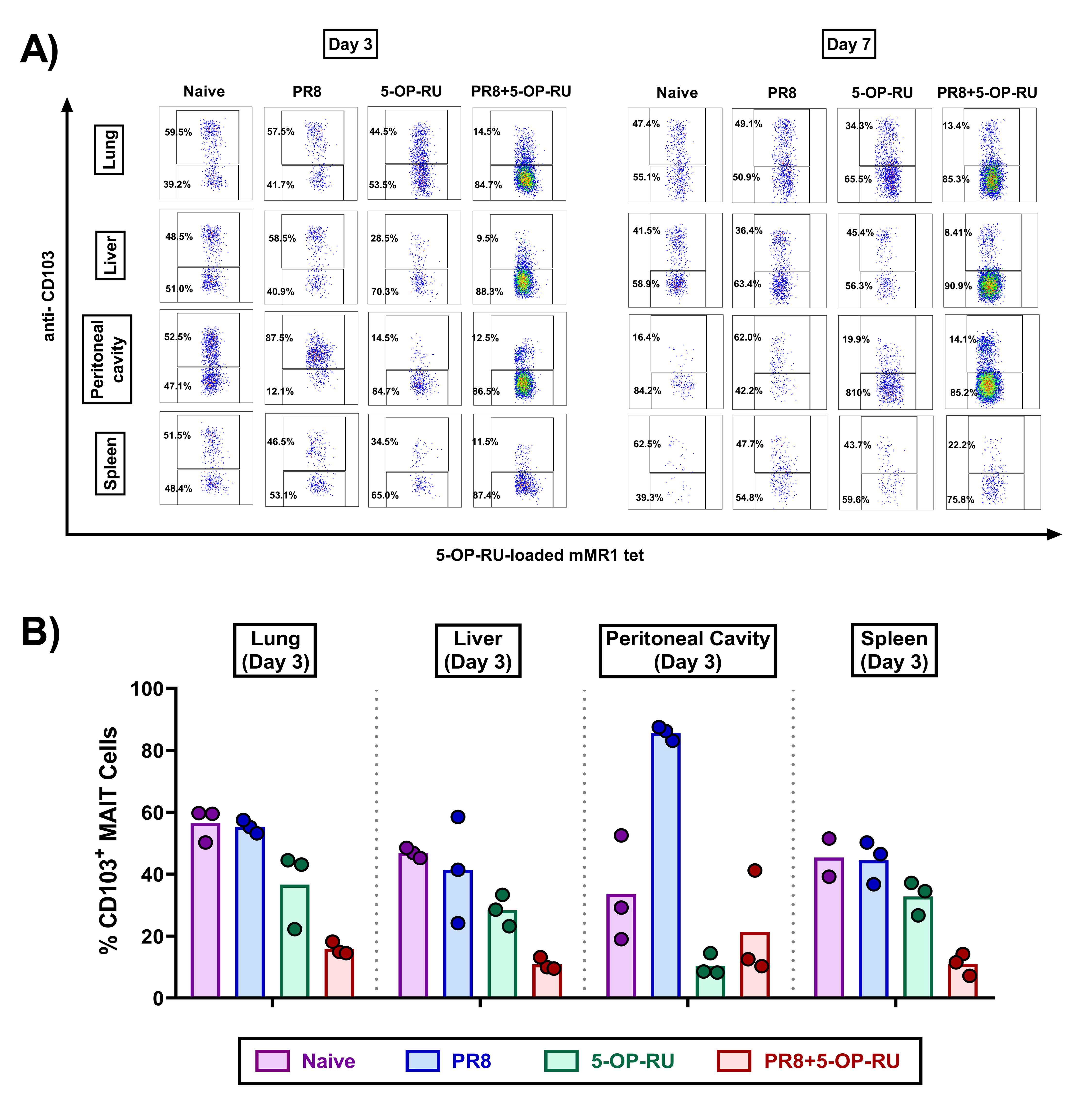

Supplement: S11 Fig — Naïve B6-MAITCAST mice and those receiving PR8, 5-OP-RU, or both, 3 or 7 days earlier were sacrificed before pulmonary, hepatic, peritoneal and splenic MAIT cells were assessed by flow cytometry for CD103 expression. Representative plots after gating on TCRβ+ cells (A) and summary data (B) are provided. (TIF) [file ppat.1011485.s014.tif]

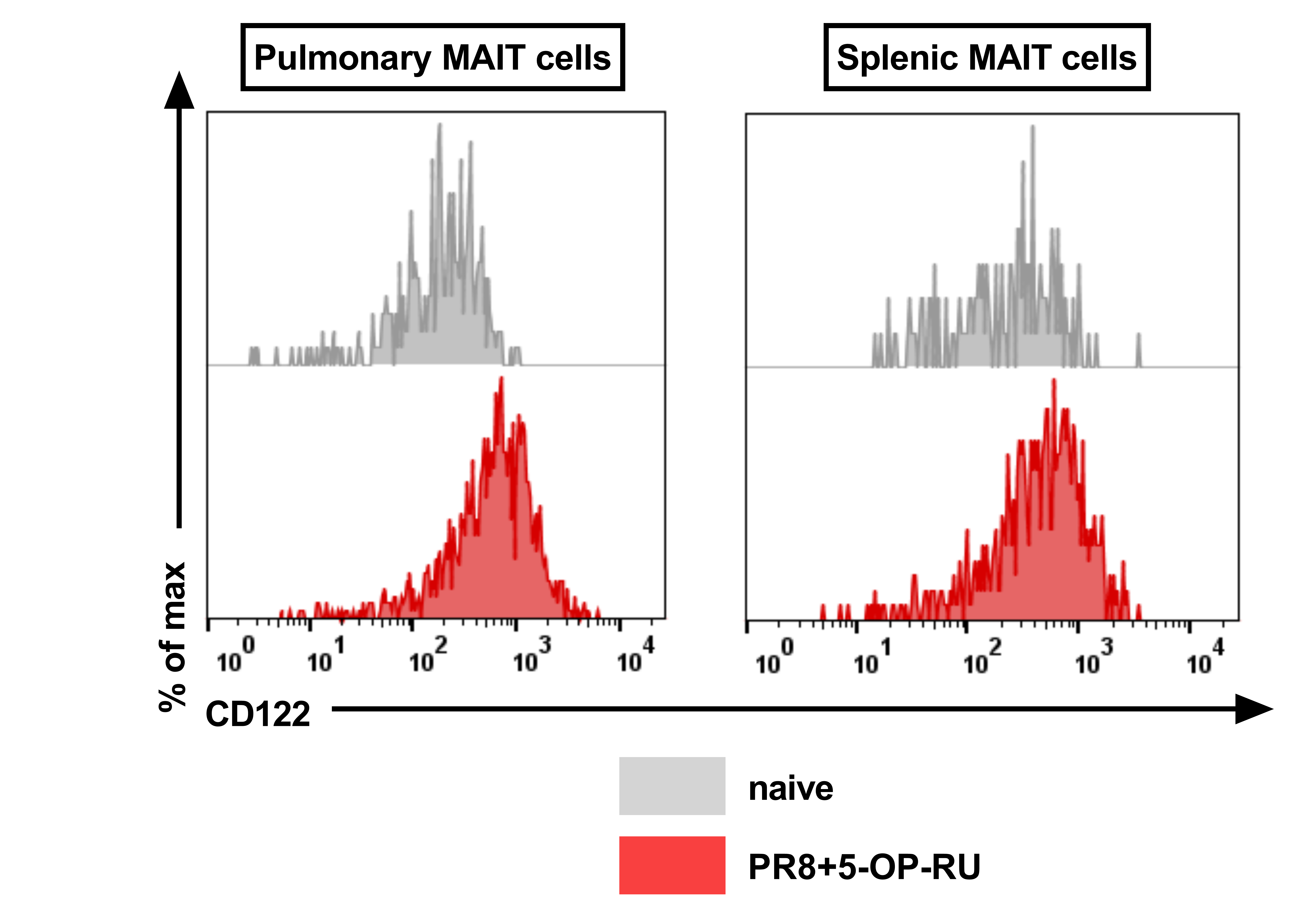

Supplement: S12 Fig — A naïve B6-MAITCAST mouse and a mouse that had received PR8 plus 5-OP-RU three days earlier were sacrificed, and pulmonary and splenic MAIT cells were examined cytofluorimetrically for CD122 expression. Histograms corresponding to naïve mouse values are overlaid with those from the immunized animal. (TIF) [file ppat.1011485.s015.tif]

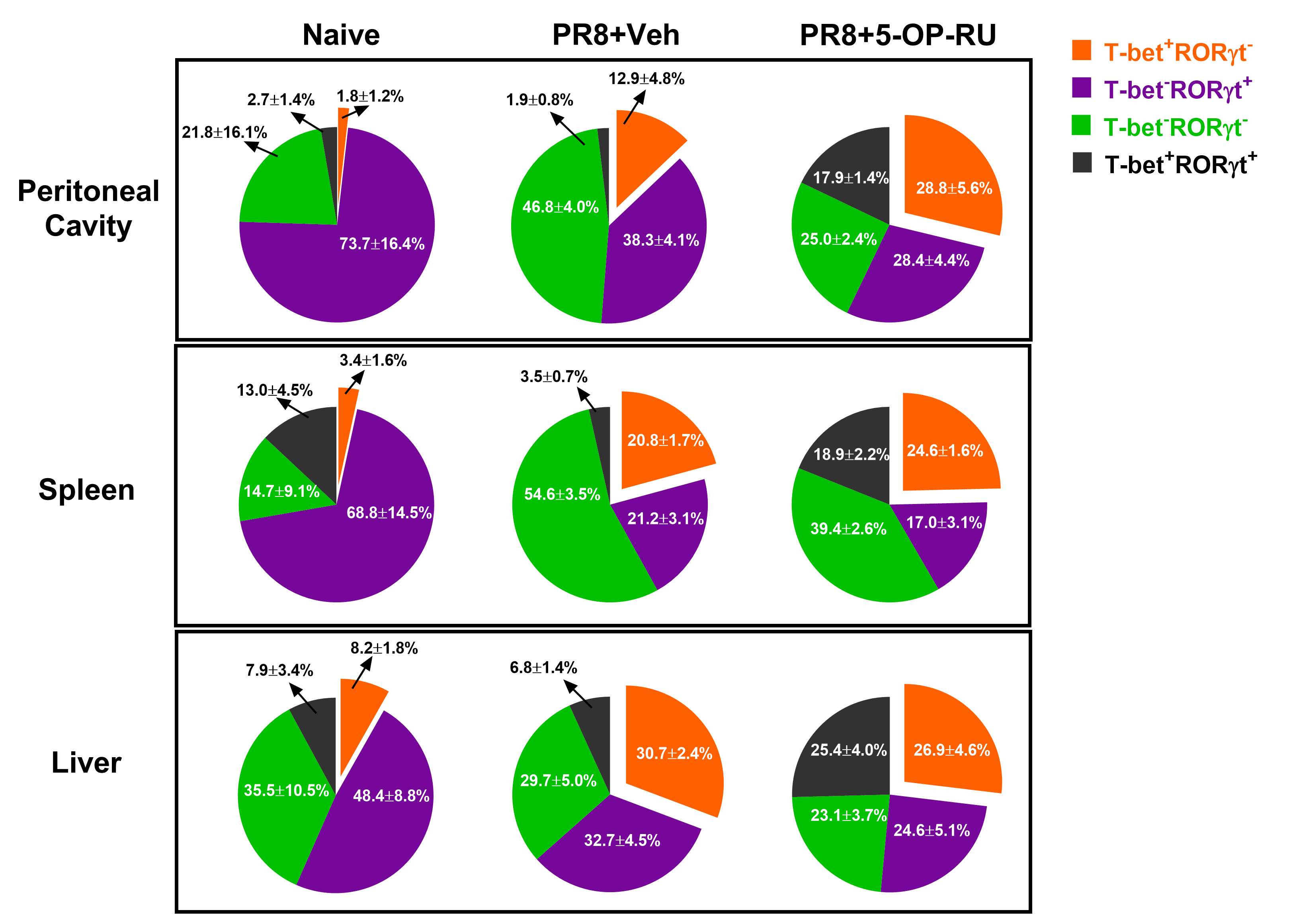

Supplement: S16 Fig — Naïve B6-MAITCAST mice (n = 5) and animals that had been inoculated 3 days earlier with PR8 plus 5-OP-RU (or vehicle) (n = 6/group) were sacrificed. Peritoneal, splenic and hepatic MAIT cells were then stained for intracellular T-bet and RORγt. Pie charts visualize the frequencies (± SEM) of T-bet+RORγt-, T-bet-RORγt+, T-bet+RORγt+ and T-bet-RORγt- MAIT cell subsets. (TIF) [file ppat.1011485.s019.tif]

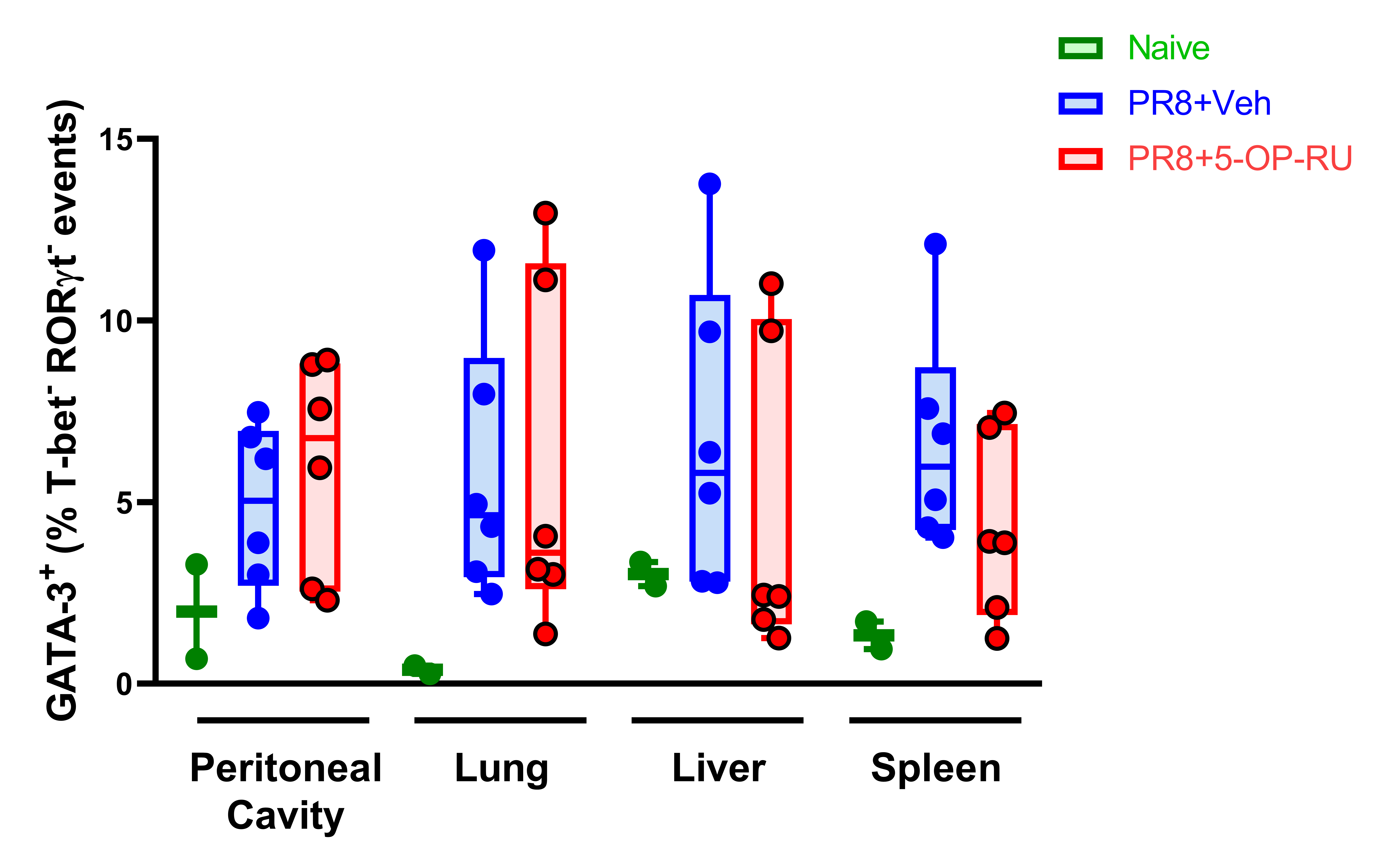

Supplement: S17 Fig — Two naïve B6-MAITCAST mice and 12 animals that had received an i.p. injection of PR8 plus 5-OP-RU (or vehicle) (n = 6/group) three days earlier were sacrificed. Peritoneal, pulmonary, hepatic and splenic MAIT cells were interrogated by flow cytometry for their intracellular T-bet, RORγt and GATA-3 levels. Box-and-Whisker plots demonstrate GATA-3+ cell percentages among T-bet-RORγt- MAIT cells. (TIF) [file ppat.1011485.s020.tif]

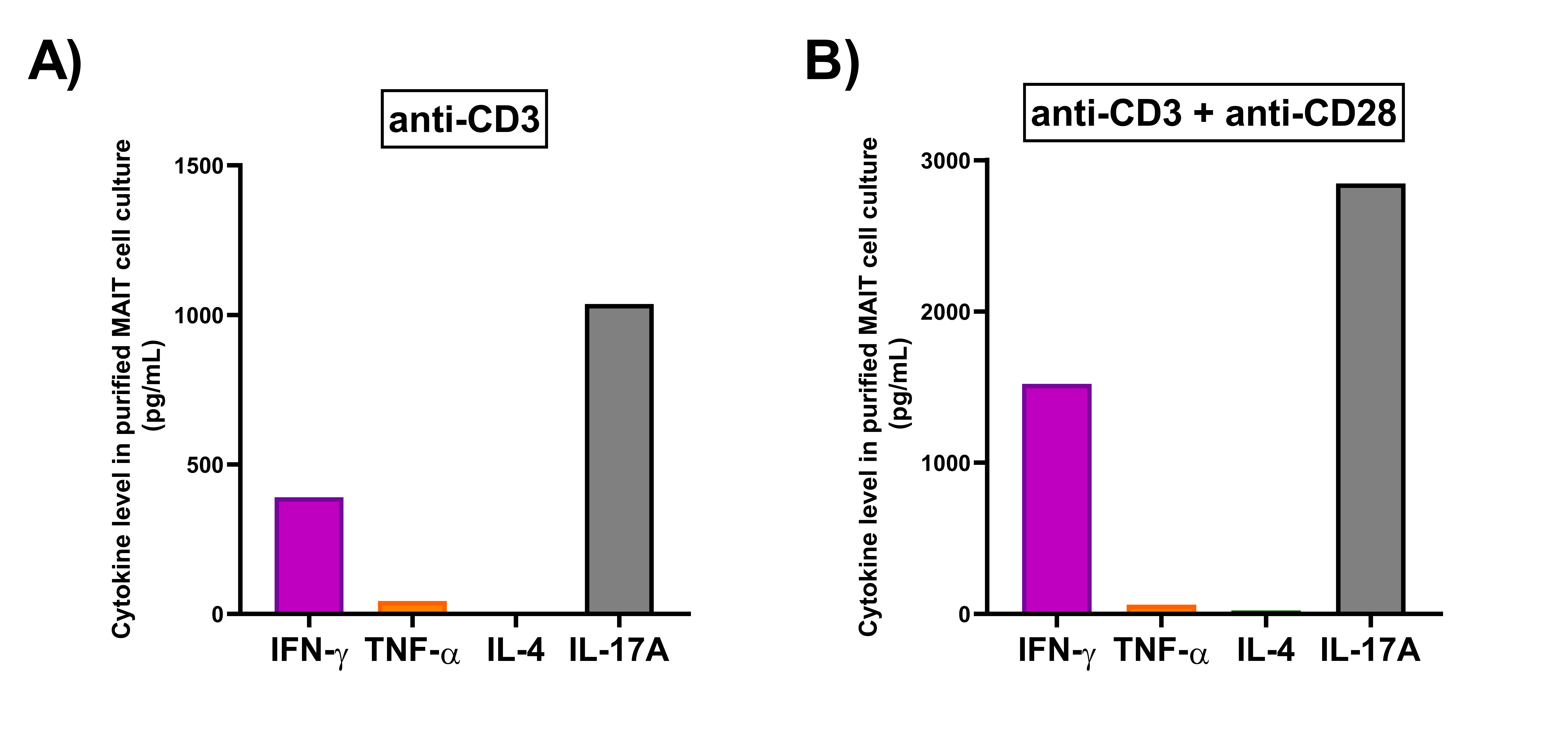

Supplement: S19 Fig — Purified pulmonary MAIT cells were pooled from 10 B6-MAITCAST mice that had been inoculated i.p. with PR8 and 5-OP-RU three days earlier. Cells were stimulated with plate-coated anti-CD3 in the absence (A) or presence (B) of soluble anti-CD28 as described in Materials and Methods. Eighteen hours later, indicated cytokines were measured in culture supernatants by ELISA. (TIF) [file ppat.1011485.s022.tif]

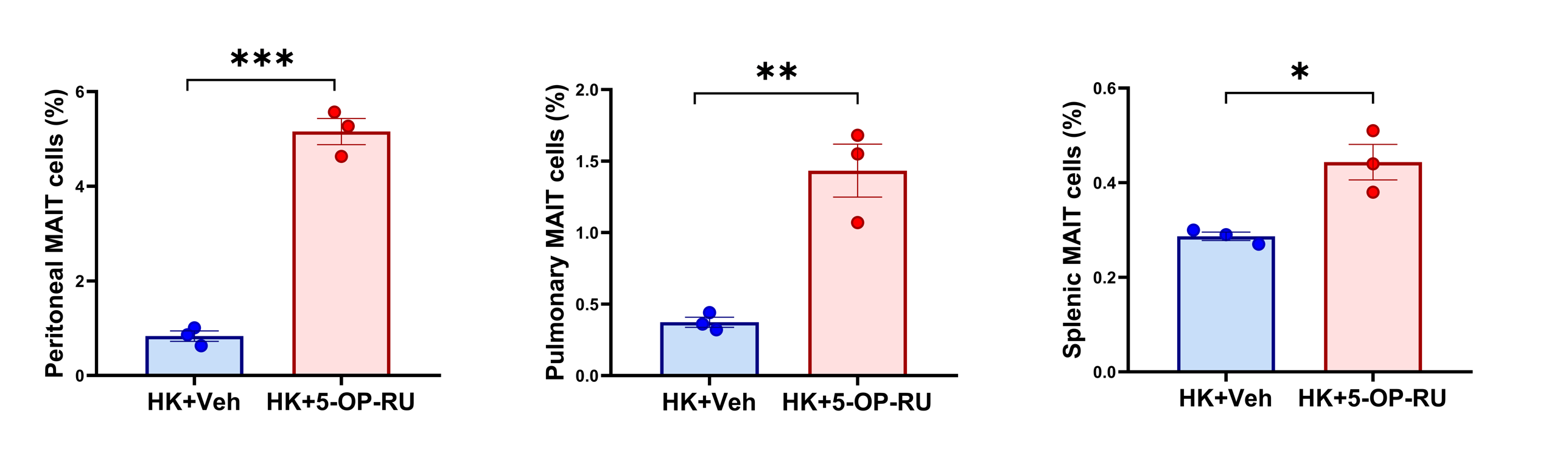

Supplement: S20 Fig — B6 mice (n = 3/group) were injected i.p. with the A/Hong Kong/1/1968 (HK) strain of IAV plus 5-OP-RU or vehicle. Three days later, peritoneal (left panel), pulmonary (middle panel) and splenic (right panel) MAIT cells were enumerated by flow cytometry. Unpaired t-tests were used for statistical comparisons. *, ** and *** indicate differences with p ≤ 0.05, p ≤ 0.01 and p ≤ 0.001, respectively. (TIF) [file ppat.1011485.s023.tif]

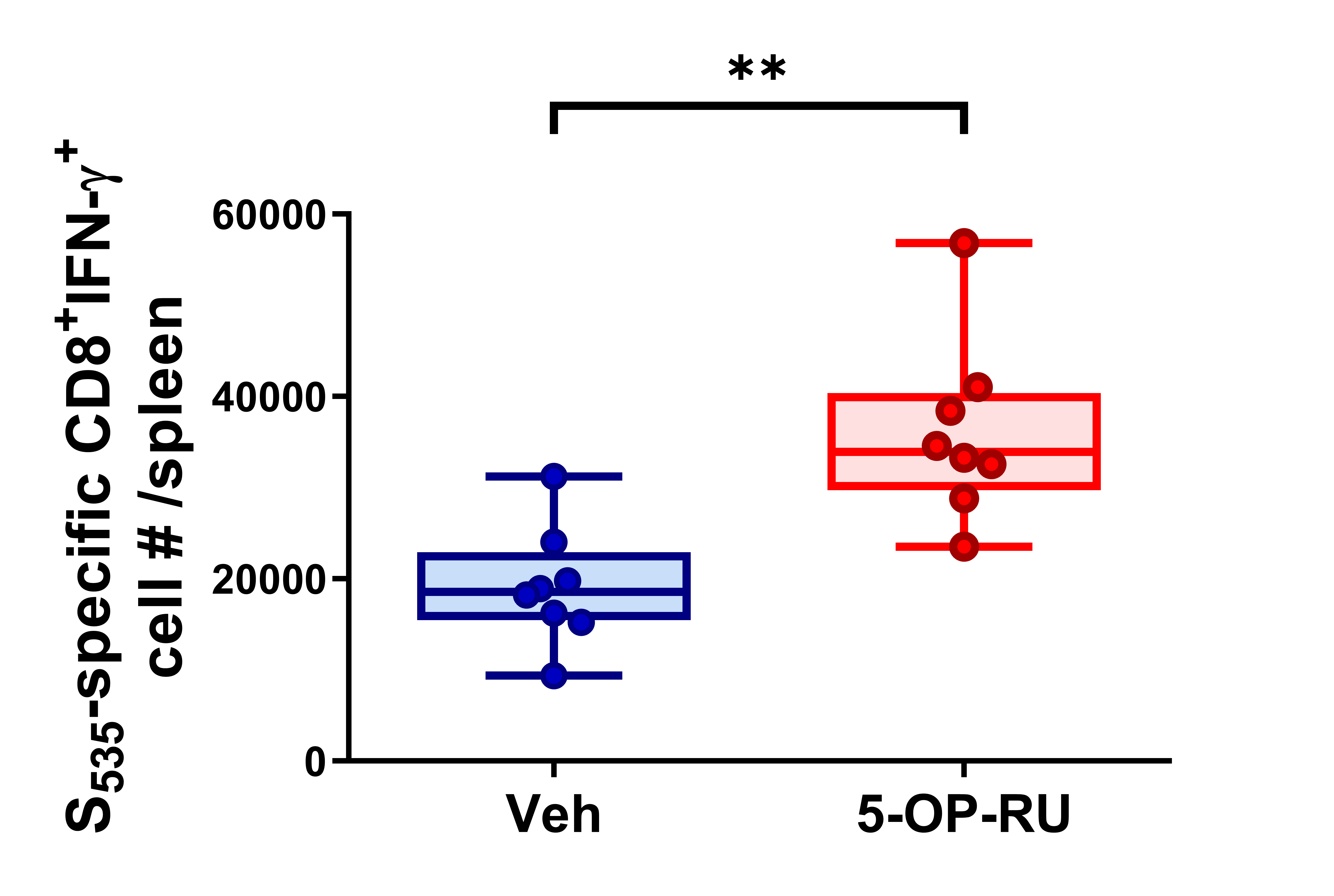

Supplement: S21 Fig — BALB/c mice were injected i.m. with rVSVInd expressing the SARS-CoV-2 Spike (S) gene plus 5-OP-RU (or vehicle). Seven days later, S535-specific CD8+ T cells were enumerated by intracellular cytokine staining for IFN-γ. Each circle represents an individual mouse, and ** denotes p ≤ 0.01 by unpaired t-test. (TIF) [file ppat.1011485.s024.tif]

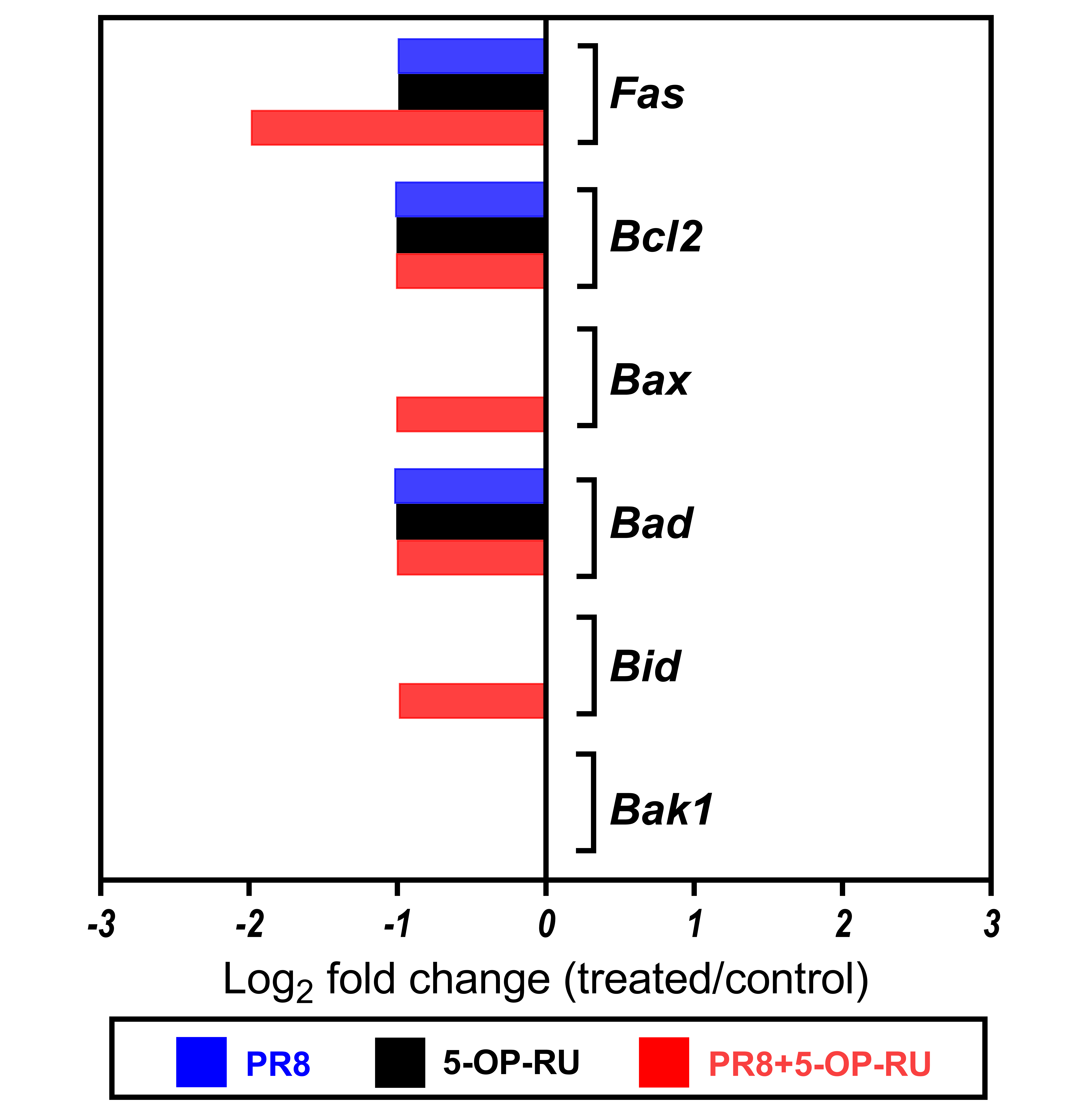

Supplement: S23 Fig — B6-MAITCAST mice were injected with PBS (n = 10), PR8 (n = 10), 5-OP-RU (n = 10), or PR8 plus 5-OP-RU (n = 5). Three days later, pooled pulmonary MAIT cells were evaluated by real-time PCR for their transcript levels of indicated molecules. Gene expression fold changes for each cohort relative to the control (PBS-injected) cohort were calculated as described in Materials and Methods. (TIF) [file ppat.1011485.s026.tif]

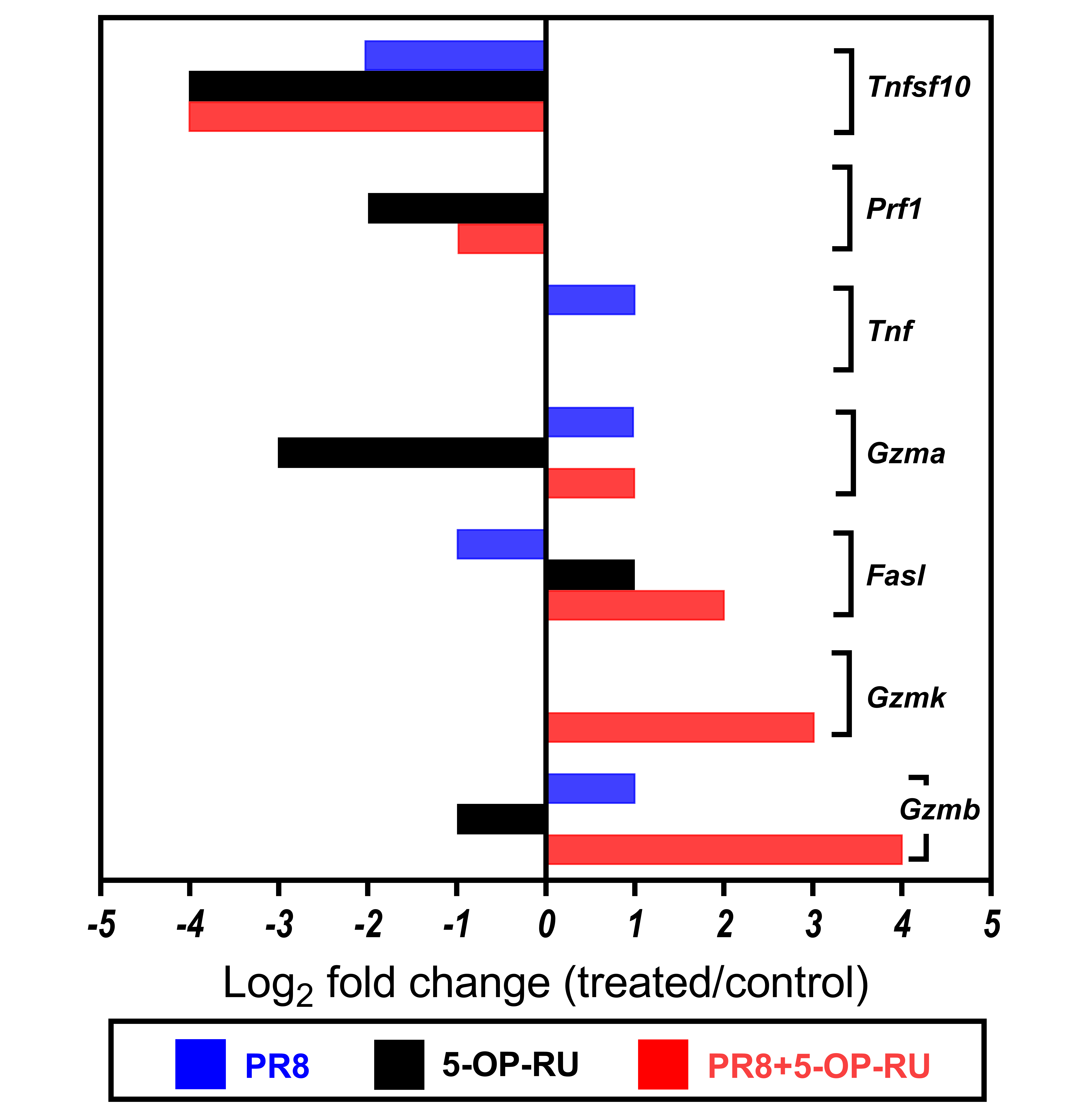

Supplement: S26 Fig — B6-MAITCAST mice were injected with PBS (n = 10), PR8 (n = 10), 5-OP-RU (n = 10), or PR8 plus 5-OP-RU (n = 5). After 3 days, pulmonary MAIT cells were purified, pooled and assessed by real-time PCR for their transcript levels of listed molecules. Gene expression fold changes were determined for each cohort compared with the PBS-injected (control) cohort as described in Materials and Methods. (TIF) [file ppat.1011485.s029.tif]
